# Four- and five-coordinate nickel(ii) complexes bearing new diphosphine–phosphonite and triphosphine–phosphite ligands: catalysts for *N*-alkylation of amines†

**DOI:** 10.1039/d1ra08961g

**Published:** 2022-02-03

**Authors:** Dipankar Panigrahi, Munmun Mondal, Rohit Gupta, Ganesan Mani

**Affiliations:** Department of Chemistry, Indian Institute of Technology Kharagpur Kharagpur 721 302 India gmani@chem.iitkgp.ac.in +91 3222 282252 +91 3222 282320

## Abstract

The reaction of Ph_2_PCH_2_OH with PhPCl_2_ and PCl_3_ in the presence of Et_3_N afforded new phosphonite compounds PhP(OCH_2_PPh_2_)_2_1 and P(OCH_2_PPh_2_)_3_2, respectively. The reaction between 1 and [NiCl_2_(DME)] in dichloromethane gave the five-coordinate complex [NiCl_2_(1-κ^3^*P*,*P*,*P*)] 3. Conversely, 1 reacts with [NiCl_2_(DME)] in the presence of NH_4_PF_6_ in dichloromethane to yield the four coordinate ionic complex [NiCl(1-κ^3^*P*,*P*,*P*)][PF_6_] 4. The reactions between 1, [NiCl_2_(DME)] and KPF_6_ in the presence of RNC (R = Xylyl, ^*t*^Bu and ^i^Pr) in dichloromethane yielded the five coordinate monocationic [NiCl(1-κ^3^*P*,*P*,*P*)(RNC)][PF_6_] (R = Xylyl) and dicationic [Ni(1-κ^3^*P*,*P*,*P*)(RNC)_2_][PF_6_]_2_ (R = ^*t*^Bu and ^i^Pr) complexes, respectively. The analogous reaction of 2 with [NiCl_2_(DME)] in the presence of KPF_6_ gave complex [NiCl(2-κ^4^*P*,*P*,*P*,*P*)][PF_6_], 8. The structures of all complexes were determined by single crystal X-ray diffraction studies and supported by spectroscopic methods. To demonstrate their catalytic application, *N*-alkylation reactions between primary aryl amines, benzyl and 4-methoxy benzyl alcohols were found to proceed smoothly in the presence of 2.5 mol% of complexes bearing ligand 1 and <0.5 mmol of KOBu^*t*^ in toluene at 140 °C. The C–N coupled products were formed in very good yields. Its substrate scope includes sterically encumbered, heterocyclic amines and aliphatic alcohol.

## Abstract 2

A series of nickel(ii) complexes supported by the new tridentate P3 and tetradentate P4 ligands act efficiently as catalysts for the *N*-alkylation of primary amines with alcohols.

## Graphical abstract

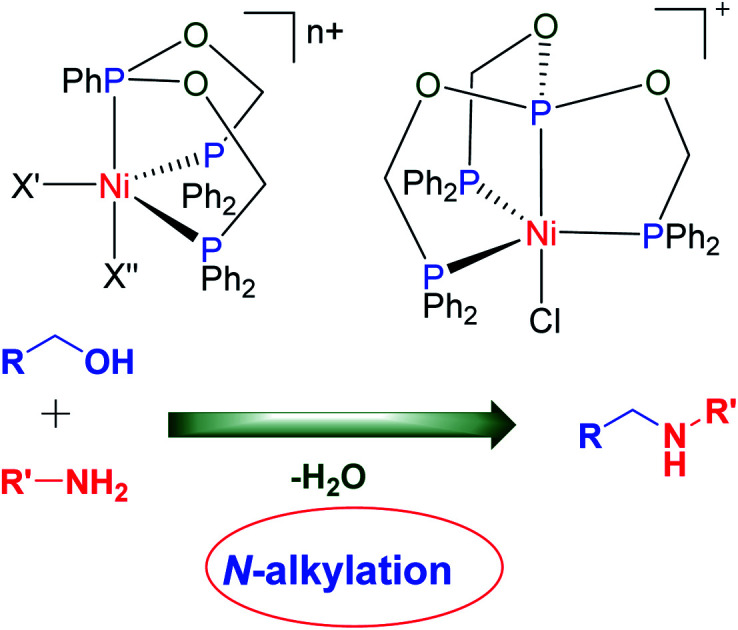

## Introduction

**Chart 1:**
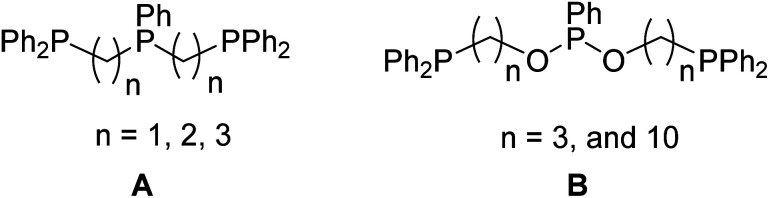
The reported linear tridentate P3 ligands.

Monophosphine and diphosphine ligands have found profound applications in homogeneous catalysis reactions.^1^ Next to them, tridentate and other multidentate ligands have also received attention and been used largely in the coordination chemistry.^2^ Compared to other ligands bearing ligating atoms such as C, N, O, the stereoelectronic factor of phosphine ligands can readily be changed as the nature of substituents on the phosphorus atom and the linking alkyl or aryl groups are changed.^3^ One of them is the linear tridentate phosphine ligand (type A) shown in Chart 1, in which the ligand with *n* = 2 has been widely used to build metal clusters,^4^ activate dinitrogen^5^ and to prepare low-valent complexes^6^ and in catalysis studies.^7^ On the contrary, the linear tridentate P3 ligands containing the phosphonite arm as the central anchoring atom (type B with *n* = 3 and 10) have been scarcely studied.^8^ Further, to our surprise, the type B ligand with *n* = 1 has not been reported to date.

The type A ligand where *n* = 2 was known to form five coordinate Ni(ii) complexes with distorted square pyramidal (SPy) or trigonal bipyramidal (TBP) geometries.^9^ Likewise, the unknown type B with *n* = 1 is a pincer-like terdentate ligand with flexible linkages connecting to the central non-aromatic anchoring atom. Its pendant phosphine arms can occupy equatorial positions in TBP (with P–Ni–P (*β* angle) < 140°), while the central phosphonite arm occupies the axial position with a distortion. In case of SPy, all three phosphorus atoms can occupy the square plane with a distortion. In general, complexes containing strongly coordinated flexible framework ligands are favorable for catalysis reactions as the geometry around the metal atom gets perturbed when a substrate binds which then undergoes transformations.

Catalysis by nickel metal complexes is voluminous and nickel catalysts have been described as “spirited horse”,^10^ because of several efficient catalysis reactions such as olefin oligomerization,^11^ C–H functionalization,^12^ activation of small molecules,^13^ C–C, and C–N coupling reactions^14^ and of their quite different mechanistic pathways. Of these, we have chosen the substitution of the primary amine hydrogen by the alkyl group of an alcohol, that is, the *N*-alkylation of amine by the hydrogen borrowing method, an atom efficient, cost effective, environmentally benign (though high temperature involved) and versatile process,^15^ in contrast to the conventional methods involving harmful and pricey alkyl halide, sulfonate or sulfate.^16^ Hence, in addition to the other C–N bond formation reactions such as hydroamination,^17^ and Buchwald–Hartwig coupling^18^ among others,^19^ this catalytic process have attracted attention of researchers from both academia and industrial sectors for a long time for which both homogeneous and heterogeneous^20^ catalytic processes have been reported.^21^ We are interested in homogeneous catalysis using well-defined, new molecular metal complexes. Among metal complexes containing Ru,^22^ Rh,^23^ Ir,^24^ Au,^25^ Mn,^26^ Co,^27^ Fe,^28^ Pd^29^ metals, nickel complexes catalyzed reactions remains attractive because of the above mentioned reasons in addition to their salts being cheaper. Hence, several efficient nickel complexes have been reported to catalyze *N*-alkylation reactions.^30^ However, to the best of knowledge, there is no report of *N*-alkylation catalyzed by nickel complex bearing tridentate P3 ligand such as PhP(CH_2_CH_2_PPh_2_)_2_. Herein, we report the synthesis, and structural characterizations of nickel complexes supported by new tridentate and tripodal tetradentate phosphorus containing ligands along with *N*-alkylation reactions catalysed by four and five coordinate nickel(ii) complexes bearing the tridentate P3 ligand.

## Results and discussion

### Synthesis of phosphonite ligands

**Scheme 1:**
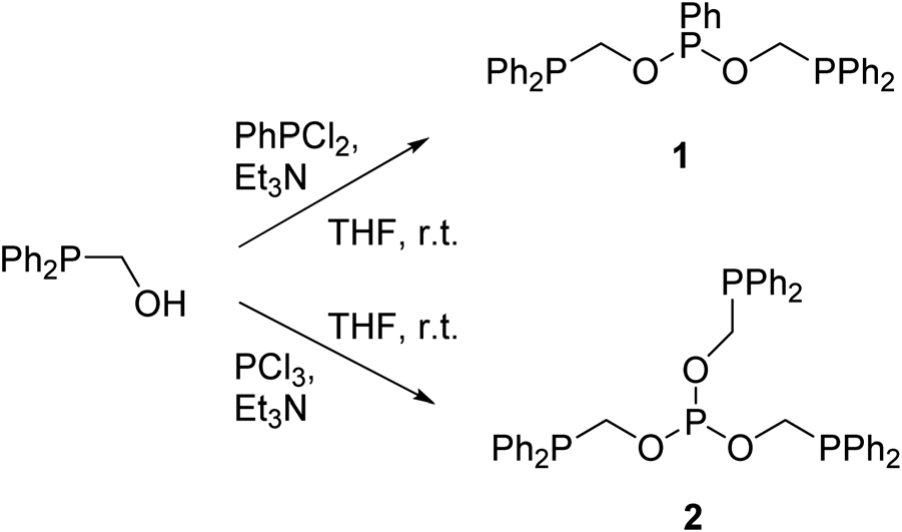
Synthesis of new tridentate and tetradentate ligands 1 and 2.

The reaction between PhPCl_2_ and Ph_2_PCH_2_OH in the 1 : 2 molar ratio in the presence of Et_3_N at room temperature afforded the new diphosphine–phosphonite ligand PhP(OCH_2_PPh_2_)_2_1 as a colorless viscous oil in an excellent yield (97%) (Scheme 1). The analogous reaction with PCl_3_ in the presence of Et_3_N gave yet another new triphosphine–phosphite ligand P(OCH_2_PPh_2_)_3_2 as a viscous liquid in 81% yield.

Compound 1 and 2 are air-sensitive compounds and soluble in CH_2_Cl_2_, CHCl_3_, CH_3_CN, toluene, and THF. Its ^1^H NMR spectrum showed two multiplets at *δ* = 4.20 and 4.39 ppm for the methylene protons owing to the spin system of ABMX (A = B = H; M = X = P). This is similar to the signal of methylene protons in bis(2-oxazolin-2-ylmethyl)phenylphosphine.^31^ Conversely, these couplings were not seen for the methylene protons in 2 which appeared as a broad multiplet in the ^1^H NMR spectrum, indicating that it is a symmetrical molecule. The ^31^P{^1^H} NMR spectrum of 1 and 2 in CDCl_3_ showed two singlets for the two different phosphorus atoms [−13.4 (PPh_2_) and 162.9 ppm (PPh) for 1 and −13.8 (PPh_2_) and 140.1 ppm (P) for 2]. These chemical shift values appear in the deshielded region except the peak at 140.1 ppm of 2 relative to the values of reported compounds: PhP{O(CH_2_)_3_PPh_2_}_2_ [−15.96 (PPh_2_) and 156.24 (PPh)] and PhP{O(CH_2_)_10_PPh_2_}_2_ [−16.02 (PPh_2_) and 155.36 (PPh)].^8^

### Metalation and structural characterizations

**Scheme 2:**
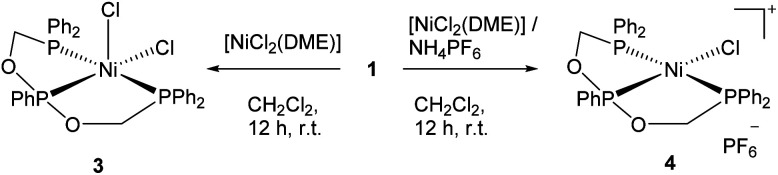
The five- and four-coordinate nickel(ii) complexes of ligand 1.

The equimolar reaction between 1 and [NiCl_2_(DME)] in dichloromethane at room temperature followed by crystallization afforded the neutral five-coordinate nickel(ii) complex 3 as a green crystalline compound. The same reaction in the presence of NH_4_PF_6_ yielded the four-coordinate ionic complex 4 with the PF_6_^−^ anion as orange-red crystals (Scheme 2). Both complexes are soluble in common organic solvents such as CH_2_Cl_2_, CHCl_3_, CH_3_CN, and THF.

The ^1^H NMR spectra of complex 3 and 4 in CDCl_3_ featured a well separated broad AB multiplets for the diastereotopic methylene protons owing to the orientation of the phenyl ring of the central phosphorus atom on one side of the nickel square plane (see below). The ^13^C NMR spectrum of 3 displayed a broad multiplet at *δ* 72.8 ppm owing to the coupling with two different phosphorus atoms. The ^31^P{^1^H} NMR spectra of 3 and 4 showed two signals corresponding to the two different coordinated phosphorus atoms in the structure [for 3, *δ* 50.9 (PPh_2_) and 209.8 ppm; for 4, 60.1 (PPh_2_) and 218.8 ppm]. In contrast to the free ligand, in both cases, the signal of PPh_2_ groups appears as a doublet owing to the coupling with the central phosphorus atom, while the central phosphorus appears as a broad singlet, and these signals are well shifted down field relative to the free ligand value.

**Fig. 1:**
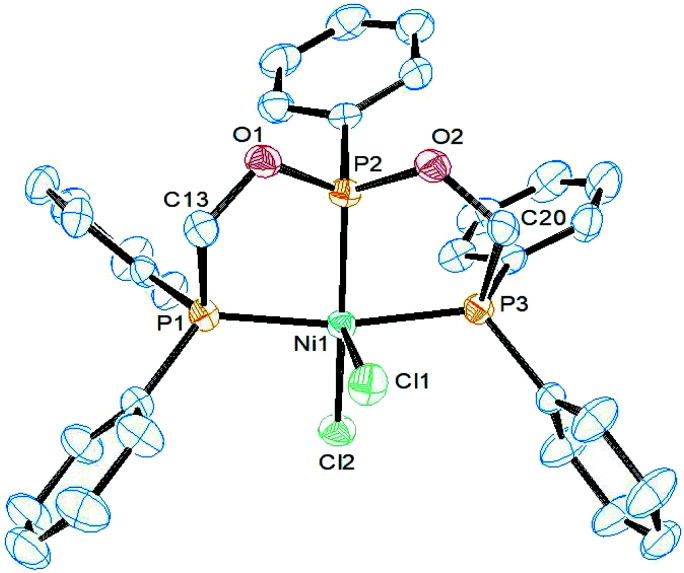
ORTEP diagram of the X-ray structure of 3 (50% displacement ellipsoids). Hydrogen atoms and the solvent of crystallization CH_2_Cl_2_ are omitted for clarity. Selected bond lengths (Å) and bond angles (°): Ni1–P2 2.1106(10), Ni1–P3 2.1793(10), Ni1–P1 2.1985(10), Ni1–Cl2 2.2403(10), Ni1–Cl1 2.5320(10), P2–O1 1.602(2), P2–Ni1–P3 82.66(4), P2–Ni1–P1 84.51(4), P3–Ni1–P1 164.78(4), P2–Ni1–Cl2 153.30(4), P3–Ni1–Cl2 95.58(4), P1–Ni1–Cl2 99.55(4), P2–Ni1–Cl1 97.28(4), P3–Ni1–Cl1 85.36(3), P1–Ni1–Cl1 88.19(3), Cl2–Ni1–Cl1 109.17(4).

The structure of complex 3 was determined by single crystal X-ray diffraction study. It crystallizes in the triclinic *P*2_1_/*c* space group and the asymmetric unit constitutes one molecule of 3 along with CH_2_Cl_2_ as the solvent of crystallization. An ORTEP diagram along with selected bond lengths and angles is given in Fig. 1. In the structure, the nickel atom is coordinated by ligand 1 adopting the κ^3^-*P*,*P*,*P* coordination mode and two chlorine atoms. The geometry around the nickel atom is best described as a distorted square pyramidal in which the basal plane is formed by the three phosphorus and one chlorine atoms. The five-coordinate *τ*_5_ parameter for this complex is 0.20 (*τ*_5_ = 1 for trigonal bipyramid and *τ*_5_ = 0 for square pyramid).^32^ This structure is similar to the structure of [NiCl_2_(PhP{CH_2_CH_2_PPh_2_}_2_)] (C) with *τ*_5_ = 02.^33^ However, metric parameters are different owing to the presence of oxygen link in ligand 1. The Ni–P_central_ distance of 2.111(1) Å is shorter than 2.135(1) Å in the reported complex C owing the strong π-acceptor nature of the phosphonite group in 1. The chlorine atom Cl2 *trans* to this central phosphorus atom is bonded at a relatively longer distance of 2.240(1) Å than 2.199(1) Å found in C, and this can also be attributed to the high *trans* influence of phosphorus than the chlorine atom. In addition, the axial Ni–Cl1 distance (2.532(1) Å) is longer than the basal Ni–Cl2 distance and is shorter than 2.652(1) Å found in C. The P1–Ni–P3 (164.8(1)°) and P2–Ni–Cl2 (153.3(1)°) angles are lower than those (168.7(1)° and 169.9(1)°) found in C and as a result, the nickel atom is located 0.205 Å above the mean square plane in 3.

**Fig. 2:**
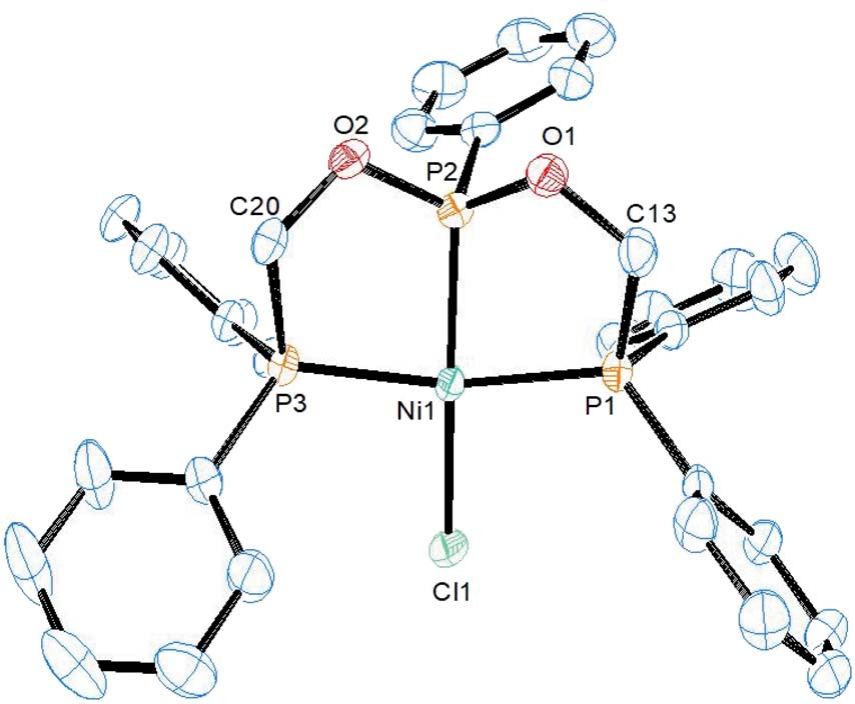
ORTEP diagram of the X-ray structure of 4 (50% displacement ellipsoids). Hydrogen atoms and PF_6_^−^ are omitted for clarity. Selected bond lengths (Å) and bond angles (°): P1–Ni1 2.1978(6), P2–Ni1 2.1067(7), P3–Ni1 2.1949(6), Ni1–Cl1 2.1685(7), P1–Ni1–P2 82.60(3), P2–Ni1–P3 84.47(2), P1–Ni1–Cl1 97.14(3), P3–Ni1–Cl1 97.86(3), P1–Ni1–P3 161.67(3), P2–Ni1–Cl1 169.90(3).

The structure of complex 4 was also determined by single crystal X- ray diffraction study. It crystallizes in the triclinic *P*1̄ space group and the asymmetric unit consists of both the cationic nickel center and the PF_6_^−^ anion. An ORTEP diagram along with selected bond lengths and angles is given in Fig. 2. The X-ray structure revealed the formation of a distorted square planar cationic Ni(ii) complex containing one ligand 1 and one chlorine atom which is charge neutralized by PF_6_^−^ anion. Ligand 1 adopts the κ^3^-*P*,*P*,*P* coordination mode and renders two fused five-membered rings about the nickel atom. The five-membered rings are puckered and the phenyl group attached to the central phosphorus P2 is oriented approximately perpendicular to the nickel square plane by which the tetrahedral angle around that phosphorus is maintained. The four-coordinate geometry index value *τ*_4_ is 0.20 (*τ*_4_ = 1 for tetrahedron and *τ*_4_ = 0 for square planar)^32^ and the nickel atom lies 0.017 Å above the mean square plane defined by P1, P2, P3 and Cl atoms. These two values are slightly different from the values (0.18 and 0.064(1) Å, respectively) found in the closely related structure [NiCl(PhP{CH_2_CH_2_PPh_2_}_2_)]^+^(D)^34^ and are attributed to the relatively smaller size of the oxygen atom in ligand 1 than the carbon atom of the ligand in D. The P1–Ni–P3 angle of 161.7(1)° and the P2–Ni1–Cl1 angle of 169.9(1)° are not differing much from the analogous angles (162.4(1)° and 171.6(1)°) in the reported complex D. The Ni–Cl bond distance of 2.1685(7) Å is close to 2.169(3) Å in complex D. The central phosphorus P2–Ni distance of 2.1067(7) Å is slightly shorter than the other two P–Ni distances (2.1978(6) Å and 2.1949(6) Å) owing to the stronger *trans* influence of phosphorus than the chlorine atom and to the nature of atoms (two oxygens and one aryl carbon to the central P2; two aryl and one alkyl carbons to P1 and P3) attached to the phosphorus atom. Similar trend has been observed in the reported four coordinate Ni(ii) square planar complexes such as D, [Ni(SR)(PhP{CH_2_CH_2_PPh_2_}_2_)]^+^(R = Et, ^*t*^Bu, Cy,^35^ Bn^36^ and Ph^37^) and in the five coordinate square pyramidal Ni(ii) complex C.

**Scheme 3:**
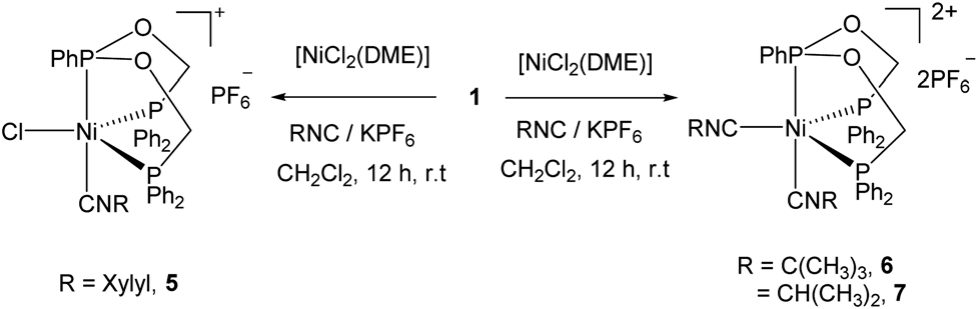
Synthesis of the five-coordinate Ni(ii) complexes 5–7.

The addition of 2,6-dimethylphenyl isocyanide and KPF_6_ to the reaction mixture of ligand 1 and [NiCl_2_(DME)] in CH_2_Cl_2_ followed by crystallization afforded the five coordinate complex 5 containing one xylyl isocyanide in good yield. However, the analogous reactions with isopropyl- or *tert*-butyl isocyanide yielded different five coordinate dicationic nickel(ii) complexes 6 and 7 containing two isonitrile molecules after crystallization of the reaction mixture (Scheme 3). Complexes are soluble in CH_2_Cl_2_, CHCl_3_, THF and insoluble in toluene.

In the ^1^H NMR spectra of complexes 5–7, the diastereotopic methylene protons of ligand display varying multiplets owing to the formation of five coordinate complex in which the phenyl group of the central phosphorus atom lies perpendicular to the equatorial plane and their fluxional nature in CDCl_3_ solution at room temperature. The two multiplets of the methylene protons in the free ligand is changed to two broad doublets and one broad singlet in the ^1^H NMR spectrum of complex 5. In the case of complex 7, it appeared a single broad multiplet. Conversely, broad doublets and multiplets two each were observed for the ^*t*^BuNC coordinated complex 6. This indicates that among these complexes the structure of complex 6 remains more rigid in solution owing to the steric bulk of the ^*t*^Bu group. This is further supported by the ^31^P{^1^H} NMR spectrum of 6 showed a doublet and a triplet, in contrast to the not well resolved spectra of 5 and 7 displaying a doublet for the PPh_2_ and a broad signal for the PPh phosphorus atom.

**Fig. 3:**
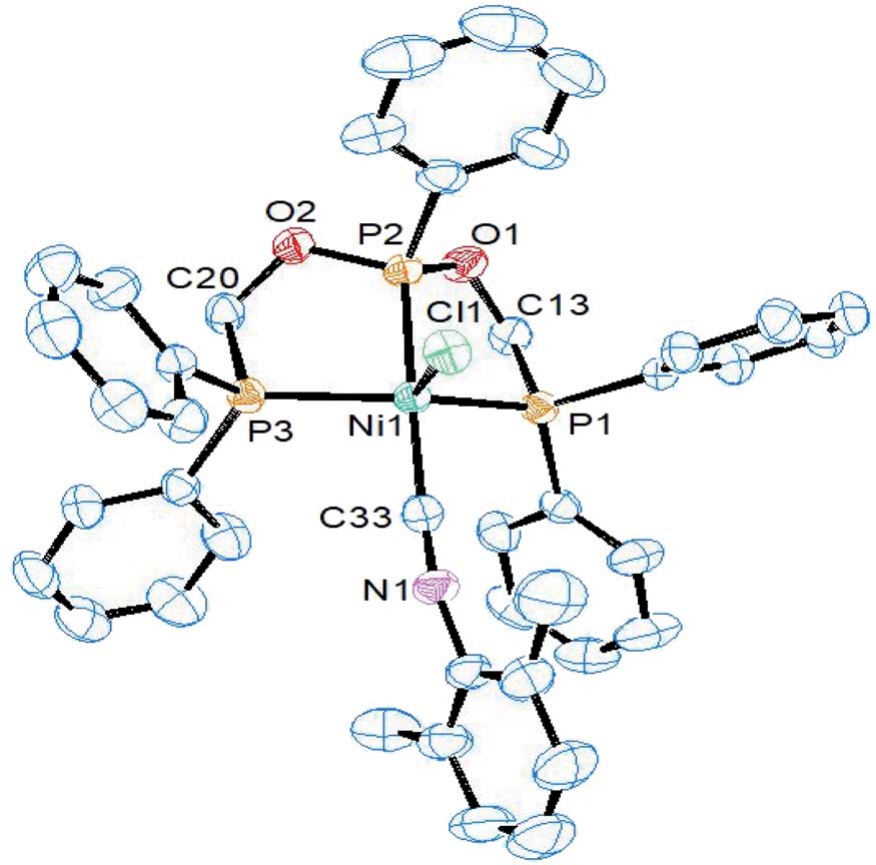
ORTEP diagram of the X-ray structure of 5 (50% displacement ellipsoids). Hydrogen atoms and PF_6_^−^ are omitted for clarity. Selected bond lengths (Å) and bond angles (°): P1–Ni1 2.1953(11), P2–Ni1 2.1459(12), P3–Ni1 2.2118(12), Ni1–C33 1.841(5), Ni1–Cl1 2.3522(12), P1–Ni1–P2 83.58(4), P2–Ni1–P3 85.03(4), P1–Ni1–C33 90.91(13), P1–Ni1–Cl1 116.05(4), P2–Ni1–C33 172.04(14), P2–Ni1–Cl1 95.38(5), P3–Ni1–C33 95.65(13), P3–Ni1–Cl1 103.05(4), P1–Ni1–P3 140.06(4).

Complex 5 crystallizes in the monoclinic space group *P*2_1_/*c* and the asymmetric unit contains both the cationic nickel(ii) center and the PF_6_^−^ anion. As in the previous structures, the triphosphine ligand adopts the κ^3^-*P*,*P*,*P* coordination mode and the five coordination around the nickel atom is completed by the chloride and XylylNC ligands (Fig. 3). The central phosphorus atom and the xylyl isocyanide occupy the axial positions, while the terminal phosphorus and chlorine atoms remain in the equatorial positions. Among the angles around the nickel atom, the axial P2–Ni–C33 angle of 172.0(1) is the largest value and the equatorial P1–Ni–P3 angle of 140.1(1) is the second largest one. The other two basal angles P1–Ni–Cl (116.0(1)°) and P3–Ni–Cl (103.0(1)°) are smaller by which the steric repulsion between the two diphenylphosphine groups is minimized. The five coordinate *τ*_5_ value is 0.53, suggesting that the structure lies in the middle of two extreme ideal structures - trigonal bipyramidal (*τ*_5_ = 1) and square pyramidal (*τ*_5_ = 0) and is best described as a distorted trigonal bipyramidal geometry. The variation in the Ni–P distances (2.1953(11), 2.1459(12) and 2.2118(12) Å) are similar to complex 3 and also to those found in the reported five-coordinate Ni(ii) complexes: [Ni(CN)_2_(PhP{CH_2_CH_2_PPh_2_}_2_)],^38^ [NiCl(dppp)(XylNC)_2_]^+^,^39^ [Ni{1,2-bis(bis(hydroxypropyl)phosphino)ethane}_2_Cl]^+^,^40^ [NiCl{bis(dimethylphosphino)ethane}_2_]^+^,^41^ [NiCl(*P*,*P*′-diphenyl-1,4-diphosphacyclohexane)_2_]^+^,^42^ and [NiCl(1,5-diphenyl-1,5-diphosphacyclooctane)_2_]^+^.^43^

The IR spectrum of complex 5 showed the *ν*(NC) stretching band at 2169 cm^−1^ which is close to 2174 cm^−1^ in [Ni(triphos)(XylylNC)]^2+^,^44^ and remains lower than 2198 cm^−1^ reported for [NiBr_2_(XylylNC)_2_].^45^ However, it is higher than the solution IR band of the free XylylNC (2117 cm^−1^),^46^ indicating that it acts as rather σ-donor. This is supported by the C33–N1 distance of 1.149(5) Å which is close to 1.161(1) Å found in the free XylylNC.^46^ The Ni–C_XylylNC_ bond length of 1.841(5) Å is similar to those found in the five coordinate Ni(ii) complexes such as [NiCl(dppp)(XylNC)_2_][PF_6_] (1.849(8) Å) and [NiCl(PPh_3_)_2_(XylNC)_2_][PF_6_] (1.84(2) Å),^39^ and [NiCl{C(NCH_2_PPh_2_)_2_(CH_2_)_3_-κ^3^*P*,*C*,*P*′}(XylylNC)][PF_6_] (1.831(2) Å).^47^ The Ni–Cl distance of 2.352(1) Å is rather far from the sum of the covalent radii of Ni (1.24 Å) and Cl (1.02 Å) atoms; however, it falls within the sum of the van der Waals radii (Ni = 1.63 Å and Cl = 1.75 Å) and is similar to the distances found in the reported complexes.^39,41–43,47,48^

**Fig. 4:**
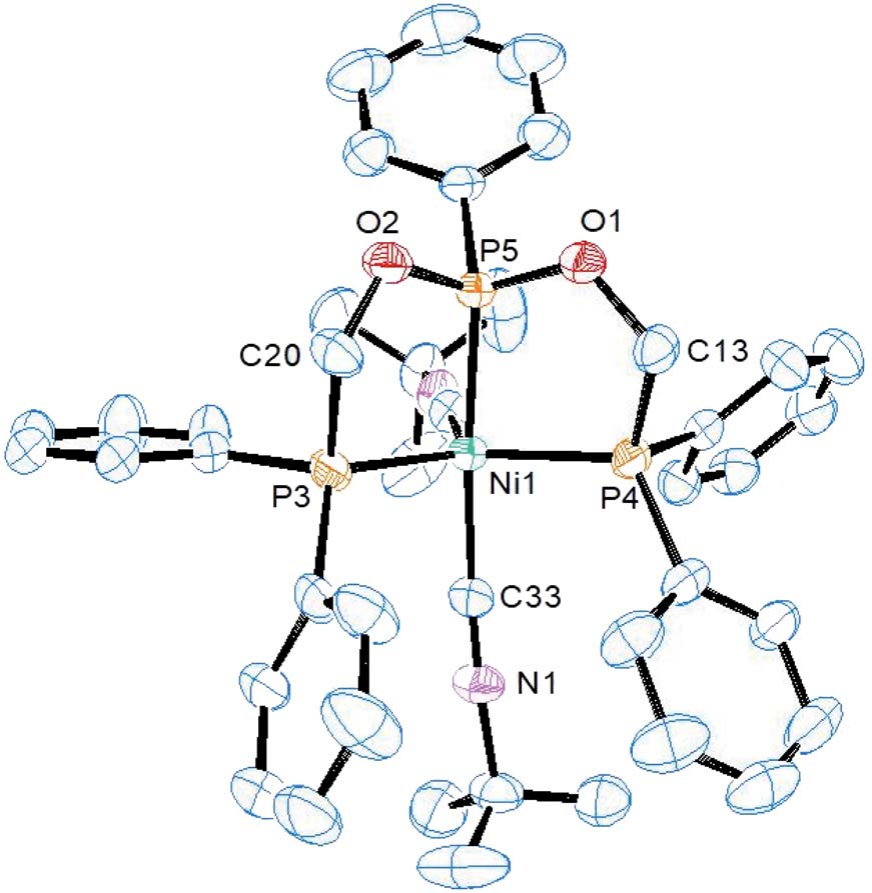
ORTEP diagram of the X-ray structure of 6 (50% displacement ellipsoids). Hydrogen atoms, PF_6_^−^ and the solvent of crystallization CH_2_Cl_2_ are omitted for clarity. Selected bond lengths (Å) and bond angles (°): Ni1–P5 2.1518(11), Ni1–P3 2.2234(12), Ni1–P4 2.2255(12), Ni1–C33 1.866(4), Ni1–C38 1.955(5), P5–Ni1–P3 84.98(4), P5–Ni1–P4 84.70(4), P3–Ni1–P4 131.95(5), C33–Ni1–P5 169.79(14), C38–Ni1–P5 95.19(13), C33–Ni1–C38 94.69(18), C33–Ni1–P3 92.74(14), C38–Ni1–P3 117.43(13), C33–Ni1–P4 89.49(14), C38–Ni1–P4 110.17(13).

**Fig. 5:**
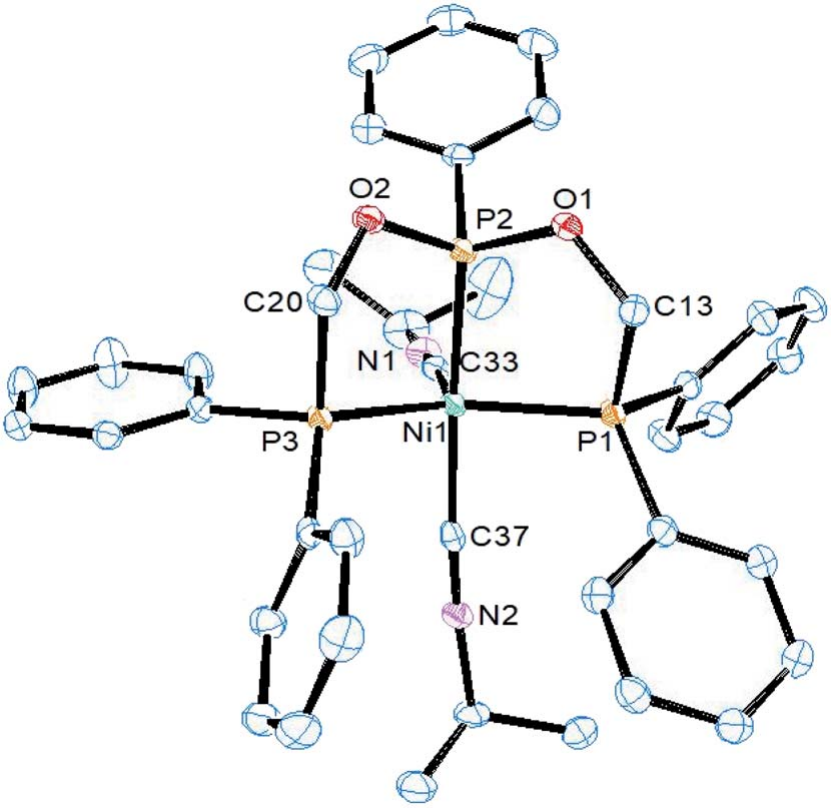
ORTEP diagram of the X-ray structure of 7 (50% displacement ellipsoids). Hydrogen atoms, PF_6_^−^ and the solvent of crystallization CH_2_Cl_2_ are omitted for clarity. Selected bond lengths (Å) and bond angles (°): P1–Ni1 2.2054(7), P2–Ni1 2.1447(6), P3–Ni1 2.2081(6), Ni1–C33 1.915(2), Ni1–C37 1.850(2), P1–Ni1–P2 85.18(2), P2–Ni1–P3 85.67(2), P1–Ni1–C33 112.99(7), P1–Ni1–C37 90.64(7), P2–Ni1–C33 93.80(7), P2–Ni1–C37 173.94(7), P3–Ni1–C33 120.01(7), P3–Ni1–C37 93.34(7), P1–Ni1–P3 126.63(2).

Complex 6 and 7 crystallize in the monoclinic space group *P*2_1_/*n* and their asymmetric units consist of the whole molecule and one dichloromethane. ORTEP views along with selected bond lengths and angles are shown in Fig. 4 and 5, respectively. The X-ray structure revealed a distorted trigonal bipyramidal geometry around the dicationic nickel ion which is charge neutralized by two PF_6_^−^ anions. While the terminal phosphorus arms of ligand and one isocyanide occupy the equatorial positions, the central phosphorus and another isocyanide occupy the axial positions. The basal P1–Ni1–P3 angles in both complexes (132.04(4) and 126.63(2)°) are higher than the other two basal angles. The five-coordinate geometry index *τ*_5_ = 0.79 for 6 and 0.63 for 7 complex, suggesting that the ^*t*^BuNC ligand restraints the geometry more toward TBP than does the ^i^PrNC ligand. As a result, a better resolved ^1^H NMR spectra was obtained for ^*t*^BuNC complex 5. As observed in the previous complexes, the central P–Ni distance is slightly shorter than the other two P–Ni distances. The axial isocyanide C–Ni distance is shorter than the equatorial C–Ni distance in both complexes. This is similar to [Ni(CN)_2_(PhP{CH_2_CH_2_PPh_2_}_2_)] in which the cyanide ions are present in both positions. The isocyanide C–N distances are similar to that in complex 5 (see above) and hence the isocyanide ligand in both complexes acts as a σ-donor rather than σ-donor–π-acceptor which is indicated by their *ν*(CN) bands being higher than the free isocyanide value.

**Scheme 4:**
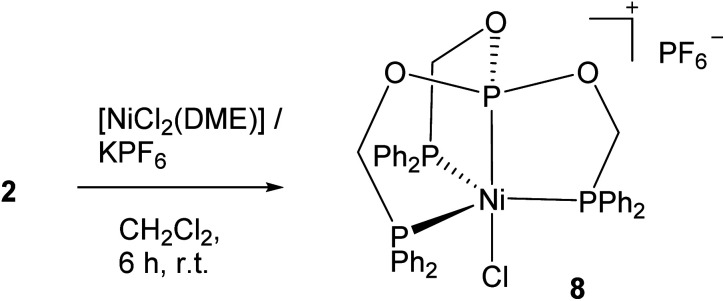
Synthesis of Ni(ii) complex of ligand 2.

The reaction of the tripodal ligand 3 with [NiCl_2_(DME)] in dichloromethane at room temperature afforded complex 8 in about 45% yield as purple crystals obtained after crystallization of the reaction mixture (Scheme 4). Complex is well soluble in CH_2_Cl_2_, THF and sparingly soluble in chloroform. In the ^1^H NMR spectrum of 8 in DMSO-*d*_6_, a down field shifted doublet appeared for the diastereotopic methylene protons in contrast to the broad multiplet observed for the free ligand. Similarly, in the ^31^P{^1^H} NMR spectrum, the presence of two types of the coordinated phosphorus atom is shown by the down field shifted signals one at *δ* 46.3 ppm as a doublet for the terminal phosphorus atoms and the other at 204.0 ppm as a multiplet for the central phosphorus atom.

**Fig. 6:**
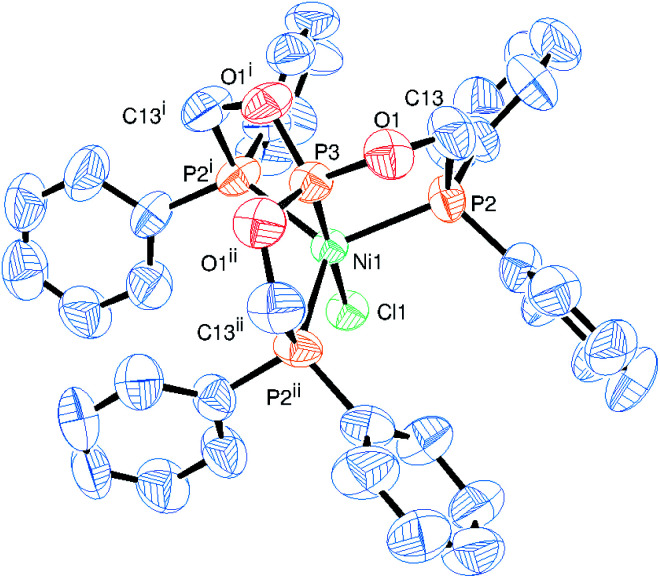
ORTEP diagram of the X-ray structure of 8 (50% displacement ellipsoids). Hydrogen atoms, and PF_6_^−^ are omitted for clarity. Selected bond lengths (Å) and bond angles (°): P2–Ni1 2.259(2), P3–Ni1 2.063(4), Cl1–Ni1 2.218(3), O1–P3 1.581(6), F1–P1 1.568(9), F2–P1 1.579(10), P3–Ni1–Cl1 180.00(13), P3–Ni1–P2 83.74(7), Cl1–Ni1–P2 96.26(7), P2–Ni1–P2^i^ 118.83(3). Symmetry transformations used to generate equivalent atoms: (i) 1 − *y* + 1/2, −*z* + 1, *x* + 1/2, (ii) *z* − 1/2, −*x* + 1/2, −*y* + 1, (iii) −*z* + 1, *x* + 1/2, −*y* + 3/2, (iv) *y* − 1/2, −*z* + 3/2, −*x* + 1.

Complex 8 crystallizes in the cubic *P*2_1_3 space group and the asymmetric unit contains one arm of the tripodal ligand coordinated to the ‘Ni–Cl’ unit alone with a part of the anion (Fig. 6). The whole molecule was generated by the symmetry generation. Ligand adopts the *κ*^4^-*PPPP* coordination mode with the nickel atom containing one chlorine atom forming the cation, which is charge neutralized by the PF_6_^−^ anion. The nickel atom adopts a slightly distorted TBP geometry with the P_ax_–Ni–Cl angle of 180.0°. The other angles P_eq_–Ni–P_eq_ (118.8(1)°), P_ax_–Ni–P_eq_ (83.7(1)°) and P_eq_–Ni–Cl (96.3(1)°) remain slightly deviating from the ideal TBP geometry. As observed in the previous structures, the central phosphorus nickel distance, that is, P_ax_–Ni of 2.063(4) Å is shorter than those of the terminal P_eq_–Ni distances (2.259(2) Å). As compared to the structure 3, this P_eq_–Ni distance is slightly longer, as none of the phosphorus atom is *trans* to each other as in 3. On the other hand, the phosphite phosphorus nickel distance (P_ax_–Ni) is shorter than the P–Ni bond distance of 2.1106(10) Å *trans* to the chlorine atom in complex 3 owing to the relatively stronger π-acceptor character of the central phosphorus atom in complex 8.

Further, this P_ax_–Ni distance is shorter than 2.137(2) Å in [(PP3)NiP(OMe)_3_]^2+^,^49^ 2.157(3) Å in [(PP3)Ni(SH)]^+^,^50^ 2.142(3) Å in [(PP3)NiI]^+^,^51^ and 2.163(2) Å in [(PP3)NiBr]^+^,^52^ which all contain similar tetradentate tripodal ligand P(CH_2_CH_2_PPh_2_)_3_ (PP3). The Ni–Cl distance of 2.218(3) Å lies within the range found in complex 3 and 4. The PF_6_^−^ anion assumes a regular octahedral geometry as the F–P–F *cis* and *trans* angles of 89.8(8)° and 176.3(8)°, respectively, are close to those in an ideal octahedron with almost the same P–F distances (1.568(9) and 1.579(10) Å).

### *N*-Alkylation catalysis

To test the catalytic *N*-alkylation of aromatic amine, 4-methoxyaniline, benzyl alcohol and complex 4 were chosen. The reaction between 4-methoxyaniline (0.5 mmol) and benzyl alcohol (1 mmol) in the presence of KOBu^*t*^ (0.4 mmol) and complex 4 (2.5 mol% with respect to amine) in toluene at 140 °C for 24 h afforded the C–N coupled product *N*-benzyl-4-methoxyaniline in 94% yield after column separation (Table 1, entry 1). When the complex loading was decreased to 1 mol% under the same conditions the yield of the product was decreased slightly to 84% (entry 2). Similarly, the decrease in loading of base by 0.25 mmol or increase by 1 mmol results in drastic decrease in the yield of product to 45 or 68% (entry 3 and 4). Under the same conditions, the reaction in the absence of complex 4 did not give the product in an appreciable quantity (entry 5), suggesting that the presence of complex is essential. In absence of base, the product was not formed (entry 6). Further, the yield of the product decreased to 56% in the presence of KOH (entry 7). Furthermore, when the reaction was carried out in open air reflux conditions, the imine product, *N*-(4-methoxyphenyl)-1-phenylmethanimine, was obtained in 53% yield. The low yield of this imine product is conceivable from the TLC analysis of the reaction mixture, showed a very less intense spot for the amine product along with a major intense spot for this imine product. This suggests the *in situ* formation of either nickel hydride or hydrogen gas, which reduces the imine product to amine. The analogous catalytic reactions in the presence of complex 3, 5 or 6 yielded the same product in a slightly lower yields (entries 8–10).

**Table 1:** *N*-Alkylation of 4-methoxyaniline with benzyl alcohol catalyzed by nickel(ii) complexes^a^

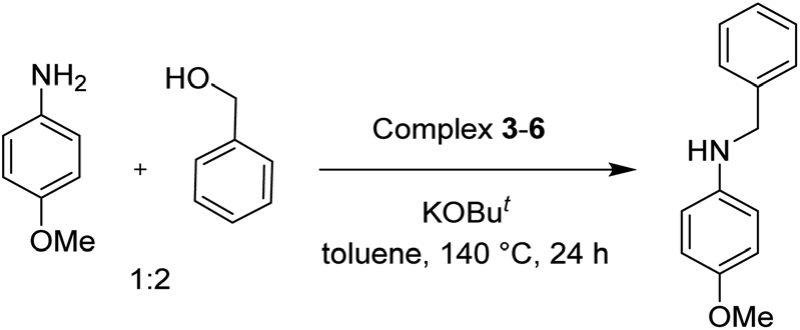			
Entry	Complex (mol%)	KOBu^*t*^ (mmol)	Yield^b^ (%)
1	4 (2.5)	0.4	94
2	4 (1.0)	0.4	84
3	4 (2.5)	0.25	45
4	4 (2.5)	1.0	68
5	—	0.4	Trace
6	4 (2.5)	0.0	0
7^c^	4 (2.5)	0.4	56
8	3 (2.5)	0.4	91
9	5 (2.5)	0.4	78
10	6 (2.5)	0.4	89

a Closed tube containing 1 mL of toluene and an oil bath temperature of 140 °C.

b Isolated yield.

c KOH as a base.

The conditions under which the product yield of 94% observed with complex 4 were applied to a few more reactions involving other aromatic amines containing different groups in the aromatic ring with benzyl alcohol and their details are given Table 2. With aniline or 4-bromoaniline the C–N coupled product yield was very good (92% and 88%, respectively). But the yield was decreased with 2,6-diisopropylaniline owing to the steric bulk of the two isopropyl groups in the aryl ring. Interestingly, above 90% yield was obtained with heterocycle amine (entry 4). The *N*-alkylation using an aliphatic alcohol ^*n*^BuOH was also observed but in less yield (37%, entry 5) because of the slow reaction and the presence of unreacted starting compounds. In addition, alkylation using 4-methoxybenzyl alcohol were also carried out under the same conditions used before and their products are shown in Table 3.

**Table 2:** *N*-Alkylation of different amines with benzyl alcohol catalyzed by complex 4^a^

Entry	Amine	Product	Yield^a^, %
1	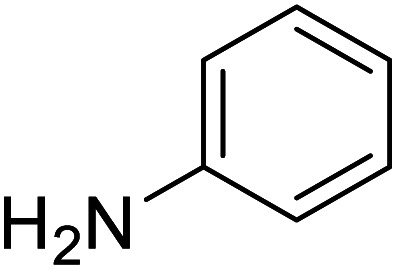	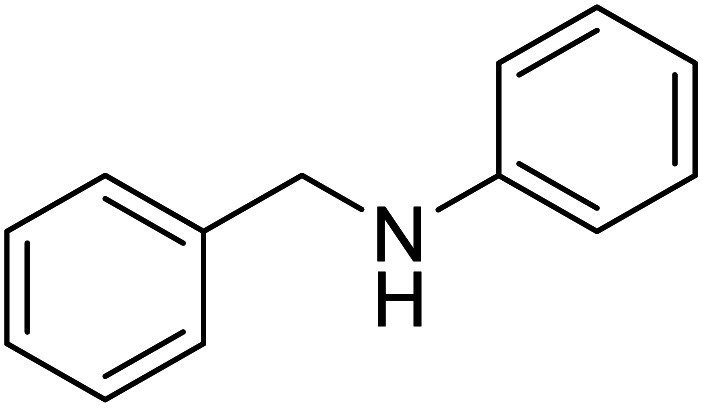	92
2	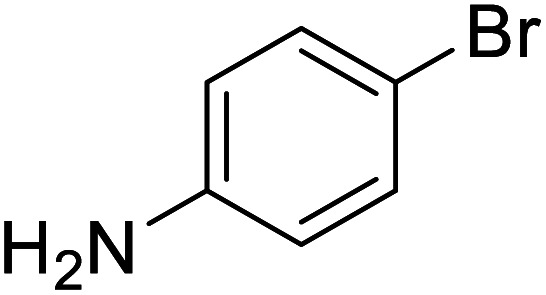	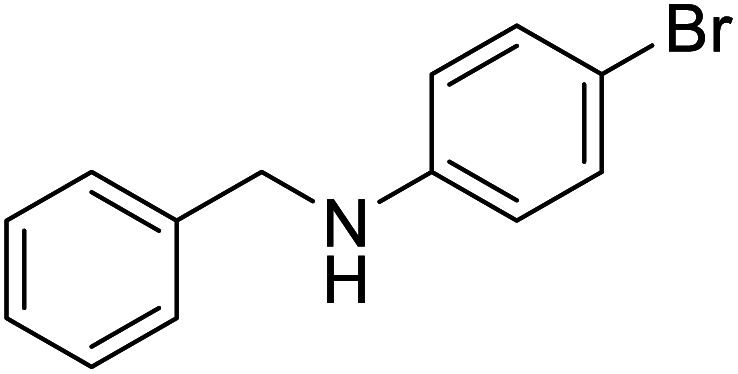	88
3	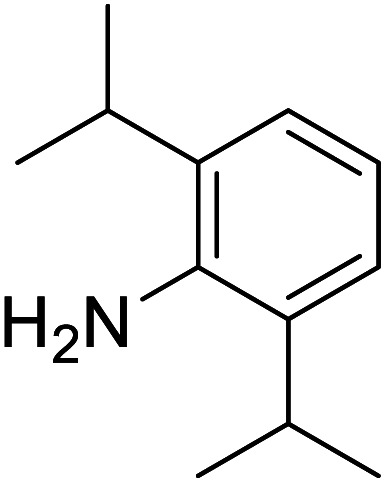	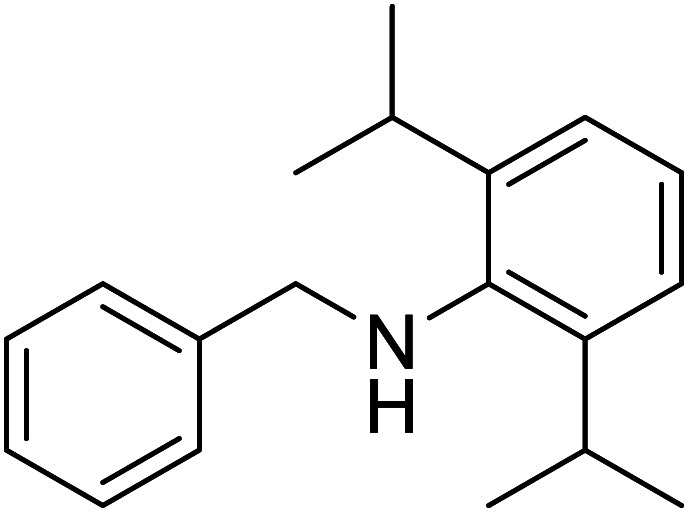	71
4	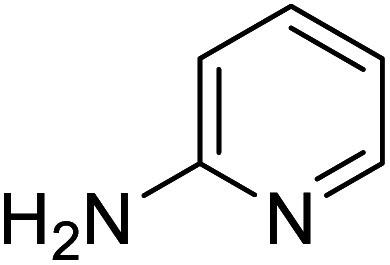	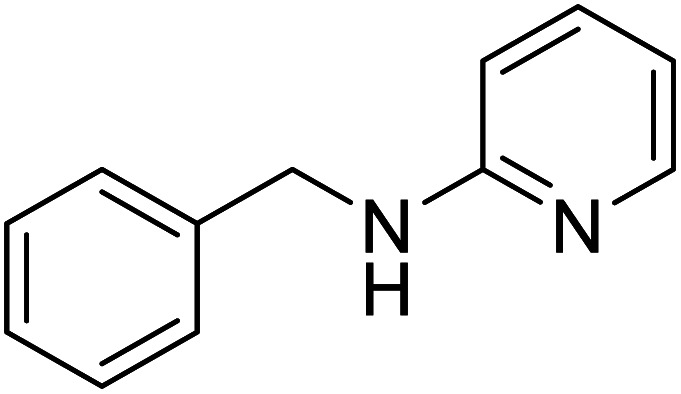	91
5^b^	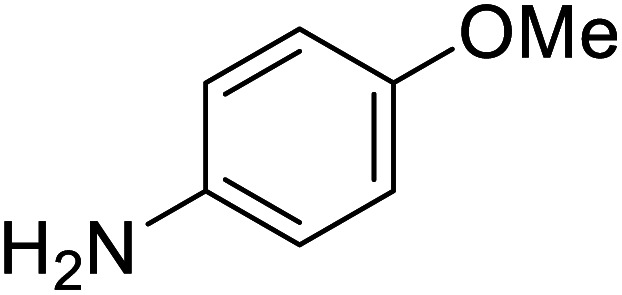	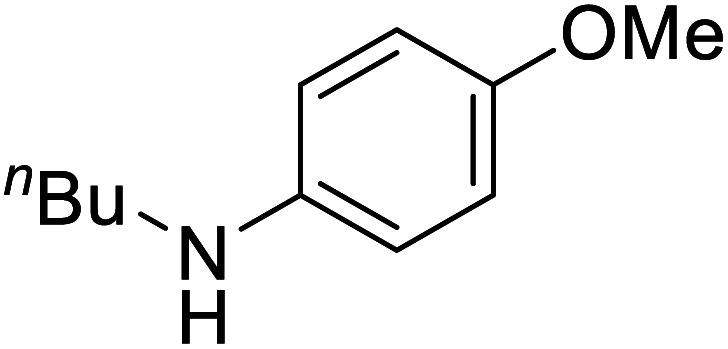	37

a Isolated yield.

b ^*n*^Butanol used.

**Table 3:** *N*-Alkylation of different amines with 4-methoxybenzyl alcohol catalyzed by complex 4^a^

Entry	Amine	Product	Yield^b^, %
1	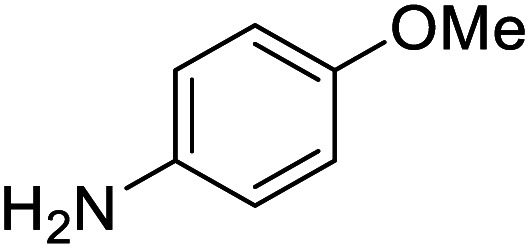	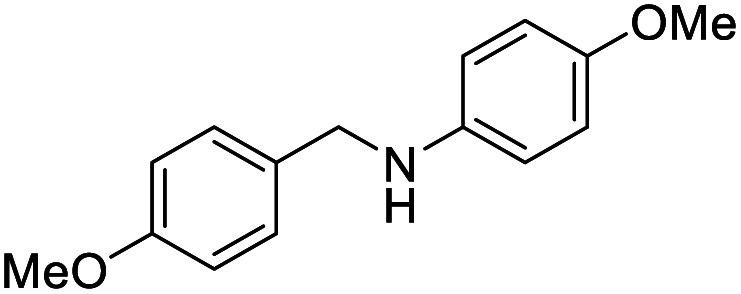	87
2	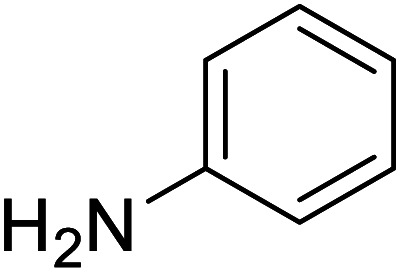	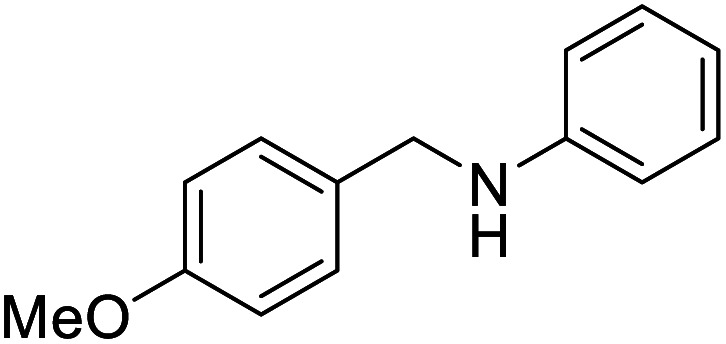	83
3	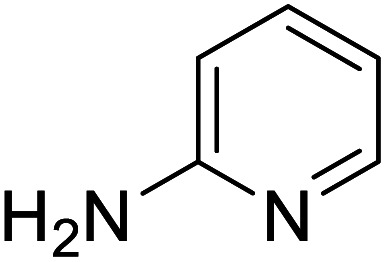	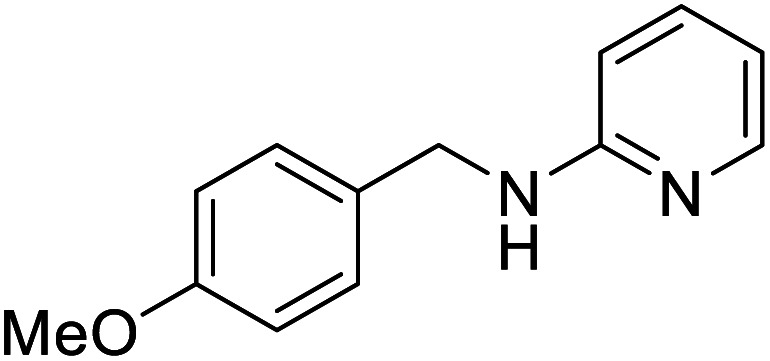	92

a Closed tube containing 1 mL of toluene and an oil bath temperature of 140 °C.

b Isolated yield.

## Conclusions

New tridentate diphosphine-phosphonite and tetradentate tripodal triphosphine–phosphite ligands were conveniently synthesized in high yields. The flexibility of ligand framework was shown through the synthesis and structural characterizations of four-, five-, mono- and dicationic Ni(ii) complexes. Their metric parameters are different from the nickel complexes containing PhP(CH_2_CH_2_PPh_2_)_2_ or PhP(CH_2_CH_2_PPh_2_)_3_ which are attributed to the presence of oxygen atom in the structure of ligand. Both the five coordinate isonitrile and chloride complexes catalyze *N*-alkylation of aromatic amines in the presence of considerably low amount (2.5 mol%) of the precatalyst which is comparable to 1 mol% of [NiCl_2_(PPh_3_)_2_] used as precatalyst in *N*-alkylation reactions.^30*k*^ The catalytic performance of the four coordinate cationic complex 4 is better and interestingly, *N*-alkylation catalysis proceeds even for the sterically encumbered and heterocyclic amines, and with an aliphatic and 4-methoxybenzyl alcohols. Other metal complexes of these ligands and catalytic studies are underway in our laboratory.

## Experimental section

### General procedure

All reactions were carried out under a nitrogen atmosphere using standard Schlenk line techniques or nitrogen-filled glove box. Petroleum ether (bp 40–60 °C) and other solvents were distilled under N_2_ atmosphere according to the standard procedures. Other chemicals were obtained from commercial sources and used as received. Ph_2_PCH_2_OH,^49^ and [NiCl_2_(DME)]^50^ were prepared according to the reported procedures. ^1^H, ^13^C, ^31^P, and ^19^F NMR spectra were recorded at room temperature. ^1^H NMR chemical shifts are referenced with respect to the chemical shift of the residual proton present in the deuterated solvent. H_3_PO_4_ (85%) was used as an external standard for ^31^P{^1^H} NMR measurements. ^19^F NMR spectra were recorded on a 400 or 500 MHz spectrometers operating at 376.5 or 470.6 MHz, respectively, for which 0.05% trifluorotoluene in CDCl_3_ was used as an external reference resonating at −62.71 ppm. Chemical shifts are in parts per million, and coupling constants are in Hz. FTIR and ATR spectra were recorded using PerkinElmer Spectrum Rx. High resolution mass spectra (ESI+/−) were obtained using Agilent Advance Bio 6545XT LC/Q-TOF system. Elemental analyses were carried out using a PerkinElmer 2400 CHN analyzer.

### Synthesis of PhP(OCH_2_PPh_2_)_2_, 1

To a solution of (diphenylphosphino)methanol (0.701 g, 3.242 mmol) in THF (15 mL) was added triethylamine (0.70 mL, 5.02 mmol) at room temperature. The solution was stirred for five minutes and then neat PhPCl_2_ (0.20 mL, 1.47 mmol) was added, resulting in the formation of colorless precipitate of triethylamine hydrochloride. After 0.5 h of stirring, the reaction mixture was filtered by using a frit. All volatiles were removed under vacuum to give compound 1 as a colourless viscous oil (0.765 g, 1.421 mmol, 97%). ^1^H NMR (CDCl_3_, 400 MHz): *δ* = 4.23 (q, 2H, *J*(H,P) = 6.1, C*H*_2_), 4.42 (q, 2H, *J*(H,P) = 6.3, C*H*_2_), 7.27–7.45 (m, 25H, C_6_*H*_5_). ^13^C NMR (CDCl_3_, 125.7 MHz): 66.1 (q, *J*(C,P) = 6.3, *C*H_2_), 128.3 (d, *J*(C,P) = 5.0), 128.5 (d, *J*(C,P) = 6.3), 128.8 (d, *J*(C,P) = 6.3), 129.0 (d, *J*(C,P) = 11.3), 130.0, 130.1 (d, *J*(C,P) = 3.8), 130.7 (d, *J*(C,P) = 10.1), 133.3 (d, *J*(C,P) = 17.6), 133.5 (d, *J*(C,P) = 18.9), 135.9 (t, *J*(C,P) = 10.7). ^31^P{^1^H} NMR (CDCl_3_, 161.9 MHz): *δ* = −13.4 (s,*P*Ph_2_), 162.9 (s, *P*Ph). HRMS (+ESI): calcd *m*/*z* for [M + H^+^]^+^: C_32_H_30_O_2_P_3_: 539.1459, found: 539.1466.

### Synthesis of P(OCH_2_PPh_2_)_3_, 2

To a solution of Ph_2_PCH_2_OH (0.715 g, 3.307 mmol) in THF (20 mL) was added triethylamine (0.50 mL, 3.59 mmol) followed by dropwise addition of PCl_3_ (0.10 mL, 1.15 mmol) at room temperature; triethylamine hydrochloride immediately began to precipitate. The whole suspension was stirring for 1 h and filtered by using a frit. All volatiles were evaporated under vacuum with warm water to give compound 2 as a colourless viscous oil (0.631 g, 0.933 mmol, 81%). ^1^H NMR (CDCl_3_, 400 MHz): *δ* = 4.29 (q, 6H, *J*(H,P) = 3.8, C*H*_2_), 7.33 (brs, 18 H, C_6_*H*_5_), 7.44 (br s, 12H, C_6_*H*_5_). ^13^C NMR (CDCl_3_, 125.7 MHz): 61.8 (q, *J*(C,P) = 6.3, *C*H_2_), 62.7, 62.8, 128.5 (d, *J*(C,P) = 7.5), 128.8 (d, *J*(C,P) = 7.5), 129.0, 129.1, 130.7 (d, *J*(C,P) = 10.1), 133.2, 133.3, 133.3, 133.4, 135.8 (d, *J*(C,P) = 11.3). ^31^P{^1^H} NMR (CDCl_3_, 161.9 MHz): *δ* = −13.8 (s, *P*Ph_2_), 140.1 (s, *P*). HRMS (+ESI): calcd *m*/*z* for [M + H^+^]^+^: 677.1688, found: 677.1693.

### Synthesis of [NiCl_2_{PhP(OCH_2_PPh_2_)_2_-κ^3^*P*,*P*,*P*}], 3

To a solution of 1 (0.201 g, 0.373 mmol) in dichloromethane (15 mL) was added [NiCl_2_(DME)] (0.083 g, 0.378 mmol) at room temperature. The solution was stirred for 12 h and the colour of solution turned to dark green. The solvent was removed under vacuum to give the greenish brown residue, which was washed with petroleum ether (2 × 10 mL) and dissolved back in dichloromethane (10 mL). Upon layering with petroleum ether (30 mL) at room temperature, dark green solid of complex 3 was formed at the junction of the layered solvents. The solid was isolated and dried under vacuum (0.188 g, 0.281 mmol, 75%). Complex was crystallized from a solution in CH_2_Cl_2_/petroleum ether upon slow evaporation at room temperature. ^1^H NMR (CDCl_3_, 400 MHz): *δ* = 4.98 (dd, ^2^*J*(H,H) = 10.8, ^2^*J*(H,P) = 29.2, 2H, C*H*_2_), 5.76 (br s, 2H, C*H*_2_), 6.93–7.88 (m, 25H, C_6_*H*_5_). ^13^C NMR (CDCl_3_, 100.6 MHz): *δ* = 72.8 (m, C*H*_2_), 127.9 (d, *J*(C,P) = 13.1), 128.8 (t, *J*(C,P) = 4.5), 129.1, 130.2 (d, *J*(C,P) = 14.1), 131.4, 131.9 (d, *J*(C,P) = 2.0), 133.2 (t, *J*(C,P) = 5.5), 134.1 (t, *J*(C,P) = 4.0). ^31^P{^1^H} NMR (CDCl_3_, 161.9 MHz): *δ* = 50.9 (d, *J*(P,P) = 39, *P*Ph_2_), 209.8 (s, *P*Ph). HRMS (+ESI): calcd *m*/*z* for [M − Cl^−^]^+^: C_32_H_29_ClNiO_2_P_3_: 631.0422, found: 631.0391. Anal. calcd for C_32_H_29_Cl_2_NiO_2_P_3_: C, 57.53; H, 4.38. Found: C, 57.14; H, 4.33.

### Synthesis of [NiCl{PhP(OCH_2_PPh_2_)_2_-κ^3^*P*,*P*,*P*}][PF_6_], 4

To a solution of 1 (0.101 g, 0.188 mmol) in dichloromethane (15 mL) was added [NiCl_2_(DME)] (0.041 g, 0.187 mmol) followed by NH_4_PF_6_ (0.040 g, 0.245 mmol). The solution was stirred at room temperature for 12 h resulting in red color solution, which was filtered and layered with petroleum ether (30 mL) at room temperature to give complex 4 as orange-red crystals formed over a period of one week. Crystals were isolated, and dried under vacuum (0.122 g, 0.157 mmol, 84%). ^1^H NMR (CDCl_3_, 400 MHz): *δ* = 5.12 (m, 1H, C*H*_2_), 5.15 (m, 1H, C*H*_2_), 5.21 (m, 1H, C*H*_2_), 5.24 (m, 1H, C*H*_2_), 5.87–5.91 (m, 2H, C*H*_2_), 6.85–7.90 (m, 25H, C_6_*H*_5_). ^31^P{^1^H} NMR (CH_2_Cl_2_, 161.9 MHz, external D_2_O locking): *δ* = −143.9 (septet, ^1^*J*(P,F) = 712.4, *P*F_6_), 59.9 (d, *J*(P,P) = 64.8, *P*Ph_2_), 218.8 (s, *P*Ph). ^19^F{^1^H} NMR (CH_2_Cl_2_, 376.5 MHz, external D_2_O locking): *δ* = −72.6 (d, ^1^*J*(P,F) = 711.6, P*F*_6_). HRMS (+ESI): calcd *m*/*z* for [M − PF_6_^−^]^+^: C_32_H_29_ClNiO_2_P_3_: 631.0417, found: 631.0443. Anal. calcd for C_32_H_29_ClF_6_NiO_2_P_4_: C, 49.43; H, 3.76. Found: C, 49.82; H, 3.82.

### Synthesis of [NiCl{PhP(OCH_2_PPh_2_)_2_-κ^3^*P*,*P*,*P*}(XylylNC)][PF_6_], 5

To a solution of ligand 1 (0.201 g, 0.373 mmol) in dichloromethane (20 mL) was added [NiCl_2_(DME)] (0.083 g, 0.378 mmol). The solution was stirred at room temperature for 10 minutes and the colour of solution is changed to greenish brown. This is followed by the successive addition of 2,6-dimethylphenyl isocyanide (0.048 g, 0.366 mmol) and KPF_6_ (0.075 g, 0.407 mmol). The solution immediately turned to a red color and the stirring was continued for additional 12 h. The solution was filtered and layered with petroleum ether (30 mL) to give red crystals of 5 over a period of one week. Crystals were separated and dried under vacuum (0.287 g, 0.316 mmol, 84%). ^1^H NMR (CDCl_3_, 400 MHz): *δ* = 1.68 (s, 6H, C*H*_3_), 5.23 (dd, ^2^*J*(H,H) = 12.2, ^2^*J*(H,P) = 36.4, 2 H, C*H*_2_), 5.42 (br s, 2 H, C*H*_2_), 6.88–7.71 (m, 25H, C_6_*H*_5_), 8.20 (br s, 3 H, C_6_*H*_5_). ^13^C NMR (CDCl_3_, 125.7 MHz): 18.2, 73.6 (m, *C*H_2_), 128.1, 128.3 (d, *J*(C,P) = 12.6), 129.8 (t, *J*(C,P) = 5.0), 130.1, 130.3, 131.5 (d, *J*(C,P) = 6.3), 132.0 (d, *J*(C,P) = 12.6), 132.3, 133.2, 133.8 (d, *J*(C,P) = 1.3), 134.7, 135.6. ^31^P{^1^H} NMR (CDCl_3_, 202.4 MHz): *δ* = −143.4 (septet, ^1^*J*(P,F) = 698.3, *P*F_6_), 62.1 (d, *J*(P,P) = 49, *P*Ph_2_), 221.9 (s, *P*Ph). ^19^F{^1^H} NMR (CDCl_3_, 470.6 MHz): *δ* = −72.7 (d, ^1^*J*(P,F) = 715.3, P*F*_6_). HRMS (+ESI): calcd *m*/*z* for [M − PF_6_^−^]^+^: C_41_H_38_ClNNiO_2_P_3_: 762.1152, found: 762.1385. Anal. calcd for C_41_H_38_ClF_6_NNiO_2_P_4_: C, 54.19; H, 4.21; N, 1.54. Found: C, 53.81; H, 3.86; N, 1.13.

### Synthesis of [Ni{PhP(OCH_2_PPh_2_)_2_-κ^3^*P*,*P*,*P*}(^*t*^BuNC)_2_][PF_6_]_2_, 6

The above procedure was followed with ligand 1 (0.201 g, 0.373 mmol), [NiCl_2_(DME)] (0.083 g, 0.378 mmol), ^*t*^butylisocyanide (0.084 mL, 0.743 mmol) and KPF_6_ (0.150 g, 0.815 mmol). Upon layering with petroleum ether, orange-red crystals of 6 were formed which were isolated and dried under vacuum (0.307 g, 0.270 mmol, 71%). ^1^H NMR (DMSO-*d*_6_, 400 MHz): *δ* = 0.87 (s, 9H, (C*H*_3_)_3_), 0.88 (s, 9H, C(C*H*_3_)_3_), 5.76 (s, 2H, C*H*_2_Cl_2_), 5.86 (d, *J*(H,H) = 8.4, 2H, C*H*_2_), 6.25 (br d, 1H, *J*(H,H) = 11.6, C*H*_2_), 6.38 (br d, *J*(H,H) = 12, 1 H, C*H*_2_), 7.06 (m, 2H, C_6_*H*_5_), 7.44–7.91 (m, 23H, C_6_*H*_5_). ^13^C NMR (DMSO-*d*_6_, 125.7 MHz): *δ* = 28.1, 28.3, 54.8 (*C*H_2_Cl_2_), 58.5, 61.2, 71.9 (t, *J*(C,P) = 15), 126.4 (t, *J*(C,P) = 21), 129.5 (d, *J*(C,P) = 14), 129.8 (t, *J*(C,P) = 5), 129.9 (d, *J*(C,P) = 13), 130.2 (t, *J*(C,P) = 4), 132.2 (t, *J*(C,P) = 5), 132.3 (t, *J*(C,P) = 5), 133.1, 133.3, 134.9. ^31^P{^1^H} NMR (DMSO-*d*_6_, 202.4 MHz): *δ* = −143.4 (septet, ^1^*J*(P,F) = 710.4, *P*F_6_), 76.7 (d, *J*(P,P) = 30.4, *P*Ph_2_), 218.4 (t, *J*(P,P) = 43.6, *P*Ph). ^19^F{^1^H} NMR (CDCl_3_, 470.6 MHz): *δ* = −71.8 (d, ^1^*J*(P,F) = 715.3, P*F*_6_). HRMS (+ESI): calcd *m*/*z* for [M − 2PF_6_^−^ + H^+^]^+^: C_42_H_48_N_2_NiO_2_P_3_: 763.2277, found: 763.2211. Anal. calcd for C_42_H_47_F_12_N_2_NiO_2_P_5_: C, 47.89; H, 4.50; N, 2.66. Found: C, 48.24; H, 4.76; N, 2.91.

### Synthesis of [Ni{PhP(OCH_2_PPh_2_)_2_-κ^3^*P*,*P*,*P*}(^i^PrNC)_2_][PF_6_]_2_, 7

The above procedure was followed with ligand 1 (0.201 g, 0.373 mmol), [NiCl_2_(DME)] (0.083 g, 0.378 mmol), isopropyl isocyanide (0.070 mL, 0.742 mmol) and KPF_6_ (0.150 g, 0.815 mmol). Upon layering with petroleum ether, orange-red crystals of 7 were formed which were isolated and dried under vacuum (0.281 g, 0.253 mmol, 67%). ^1^H NMR (CDCl_3_, 400 MHz): *δ* = 0.517 (d, *J*(H,H) = 6.8, 3H, C*H*_3_), 0.691 (d, *J*(H,H) = 6.8, 3H, C*H*_3_), 3.884 (m, 2H, C*H*), 5.629 (m, 4H, C*H*_2_), 6.971–7.862 (m, 25H, C_6_*H*_5_). ^13^C NMR (CDCl_3_, 125.7 MHz): *δ* = 20.4, 20.6, 48.2, 50.5, 71.1 (m, *C*H_2_), 125.3 (t, *J*(C,P) = 23.3), 128.4 (d, *J*(C,P) = 13,8), 129.3 (t, *J*(C,P) = 5.6), 129.5 (d, *J*(C,P) = 11.3), 129.7 (t, *J*(C,P) = 5.0), 131.3 (t, *J*(C,P) = 5.6), 131.8 (t, *J*(C,P) = 6.3), 132.2, 132.7, 133.6. ^31^P{^1^H} NMR (CDCl_3_, 202.4 MHz): *δ* = −143.5 (septet, ^1^*J*(P,F) = 713.0, *P*F_6_), 75.7 (d, *J*(P,P) = 38.5, *P*Ph_2_), 217.7 (s, *P*Ph). ^19^F{^1^H} NMR (CDCl_3_, 470.6 MHz): *δ* = −72.5 (d, ^1^*J*(P,F) = 710.6, P*F*_6_). HRMS (+ESI): calcd *m*/*z* for [M − 2PF_6_^−^+H^+^]^+^: C_40_H_44_N_2_NiO_2_P_3_: 735.1964, found: 735.1883. Anal. calcd for C_40_H_43_F_12_N_2_NiO_2_P_5_: C, 46.86; H, 4.23; N, 2.73. Found: C, 47.32; H, 4.58; N, 2.49.

### Synthesis of [NiCl{P(OCH_2_PPh_2_)_3_-κ^4^*P*,*P*,*P*,*P*}][PF_6_], 8

To a solution of 2 (0.101 g, 0.149 mmol) in dichloromethane (10 mL) was added [NiCl_2_(DME)] (0.039 g, 0.177 mmol) followed by KPF_6_ (0.043 g, 0.234 mmol). The solution was stirred at room temperature for 6 h resulting in a deep purple color solution, which was filtered and layered with petroleum ether (30 mL) at room temperature to give complex 8 as purple crystals formed over a period of one week. Crystals were isolated, and dried under vacuum (0.073 g, 0.080 mmol, 45%). ^1^H NMR (DMSO-*d*_6_, 500 MHz): *δ* = 5.51. (d, *J*(H,H) = 22.5, 6H, C*H*_2_), 7.12–7.35 (m, 30H, C_6_*H*_5_). ^13^C NMR (DMSO-*d*_6_, 125.7 MHz): 78.9 (t, *J*(C,P) = 33.2, *C*H_2_), 128.8 (br m), 130.1 (d, *J*(C,P) = 9.5), 131.0, 131.4 (br m). ^31^P{^1^H}NMR (DMSO-*d*_6_, 202.4 MHz): *δ* = −143.5 (quintet, ^1^*J*(P,F) = 711.4, *P*F_6_), 46.3 (d, *J*(P,P) = 53.0, *P*Ph_2_), 204.0 (m, *P*). ^19^F{^1^H}NMR (DMSO-*d*_6_, 470.6 MHz): *δ* = −71.8 (d, ^1^*J*(P,F) = 712.0, P*F*_6_). HRMS (+ESI): calcd *m*/*z* for [M − PF_6_^−^]^+^: C_39_H_36_ClNiO_3_P_4_: 769.0651, found: 769.0638.

### General procedure for *N*-alkylation of amine reactions

Amines (0.50 mmol), alcohols (1.00 mmol), complex 4 (2.50 mol% with respect to amine), KOBu^*t*^ (0.40 mmol), and toluene (1 mL) were taken in a 60 mL oven-dried pressure tube under open atmosphere, and then tube was purged with N_2_ gas by using septum and needle for five minutes. The tube was capped and the content of the tube was immersed in an oil bath maintained at 140 °C with stirring for 24 h. The progress of the product formation was monitored with thin layer chromatography. The reaction mixture was cooled to room temperature and extracted with dichloromethane (3 × 10 mL). The solvent was removed under vacuum from the combined solution and the residue was loaded onto a silica gel column. Elution using ethyl acetate/petroleum ether (v/v = 1 : 4) mixture gave the first fraction from which solvents were removed under vacuum to give the product in a pure form.

#### *N*-Benzyl-4-methoxyaniline^30*g*,51^

0.100 g, 0.469 mmol, 94%. ^1^H NMR (CDCl_3_, 500 MHz): 3.64 (s, 3H, OC*H*_3_), 4.18 (s, 2H, C*H*_2_), 6.50 (m, 2H, C_6_*H*_5_), 6.68 (m, 2H, C_6_*H*_5_), 7.13–7.23 (m, 5H). ^13^C{^1^H} NMR (CDCl_3_, 125.7 MHz): 49.4, 55.9, 114.3, 115.1, 127.3, 127.7, 128.7, 139.9, 142.6, 152.4.

#### *N*-Benzylaniline^30*g*,51^

0.084 g, 0.458 mmol, 92%. ^1^H NMR (CDCl_3_, 500 MHz): 3.92 (br s, 1H, N*H*), 4.24 (s, 2H, C*H*_2_), 6.56 (m, 2H, C_6_*H*_5_), 6.65 (t, 1H, C_6_*H*_5_), 7.07–7.29 (m, 7H). ^13^C{^1^H} NMR (CDCl_3_, 125.7 MHz): 48.5, 113.0, 117.7, 127.4, 127.6, 128.8, 129.4, 139.6, 148.3.

#### *N*-Benzyl-4-bromoaniline^30*a*,51^

0.115 g, 0.439 mmol, 88%. ^1^H NMR (CDCl_3_, 500 MHz): 3.98 (br s, 1H, N*H*), 4.21 (s, 2H, C*H*_2_), 6.42 (d, *J*(H,H) = 8.5 MHz, 2H, C_6_*H*_5_), 7.18–7.30 (m, 7H). ^13^C{^1^H} NMR (CDCl_3_, 125.7 MHz): 48.4, 109.3, 114.6, 127.51, 127.53, 128.8, 132.1, 139.1, 147.2.

#### *N*-Benzyl-2,6-diisopropylaniline^30*a*,*h*,51^

0.096 g, 0.359 mmol, 72%. ^1^H NMR (CDCl_3_, 500 MHz): 1.276 (d, *J*(H,H) = 2.5, 7H), 1.292 (s, 5H), 2.957 (m, 2H), 3.817 (br s, 1H), 6.794–8.206 (m, 8H). ^13^C{^1^H} NMR (CDCl_3_, 125.7 MHz): 22.6, 23.61, 28.11, 29.85, 118.72, 122.93, 123.17, 124.26, 128.73, 128.97, 131.55, 132.65, 137.77, 140.39, 162.15.

#### *N*-Benzylpyridin-2-amine^30*a*^

0.0838 g, 0.455 mmol, 91%. ^1^H NMR (CDCl_3_, 500 MHz): 4.41 (d, *J*(H,H) = 4.0, 2 H), 4.87 (br s, 1H), 6.28 (d, *J*(H,H) = 8.5, 1H), 6.49 (t, *J*(H,H) = 5.8, 1H), 7.17–7.30 (m, 6 H), 8.00 (d, *J*(H,H) = 3.0, 1 H). ^13^C{^1^H} NMR (CDCl_3_, 125.7 MHz): 46.5, 106.9, 113.3, 127.4, 127.5, 128.8, 137.6, 139.4, 148.3, 158.8.

#### *N*-(^*n*^Bu)-4-Methoxyaniline^30*c*^

0.034 g, 0.190 mmol, 38%. ^1^H NMR (CDCl_3_, 500 MHz): 1.26–1.607 (m, 9H), 3.068 (t, *J*(H,H) = 7, 2H), 3.707–3.913 (m, 3H), 6.572–7.923 (m, 4H). ^13^C{^1^H} NMR (CDCl_3_, 125.7 MHz): 14.06, 20.47, 29.84 (grease), 3199, 44.89, 56.02, 114.2, 115.12, 143.08, 152.18.

#### 4-Methoxy-*N*-(4-methoxybenzyl)aniline^53^

0.106 g, 0.435 mmol, 87%. ^1^H NMR (CDCl_3_, 400 MHz): 3.76 (s, 3H, OC*H*_3_), 3.81 (s, 3H, OC*H*_3_), 4.22 (s, 2H, C*H*_2_), 6.62 (d, *J*(H,H) = 8.8, 2H, C_6_*H*_5_), 6.80 (d, *J*(H,H) = 8.8, 2H, C_6_*H*_5_), 6.90 (d, *J*(H,H) = 8.4, 2H, C_6_*H*_5_), 7.30 (d, *J*(H,H) = 8.4, 2H, C_6_*H*_5_). ^13^C{^1^H} NMR (CDCl_3_, 125.7 MHz): 48.8, 55.4, 55.9, 114.1, 114.3, 115.0, 128.9, 131.8, 142.7, 152.3, 158.9.

#### *N*-(4-Methoxybenzyl)aniline^30*b*,54^

0.088 g, 0.413 mmol, 83%. ^1^H NMR (CDCl_3_, 400 MHz): 3.83 (s, 3H, OC*H*_3_), 3.914 (br s, 1H, N*H*), 4.28 (s, 2H, C*H*_2_), 6.67 (d, *J*(H,H) = 8.4, 2H, C_6_*H*_5_), 6.76 (t, *J*(H,H) = 7.2, 1H, C_6_*H*_5_), 6.92 (d, *J*(H,H) = 8.4, 2H, C_6_*H*_5_), 7.22 (t, *J*(H,H) = 7.4, 2H, C_6_*H*_5_), 7.33 (d, *J*(H,H) = 8.0, 2H, C_6_*H*_5_). ^13^C{^1^H} NMR (CDCl_3_, 125.7 MHz): 47.9, 55.4, 113.0, 114.2, 117.6, 128.9, 129.4, 131.6, 148.4, 159.0.

#### *N*-(4-Methoxybenzyl)pyridin-2-amine^30*e*,*g*,55^

0.099 g, 0.462 mmol, 92%. ^1^H NMR (CDCl_3_, 400 MHz): 3.80 (s, 3H, OC*H*_3_), 4.43 (d, *J*(H,H) = 5.6, 2H, C*H*_2_), 4.81 (s, 1H, N*H*), 6.37 (d, *J*(H,H) = 8.4, 1H, C_5_*H*_4_), 6.59 (t, *J*(H,H) = 6.0, 1H, C_5_*H*_4_), 6.87 (d, *J*(H,H) = 8.4, 2H, C_6_*H*_5_), 7.27 (t, *J*(H,H) = 7.0, 2H, C_6_*H*_5_), 7.40 (t, *J*(H,H) = 7.8, 1H, C_6_*H*_5_), 8.11 (d, *J*(H,H) = 4.8, 1H, C_5_*H*_4_). ^13^C{^1^H} NMR (CDCl_3_, 125.7 MHz): 29.8 (grease), 46.0, 55.5, 107.0, 113.3, 114.2, 128.9, 131.4, 137.6, 148.3, 158.8, 159.1.

## Conflicts of interest

There are no conflicts to declare.

## Supplementary Material

RA-012-D1RA08961G-s001

RA-012-D1RA08961G-s002

## References

[cit1] Hartwig J. F. , Organotransition Metal Chemistry, From Bonding to Catalysis, University Science Books, 2010

[cit2] Hierso J. C., Amardeil R., Bentabet E., Broussier R., Gautheron B., Meunier P., Kalck P. (2003). Coord. Chem. Rev..

[cit3] Phanopoulos A., Long N. J., Miller P. W. (2017). Struct. Bonding.

[cit4] Krytchankou I. S., Krupenya D. V., Karttunen A. J., Tunik S. P., Pakkanen T. A., Chou P. T., Koshevoy I. O. (2014). Dalton Trans..

[cit5] Ning Y., Sarjeant A. A., Stern C. L., Peterson T. H., Nguyen S. T. (2012). Inorg. Chem..

[cit6] Mukhopadhyay T. K., Flores M., Feller R. K., Scott B. L., Taylor R. D., Paz-Pasternak M., Henson N. J., Rein F. N., Smythe N. C., Trovitch R. J., Gordon J. C. (2014). Organometallics.

[cit7] Cook R. E., Phelan B. T., Shoer L. E., Majewski M. B., Wasielewski M. R. (2016). Inorg. Chem..

[cit8] Peori M. B., Kakkar A. K. (2002). Organometallics.

[cit9] Roddick D. M., Zargarian D. (2014). Inorg. Chim. Acta.

[cit10] Ananikov V. P. (2015). ACS Catal..

[cit11] Bourbigou H. O., Breuil P. A. R., Magna L., Michel T., Pastor M. F. E., Delcroix D. (2020). Chem. Rev..

[cit12] Khake S. M., Chatani N. (2019). Trends Chem..

[cit13] Zimmermann P., Limberg C. (2017). J. Am. Chem. Soc..

[cit14] Tasker S. Z., standley E. A., Jamison T. F. (2014). Nature.

[cit15] Maji M., Panja D., Borthakur I., Kundu S. (2021). Org. Chem. Front..

[cit16] Smith M. B. , March's Advanced Organic Chemistry, Wiley India Pvt. Ltd, New Delhi, 7th edn, 2013, p. 481

[cit17] Mueller T. E., Hultzsch K. C., Yus M., Foubelo F., Tada M. (2008). Chem. Rev..

[cit18] Castillo P. R., Buchwald S. L. (2016). Chem. Rev..

[cit19] Kan T., Fukuyama T. (2004). Chem. Commun..

[cit20] Buzo S. R., Concepción P., Corma A., Moliner M., Boronat M. (2021). ACS Catal..

[cit21] Dobereiner G. E., Crabtree R. H. (2010). Chem. Rev..

[cit22] Hamid M. H. S. A., Allen C. L., Lamb G. W., Maxwell A. C., Maytum H. C., Watson A. J. A., Williams J. M. J. (2009). J. Am. Chem. Soc..

[cit23] Wong C. M., Peterson M. B., Pernik I., McBurney R. T., Messerle B. A. (2017). Inorg. Chem..

[cit24] Fujita K., Li Z., Ozeki N., Yamaguchi R. (2003). Tetrahedron Lett..

[cit25] Huang R., Yang Y., Wang D. S., Zhang L., Wang D. (2018). Org. Chem. Front..

[cit26] Wei D., Yang P., Yu C., Zhao F., Wang Y., Peng Z. (2021). J. Org. Chem..

[cit27] Liu Z., Yang Z., Yu X., Zhang H., Yu B., Zhao Y., Liu Z. (2017). Adv. Synth. Catal..

[cit28] Zhao Y., Foo S. W., Saito S. (2011). Angew. Chem., Int. Ed..

[cit29] Zhang Y., Qi X., Cui X., Shi F., Deng Y. (2011). Tetrahedron Lett..

[cit30] Bains A. K., Kundu A., Yadav S., Adhikari D. (2019). ACS Catal..

[cit31] Braunstein P., Fryzuk M. D., Nauda F., Rettig S. J. (1999). J. Chem. Soc., Dalton Trans..

[cit32] Yang L., Powell D. R., Houser R. P. (2007). Dalton Trans..

[cit33] Murugesan K., Bheeter C. B., Linnebank P. R., Spannenberg A., Reek J. N., Jagadeesh R. V., Beller M. (2019). ChemSusChem.

[cit34] Bertinsson G. I. (1983). Acta Crystallogr., Sect. C: Cryst. Struct. Commun..

[cit35] Alwaaly A., Clegg W., Harrington R. W., Petrou A. L., Henderson R. A. (2015). Dalton Trans..

[cit36] Autissier V., Zarza P. M., Petrou A., Henderson R. A., Harrington R. W., Clegg W. C. (2004). Inorg. Chem..

[cit37] Clegg W., Henderson R. A. (2002). Inorg. Chem..

[cit38] Ramírez A. A., Álamo M. F., Jones W. D., García J. J. (2008). Organometallics.

[cit39] Yamamoto Y., Takahata H., Takei F. (1997). J. Organomet. Chem..

[cit40] Baxley G. T., Miller W. K., Lyon D. K., Miller B. E., Nieckarz G. F., JR Weakley T., Tyler D. R. (1996). Inorg. Chem..

[cit41] Handley D. A., Hitchcock P. B., Leigh G. J. (2001). Inorg. Chim. Acta.

[cit42] Cucciolito M. E., Felice V. D., Orabona I., Panunzi A., Ruffo F. (2003). Inorg. Chim. Acta.

[cit43] Arbuckle B. W., Musker W. K. (1991). Polyhedron.

[cit44] Hou H., Gantzel P. K., Kubiak C. P. (2003). Organometallics.

[cit45] Tanabiki M., Tsuchiya K., Kumanomido Y., Matsubara K., Motoyama Y., Nagashima H. (2004). Organometallics.

[cit46] Brennessel W. W., Kucera B. E., Young Jr V. G., Ellis J. E. (2019). Acta Crystallogr., Sect. C: Struct. Chem..

[cit47] Subramaniyan V., Kumar A., Govindaraj A., Mani G. (2019). Acta Crystallogr., Sect. C: Struct. Chem..

[cit48] Fox M. A., Chandler D. A., Kyba E. P. (1992). J. Coord. Chem..

[cit49] Hohman W. H., Kountz D. J., Meek D. W. (1986). Inorg. Chem..

[cit50] Vaira M. D., Midollini S., Sacconi L. (1977). Inorg. Chem..

[cit51] Ghilardi C. A., Mealli C., Midollini S., Orlandini A., Proserpio D. M. (1990). Struct. Chem..

[cit52] Whyte T., Casey A. T., Williams G. A. (1995). Inorg. Chem..

[cit53] Biriukov K. O., Vinogradov M. M., Afanasyev O. I., Vasilyev D. V., Tsygankov A. A., Godovikova M., Nelyubina Y. V., Loginov D. A., Chusov D. (2021). Catal. Sci. Technol..

[cit54] Zhang G., Yin Z., Zheng S. (2016). Org. Lett..

[cit55] Singh A., Maji A., Joshi M., Choudhury A. R., Ghosh K. (2021). Dalton Trans..

