# Supplementary material for: Four- and five-coordinate nickel(ii) complexes bearing new diphosphine–phosphonite and triphosphine–phosphite ligands: catalysts for N-alkylation of amines
Source: RSC Adv. 2022 Feb 3;12(8):4510–20. doi: 10.1039/d1ra08961g (PMC8981024; doi:10.1039/d1ra08961g)
Supplement: RA-012-D1RA08961G-s001 [file RA-012-D1RA08961G-s001.pdf]

Electronic Supplementary Material (ESI) for RSC Advances.
This journal is © The Royal Society of Chemistry 2021

Supporting Information

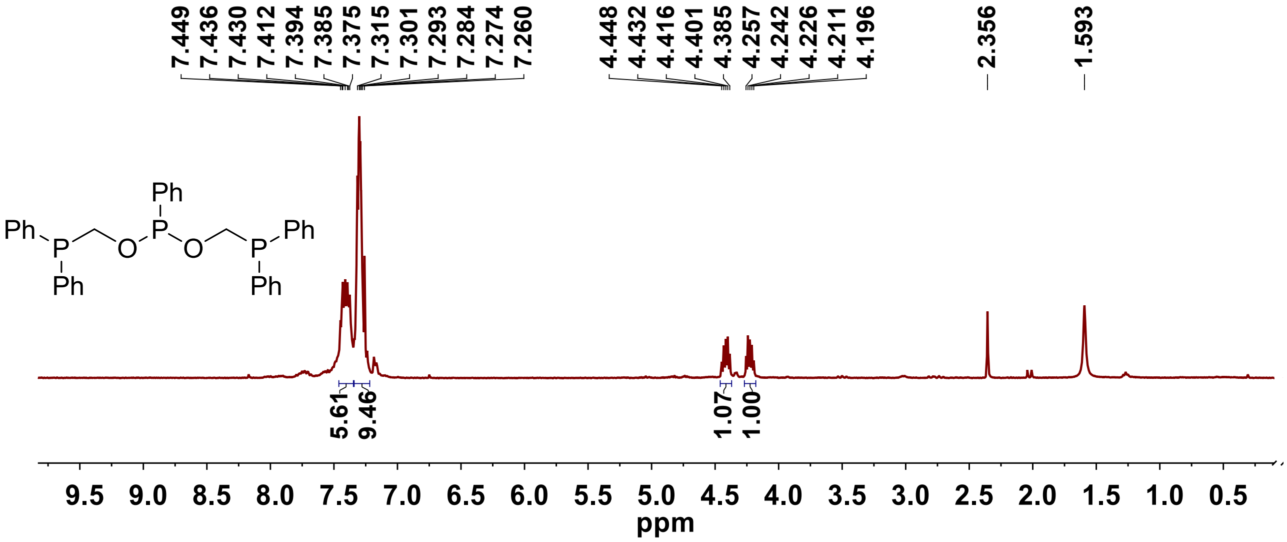

Figure S1. ^{1}H NMR (400 MHz) spectrum of $PhP(OCH_2PPh_2)_2$ **1** in $CDCl_3$. The peak at 2.36 ppm is due to the toluene methyl group as an impurity.

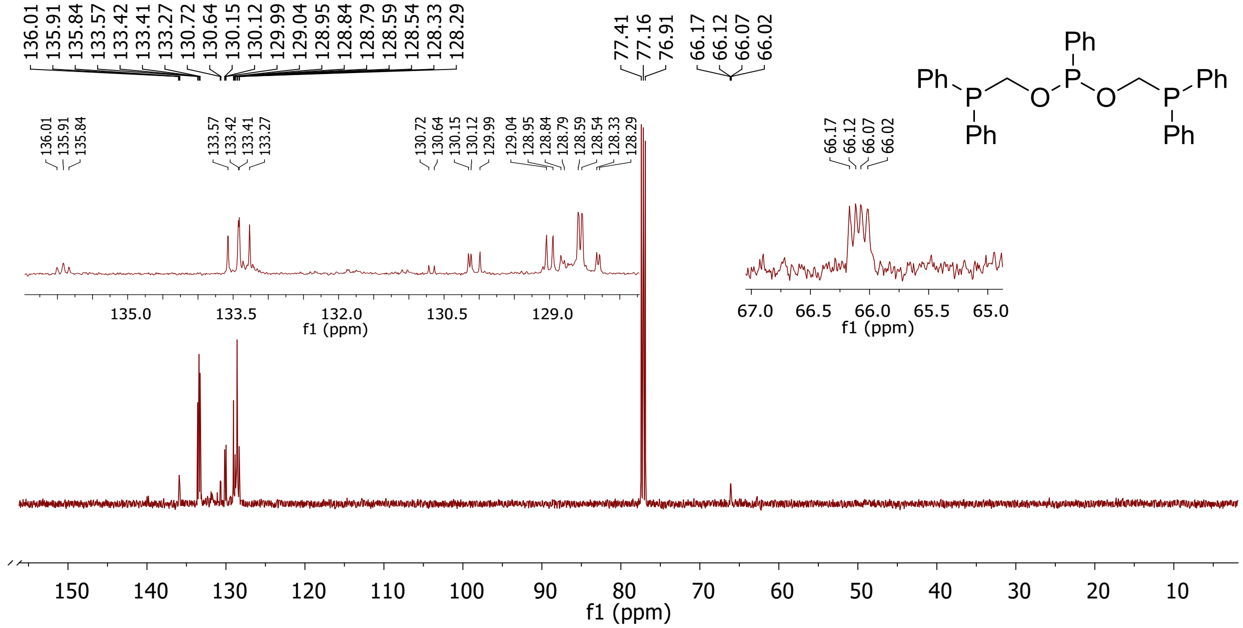

Figure S2. ^{13}C NMR (125.7 MHz) spectrum of $PhP(OCH_2PPh_2)_2$ **1** in $CDCl_3$.

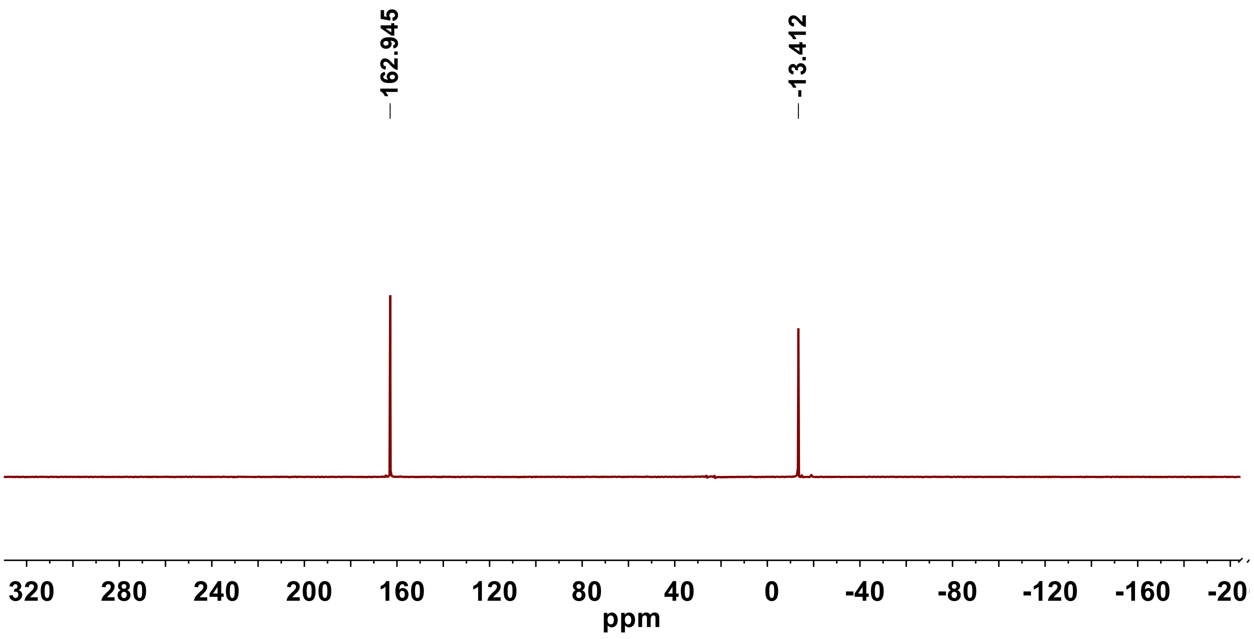

Figure S3. ^{31}P NMR (161.9 MHz) spectrum of $PhP(OCH_2PPh_2)_2$ **1** in $CDCl_3$.

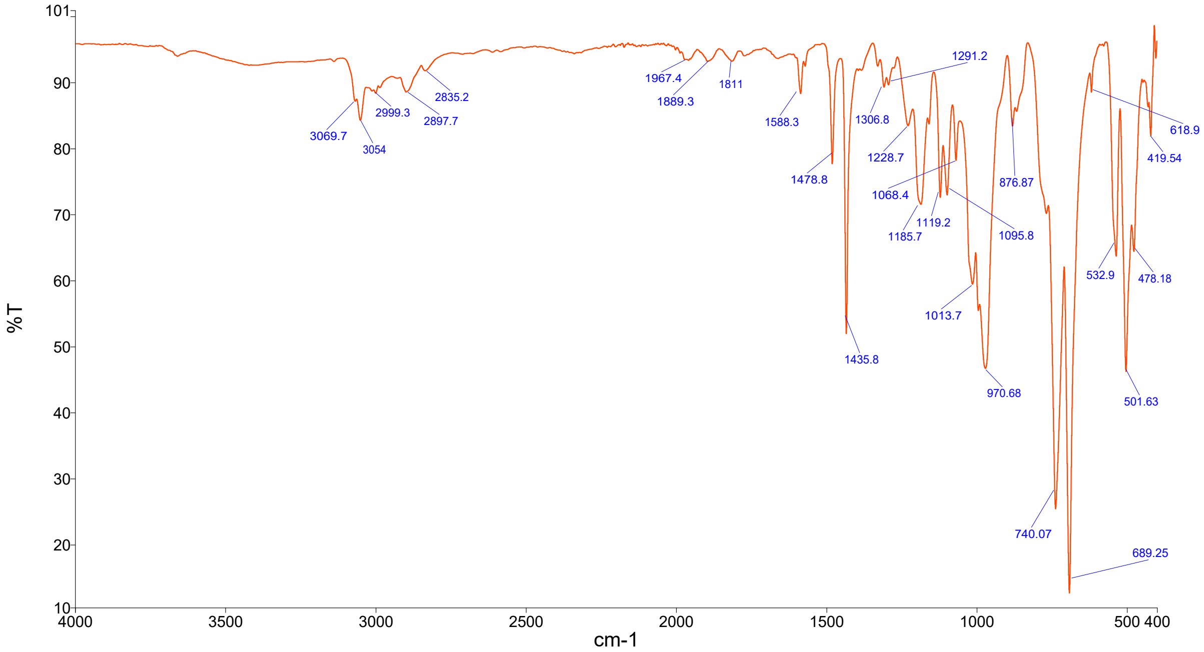

Figure S4. ATR-IR spectrum of $PhP(OCH_2PPh_2)_2$ **1**.

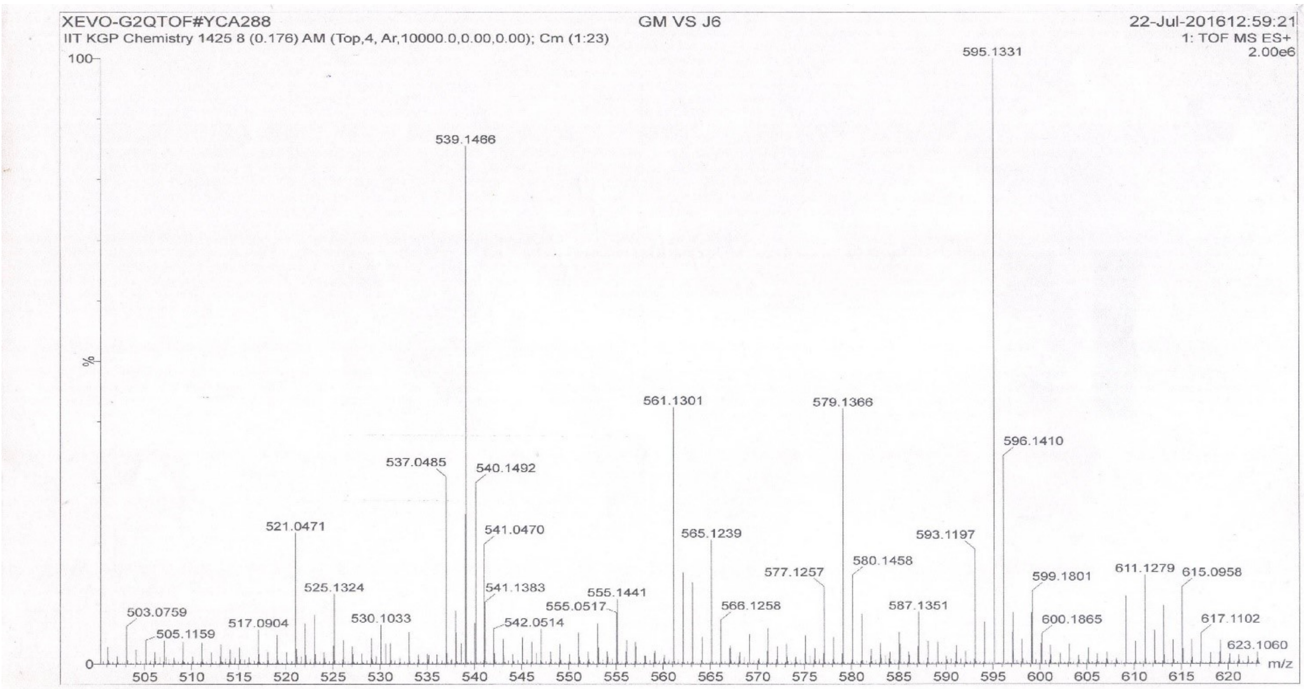

Figure S5. HRMS spectrum of $PhP(OCH_2PPh_2)_2$ **1**.

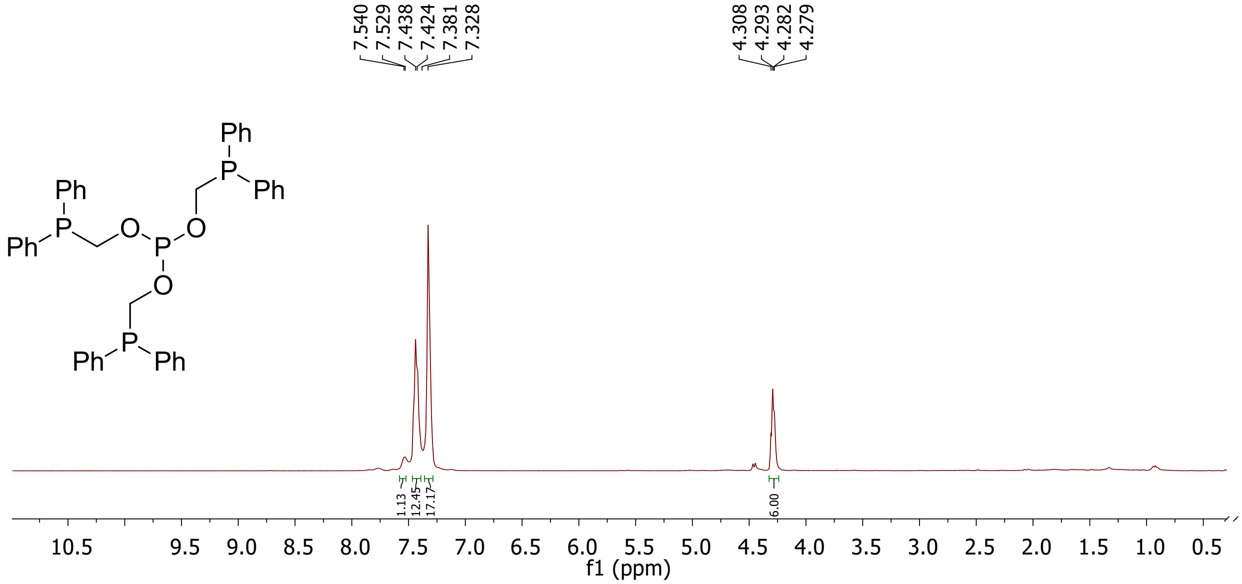

Figure S6. ^{1}H NMR (400 MHz) spectrum of $P(OCH_2PPh_2)_3$ **2** in $CDCl_3$.

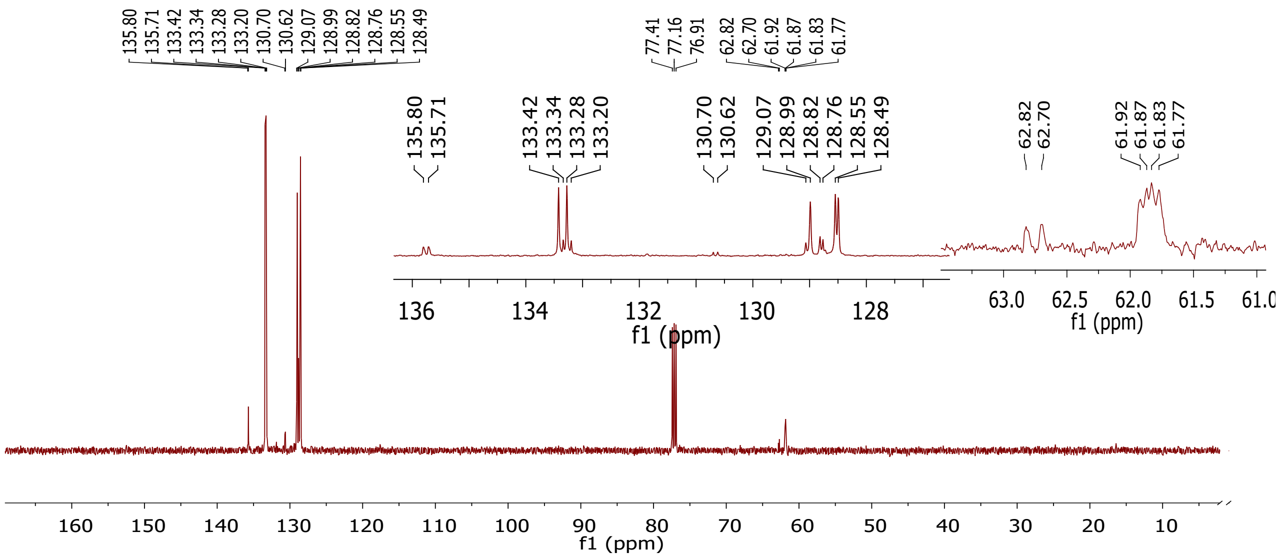

Figure S7. ^{13}C NMR (125.7 MHz) spectrum of $P(OCH_2PPh_2)_3$ **2** in $CDCl_3$.

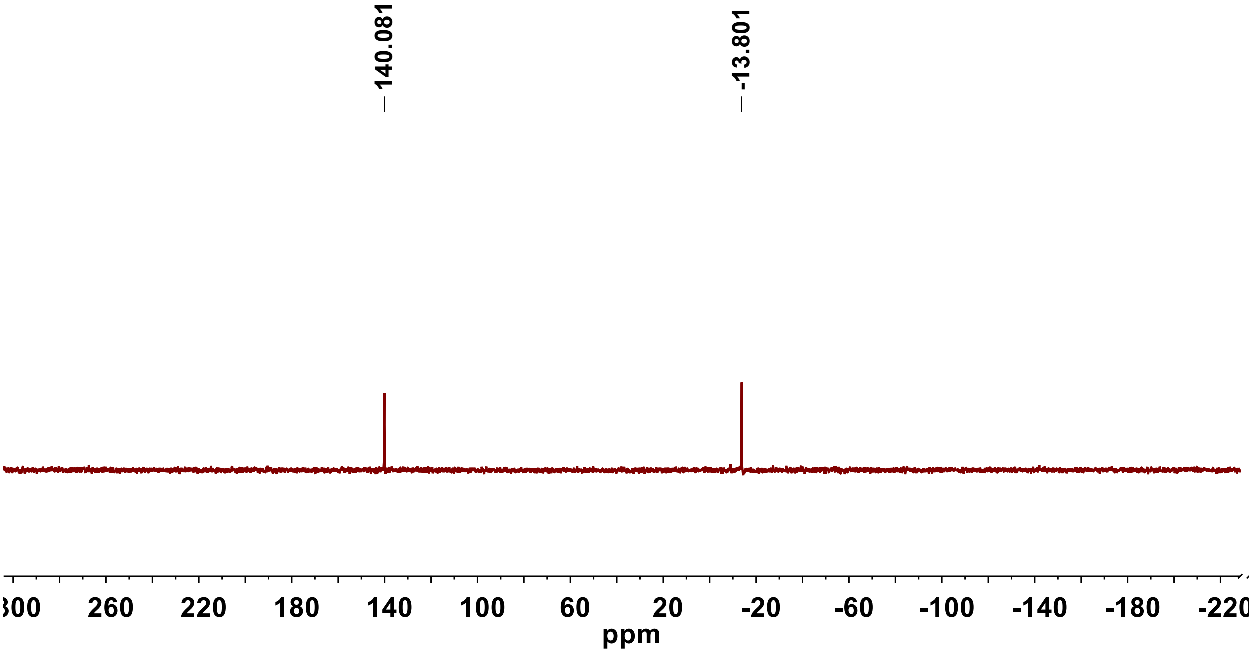

Figure S8. ^{31}P NMR (161.9 MHz) spectrum of $P(OCH_2PPh_2)_3$ **2** in $CDCl_3$.

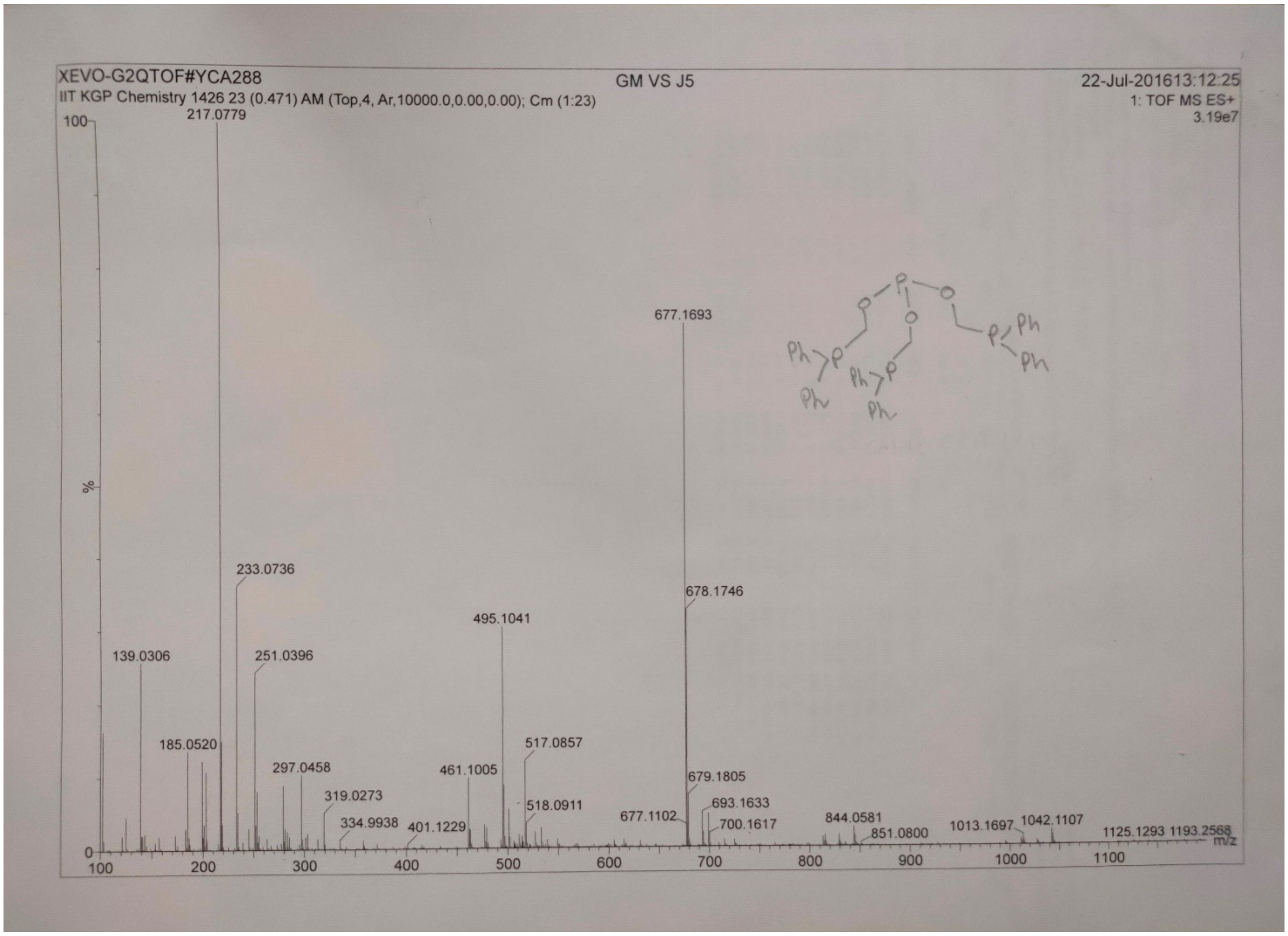

Figure S9. HRMS spectrum of $P(OCH_2PPh_2)_3$ **2**.

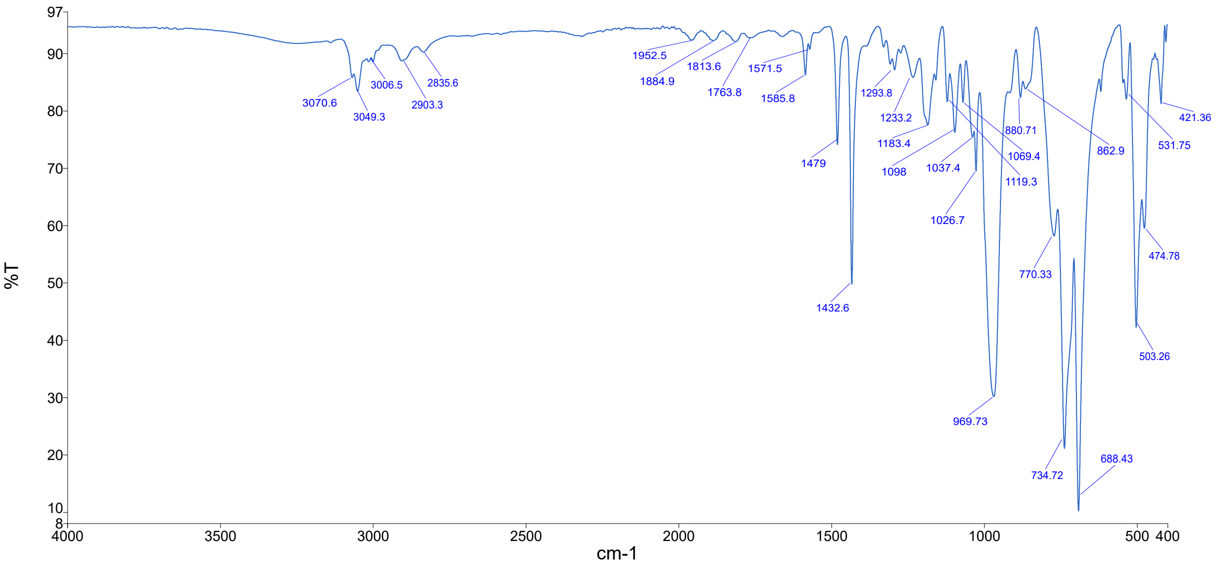

Figure S10. ATR-IR spectrum of $P(OCH_2PPh_2)_3$ **2**.

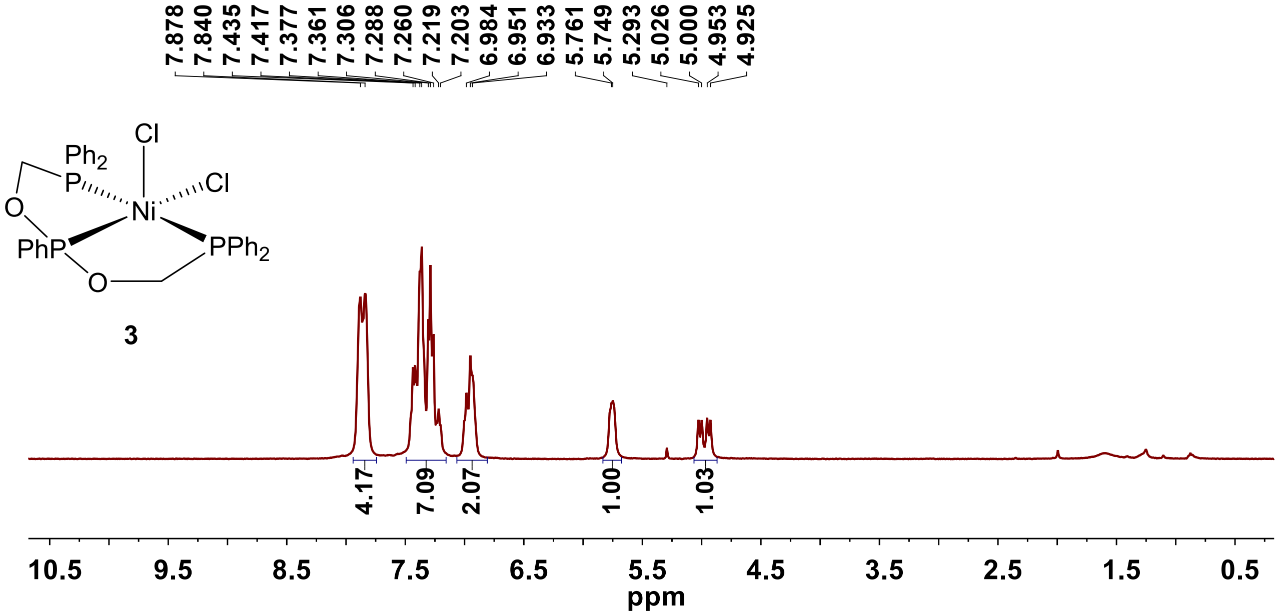

Figure S11. ^{1}H NMR (400 MHz) spectrum of [$NiCl_2${PhP(OCH_2PPh_2)$_2$-κ^3P,P,P}] **3** in $CDCl_3$.

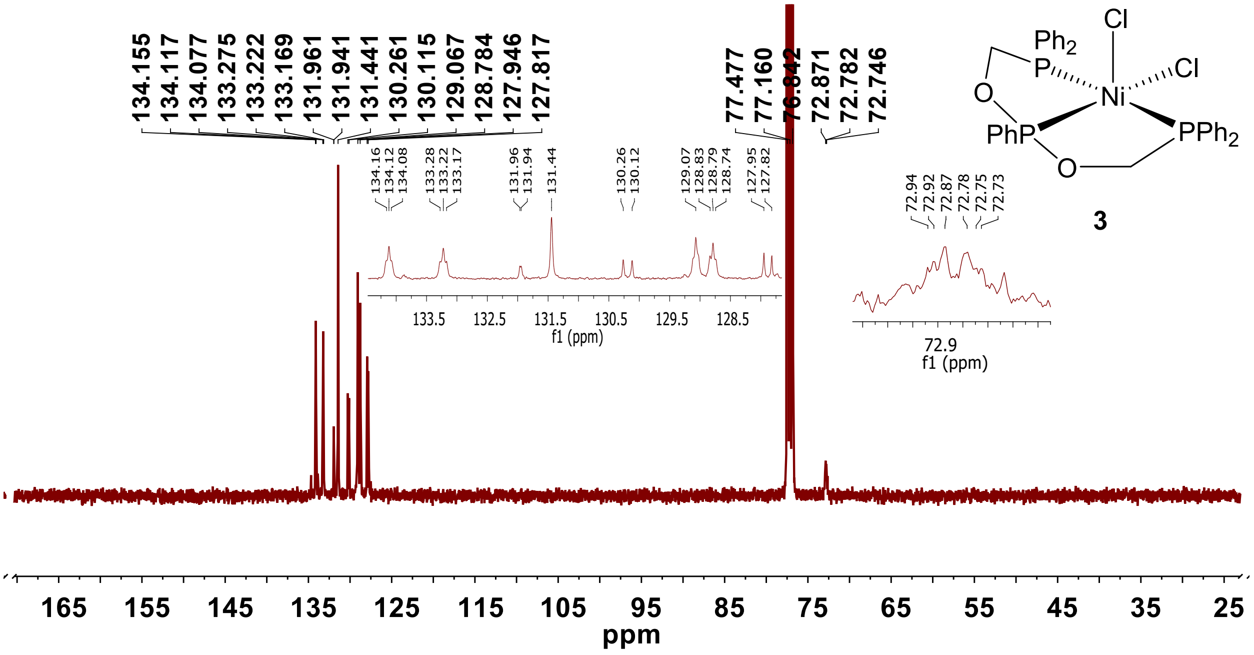

Figure S12. ^{13}C NMR (100.6 MHz) spectrum of [$NiCl_2${PhP(OCH_2PPh_2)$_2$-κ^3P,P,P}] **3** in $CDCl_3$.

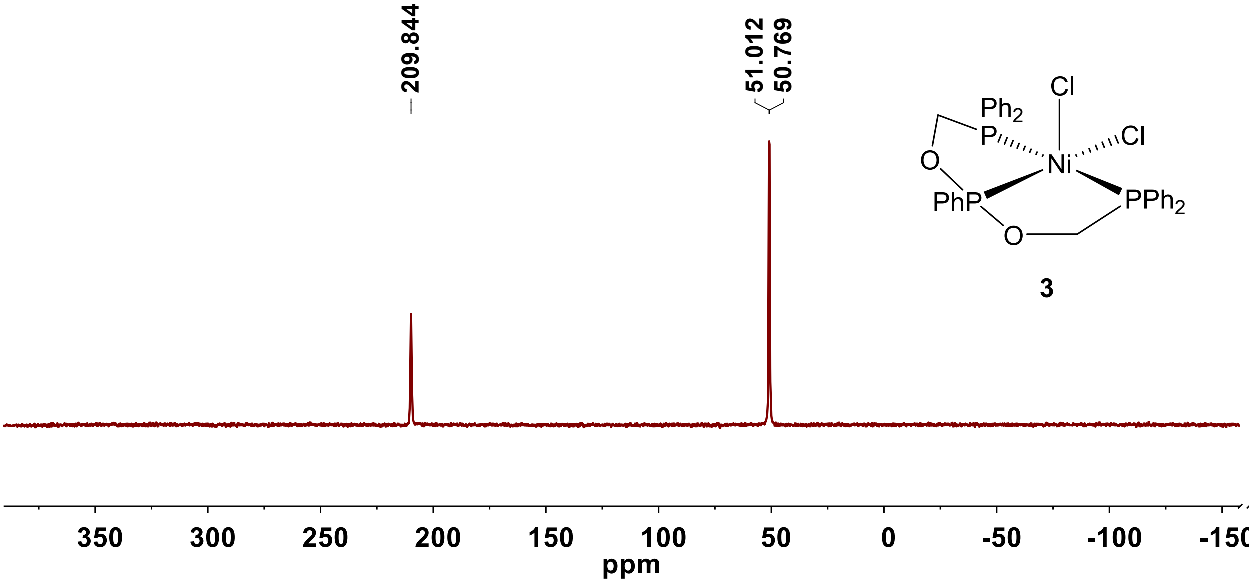

Figure S13. ^{31}P NMR (161.9 MHz) spectrum of $[NiCl_2\{PhP(OCH_2PPh_2)_2\text{-}\kappa^3 P,P,P\}]$ **3** in $CDCl_3$.

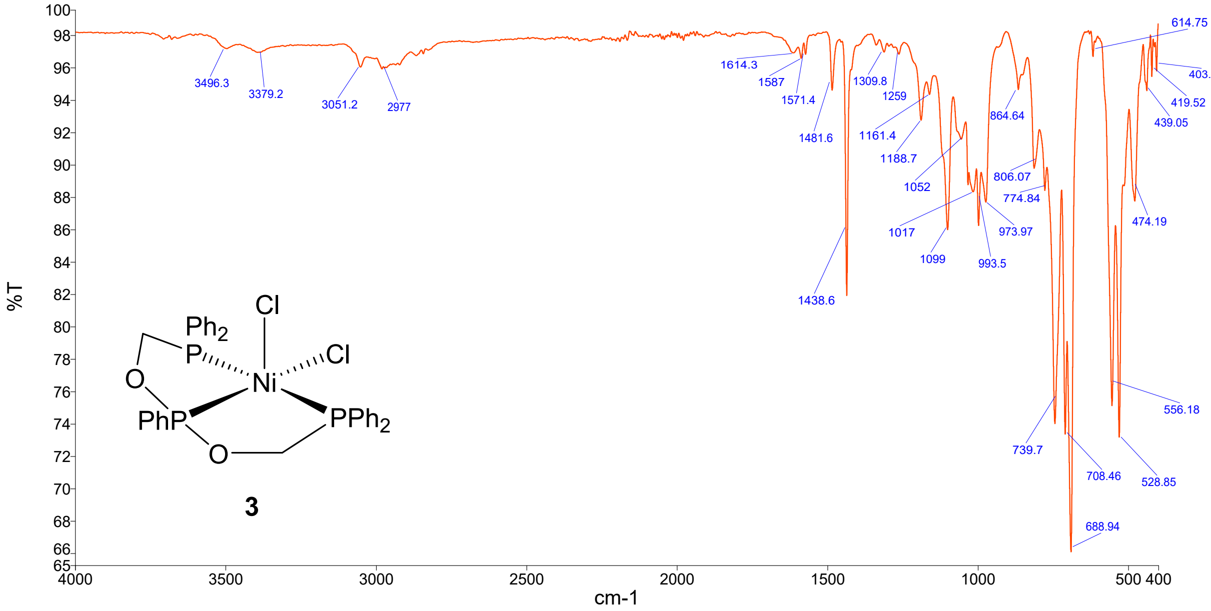

Figure S14. ATR spectrum of $[NiCl_2\{PhP(OCH_2PPh_2)_2\text{-}\kappa^3 P,P,P\}]$ **3**.

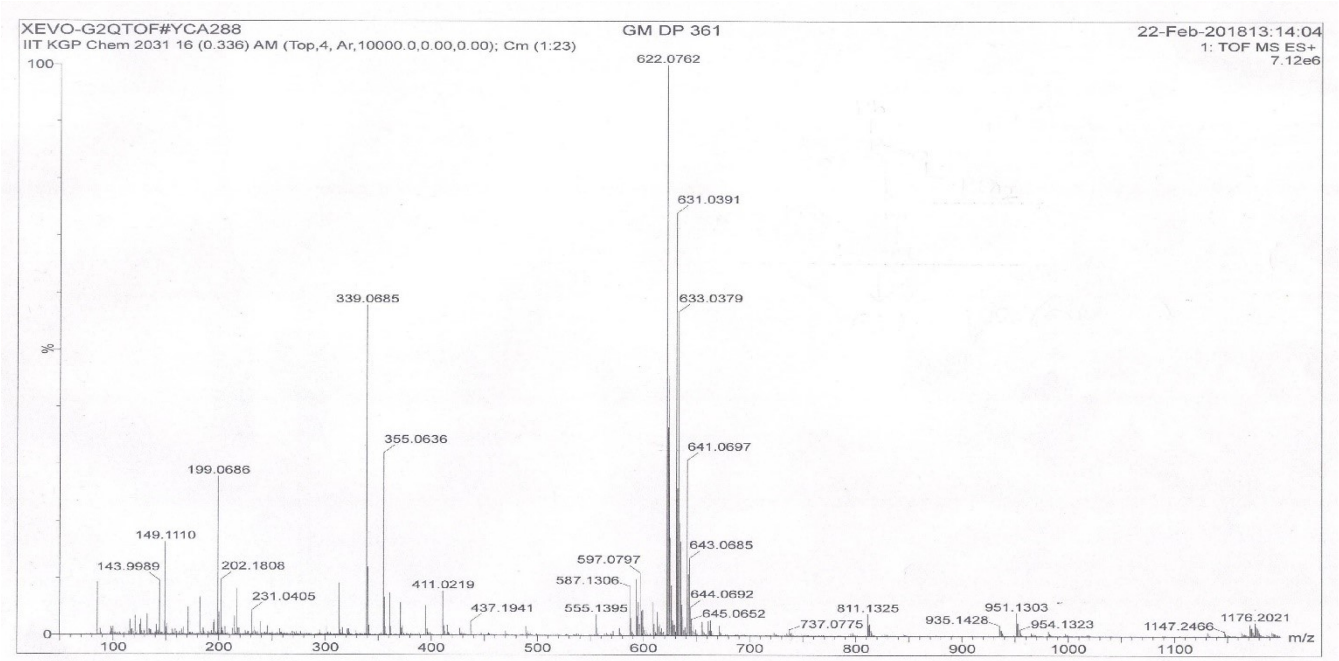

Figure S15. HRMS spectrum of [NiCl{PhP(OCH_2PPh_2)$_2$-κ^3P,P,P}][PF_6], **4**.

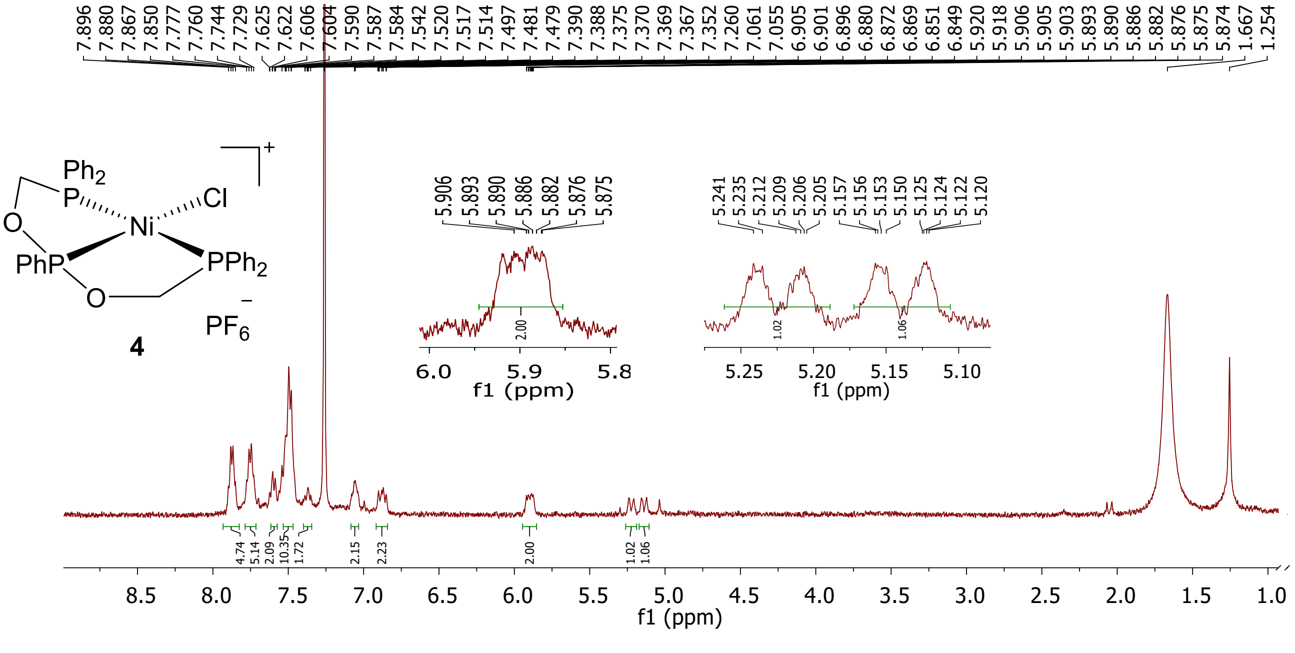

Figure S16. ^{1}H NMR (400 MHz) spectrum of [NiCl{PhP(OCH_2PPh_2)$_2$-κ^3P,P,P}][PF_6], **4** in $CDCl_3$.

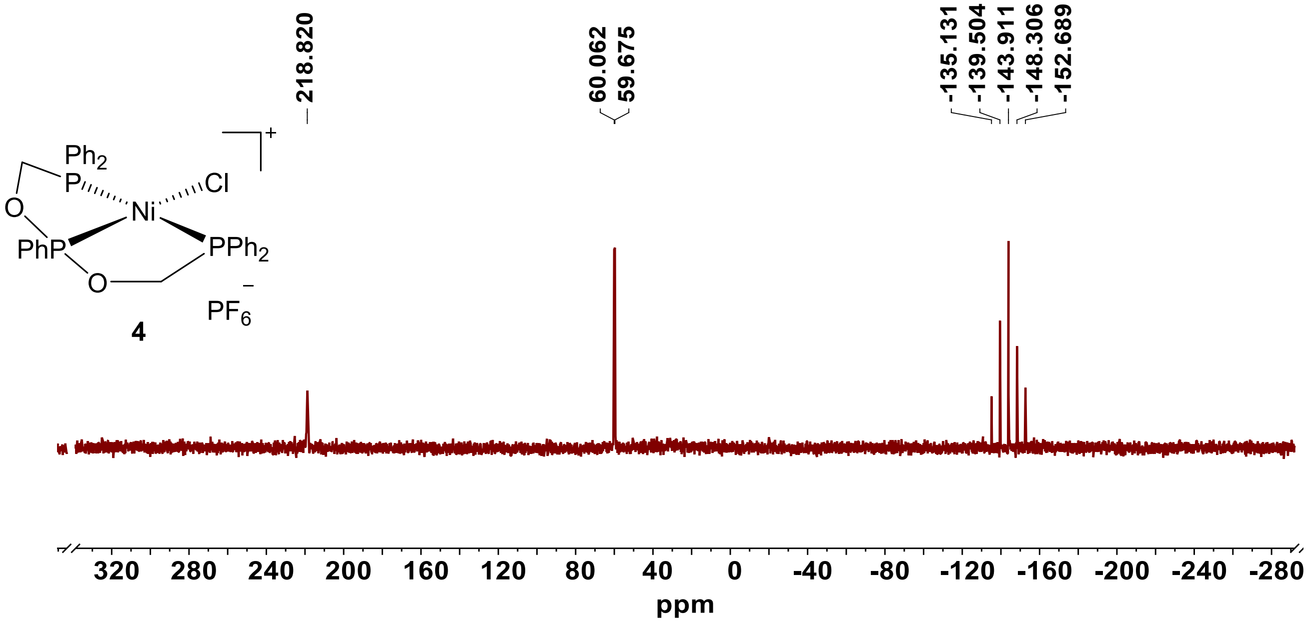

Figure S17. ^{31}P NMR (161.9 MHz) spectrum of [NiCl{PhP(OCH_2PPh_2)$_2$-κ^3*P*,*P*,*P*}][PF_6], **4** in CH_2Cl_2 with D_2O external locking.

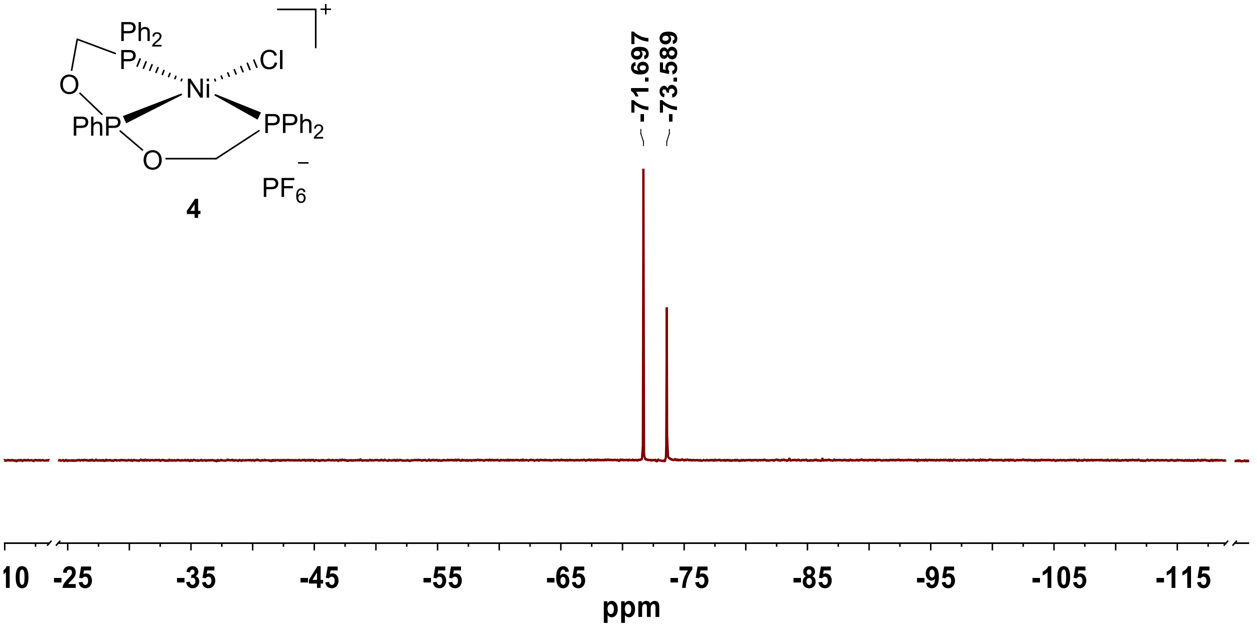

Figure S18. ^{19}F NMR (376.5 MHz) spectrum of [NiCl{PhP(OCH_2PPh_2)$_2$-κ^3*P*,*P*,*P*}][PF_6], **4** in CH_2Cl_2 with D_2O external locking.

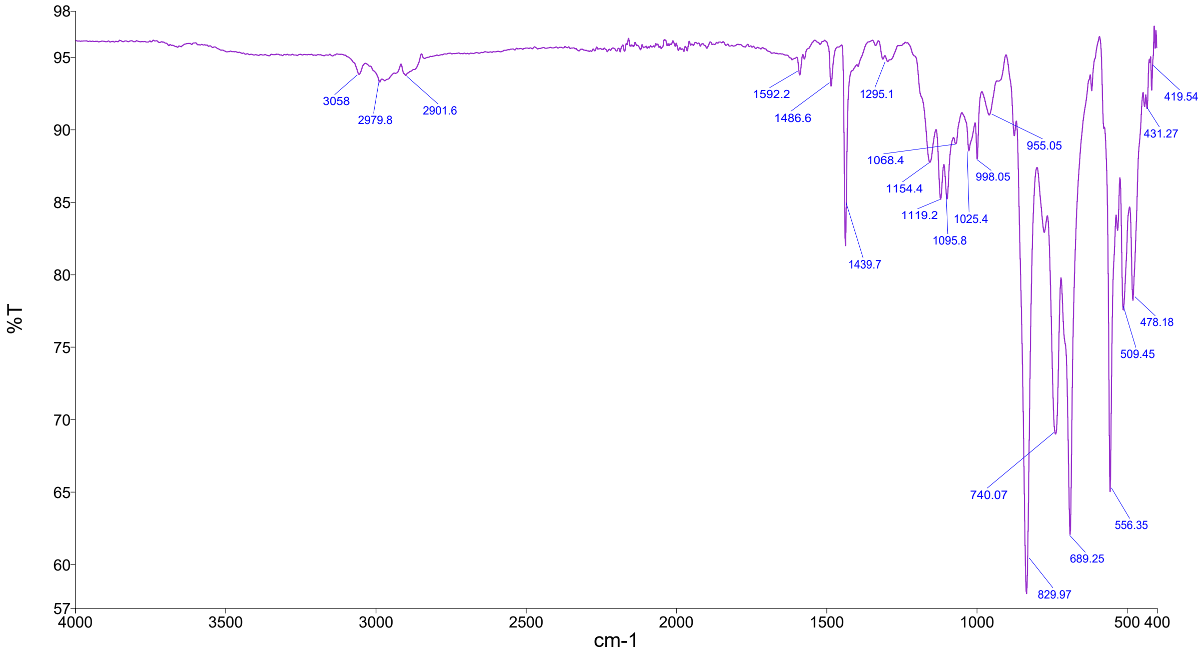

Figure S19. ATR spectrum of [NiCl{PhP(OCH_2PPh_2)$_2$-κ^3P,P,P}][PF_6], **4**.

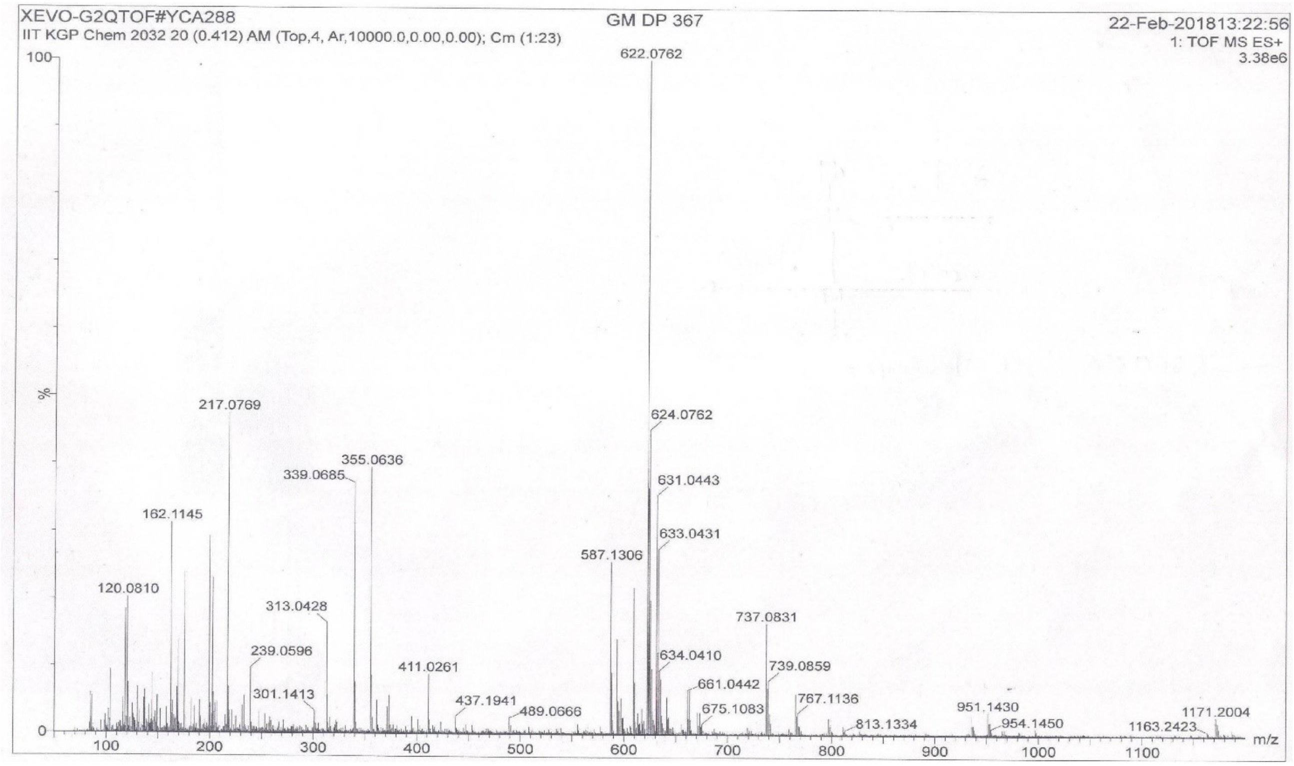

Figure S20. HRMS spectrum of [NiCl{PhP(OCH_2PPh_2)$_2$-κ^3P,P,P}][PF_6], **4**.

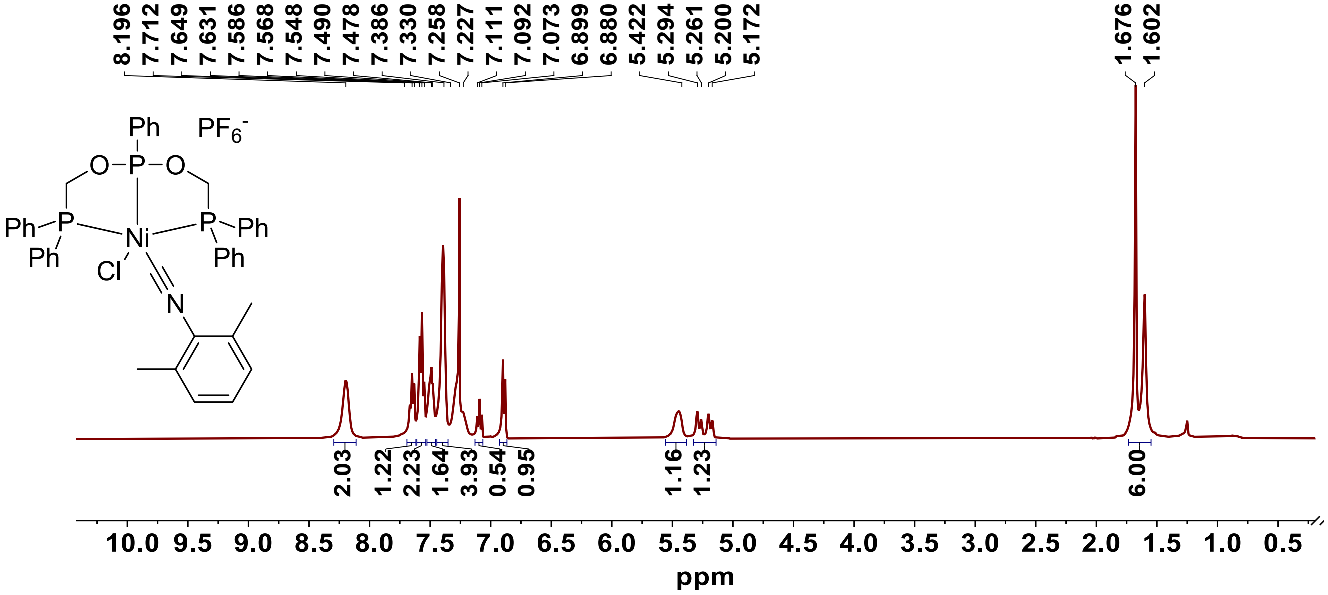

Figure S21. ^{1}H NMR (400 MHz) spectrum of [NiCl{PhP(OCH$_2$PPh$_2$)$_2$-*κ*^{3}*P*,*P*,*P*}(XylylNC)][PF$_6$] **5** in $CDCl_3$.

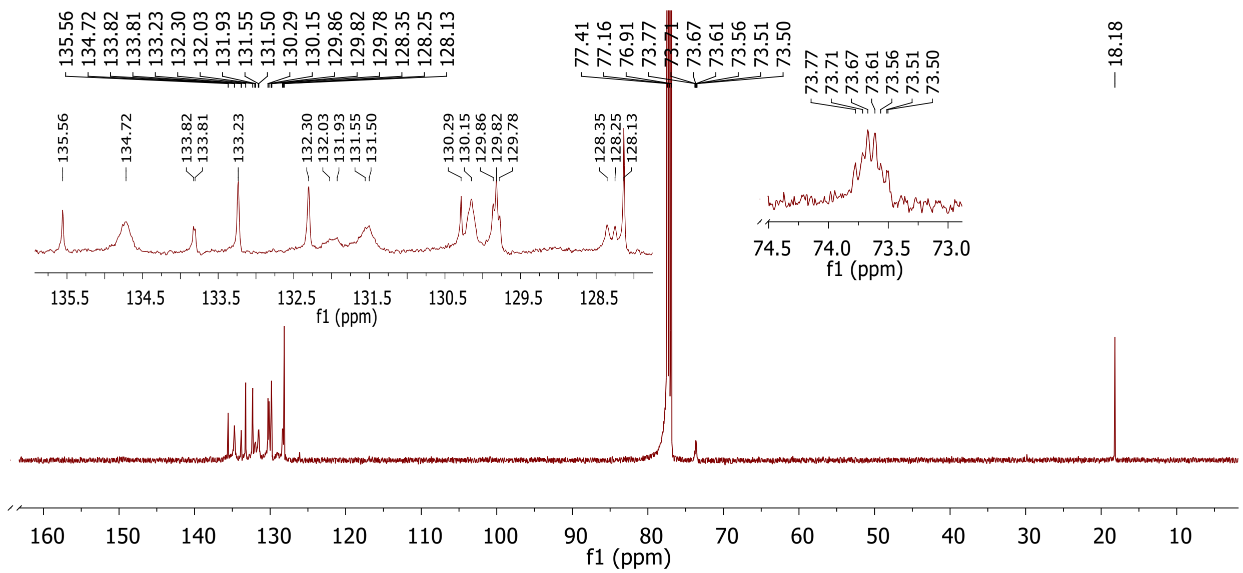

Figure S22. ^{13}C NMR (125.7 MHz) spectrum of [NiCl{PhP(OCH$_2$PPh$_2$)$_2$-κ^3*P*,*P*,*P*}(XylylNC)][PF$_6$] **5** in CDCl$_3$.

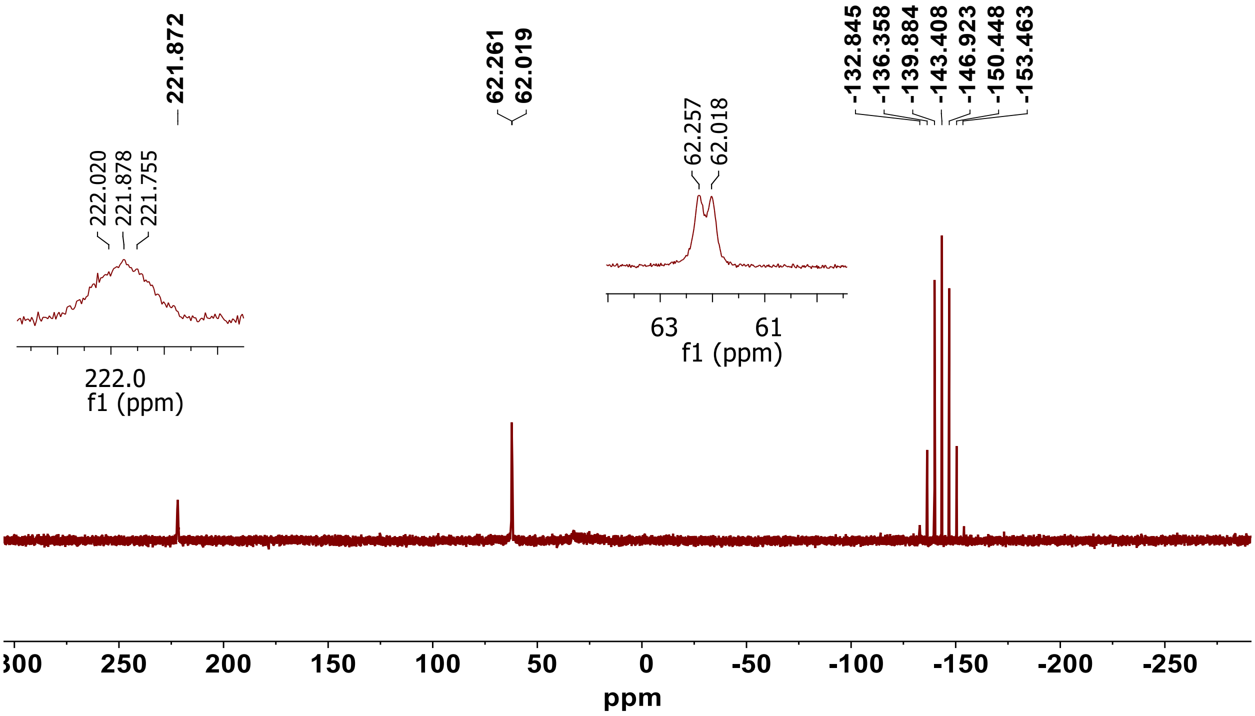

Figure S23. ^{31}P NMR (202.4 MHz) spectrum of [NiCl{PhP(OCH$_2$PPh$_2$)$_2$-κ^3*P*,*P*,*P*}(XylylNC)][PF$_6$] **5** in CDCl$_3$.

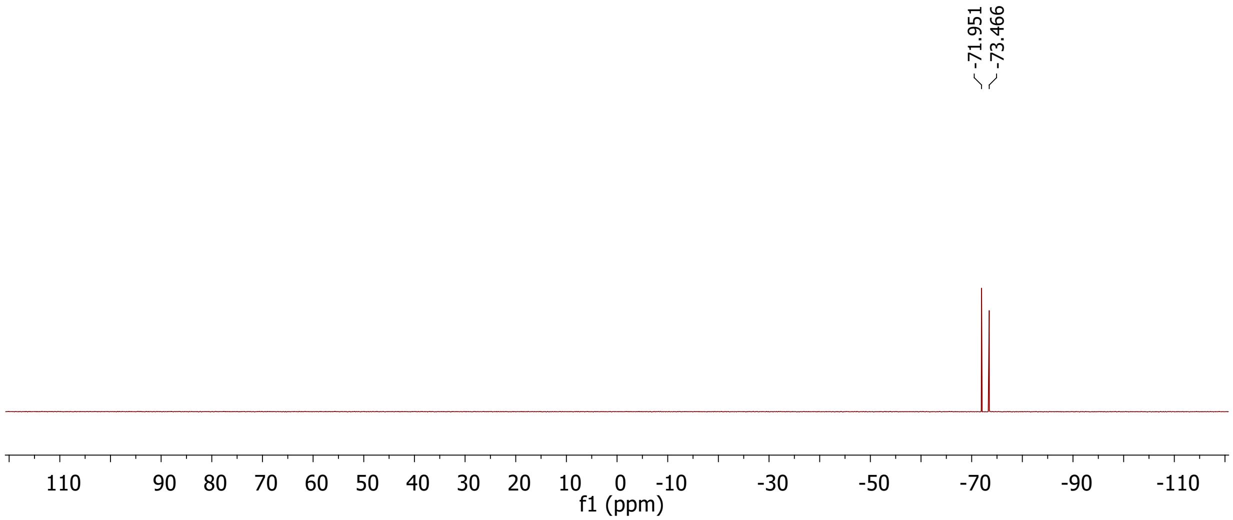

Figure S24. ^{19}F NMR (470.6 MHz) spectrum of [NiCl{PhP(OCH_2PPh_2)$_2$-κ^3*P*,*P*,*P*}(XylylNC)][PF_6] **5** in $CDCl_3$.

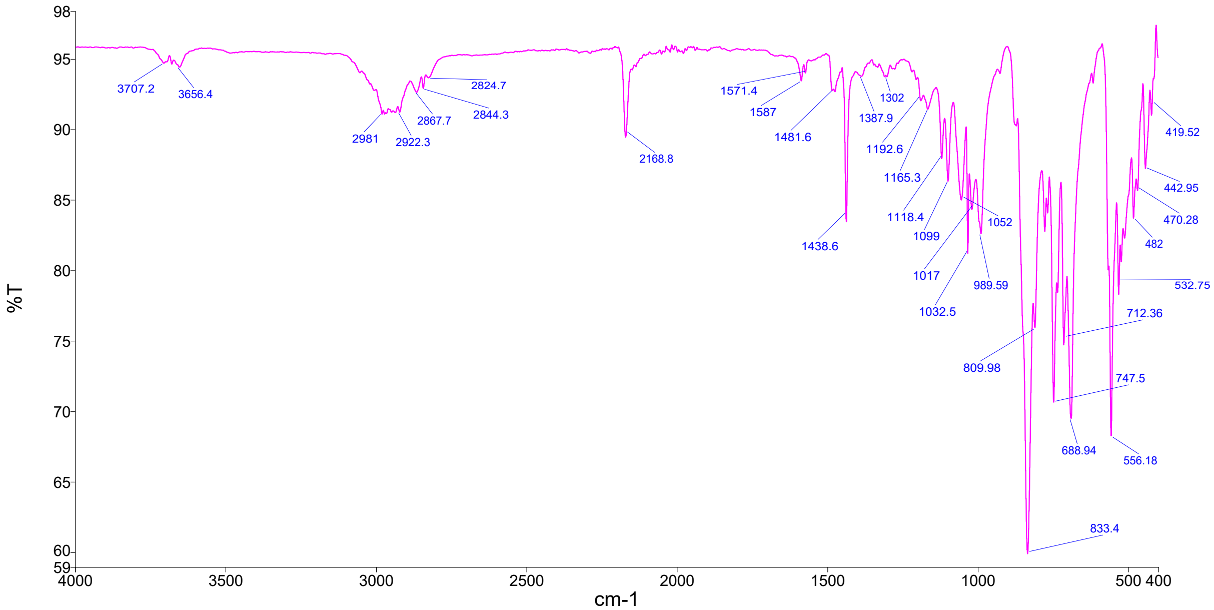

Figure S25. ATR-IR spectrum of [NiCl{PhP(OCH_2PPh_2)$_2$-κ^3*P*,*P*,*P*}(XylylNC)][PF_6] **5**.

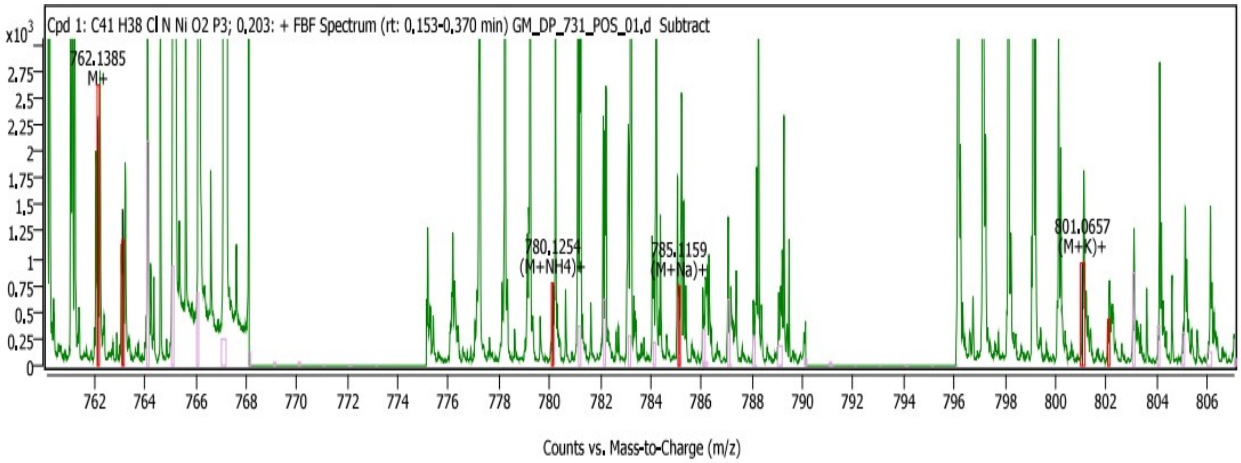

Figure S26. HRMS spectrum of [NiCl{PhP(OCH$_2$PPh$_2$)$_2$-κ^3*P*,*P*,*P*}(XylylNC)][PF$_6$] **5**.

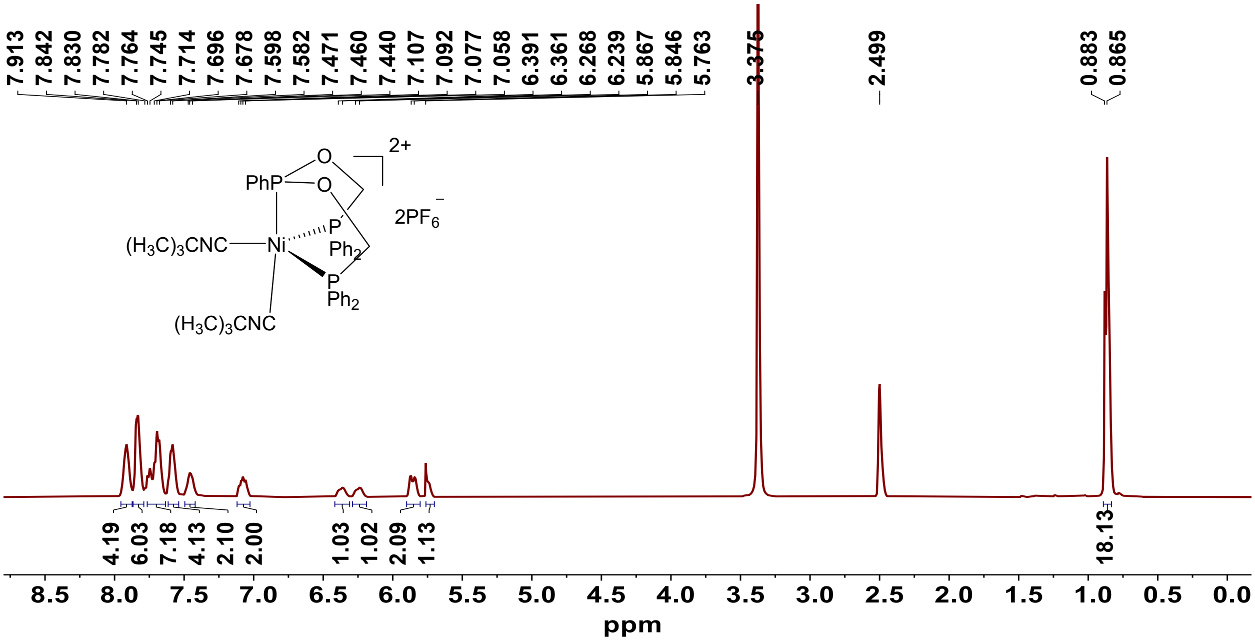

Figure S27. ^{1}H NMR (400 MHz) spectrum of [Ni{PhP(OCH$_2$PPh$_2$)$_2$-κ^3*P*,*P*,*P*}(^{t}BuNC)$_2$][PF$_6$]$_2$ **6** in DMSO−*d$_6$*.

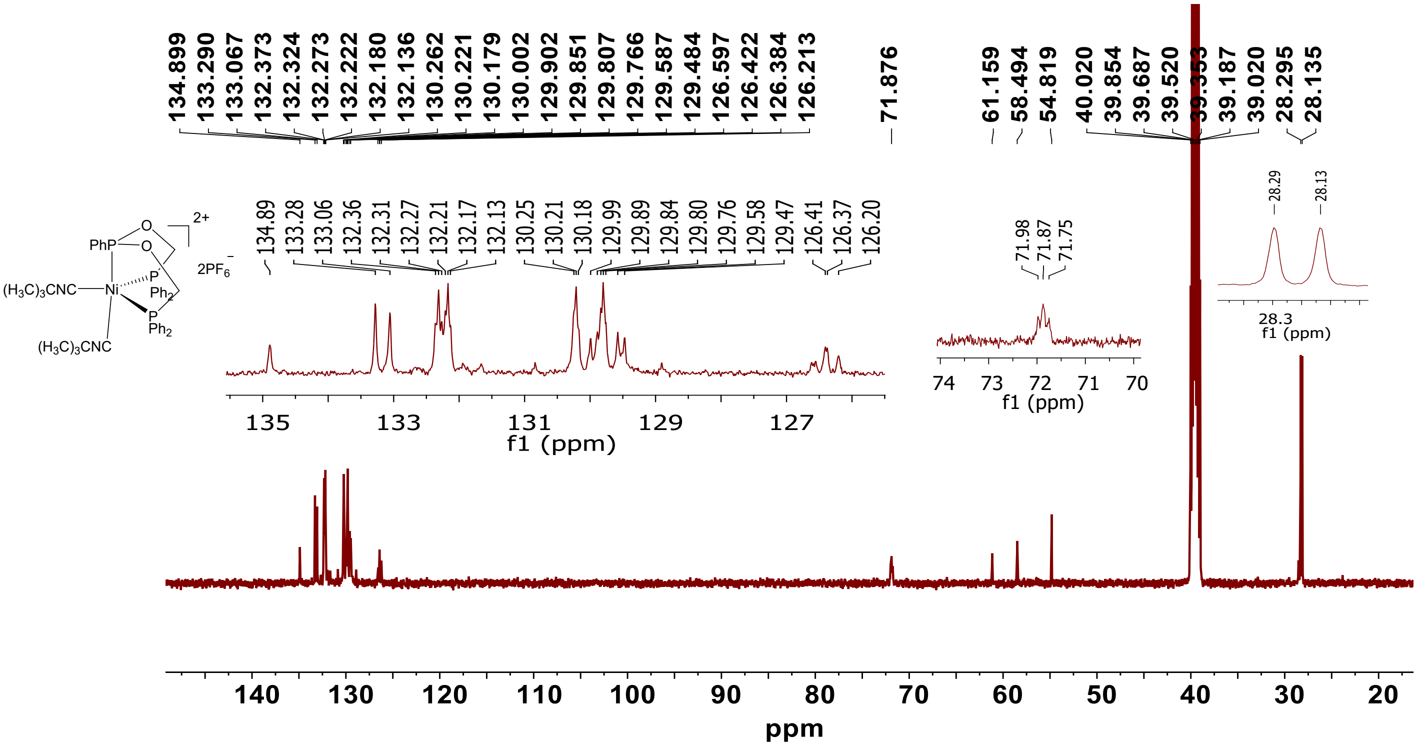

Figure S28. ^{13}C NMR (125.7 MHz) spectrum of $[Ni\{PhP(OCH_2PPh_2)_2\text{-}\kappa^3 P,P,P\}(^tBuNC)_2][PF_6]_2$ **6** in DMSO−d$_6$.

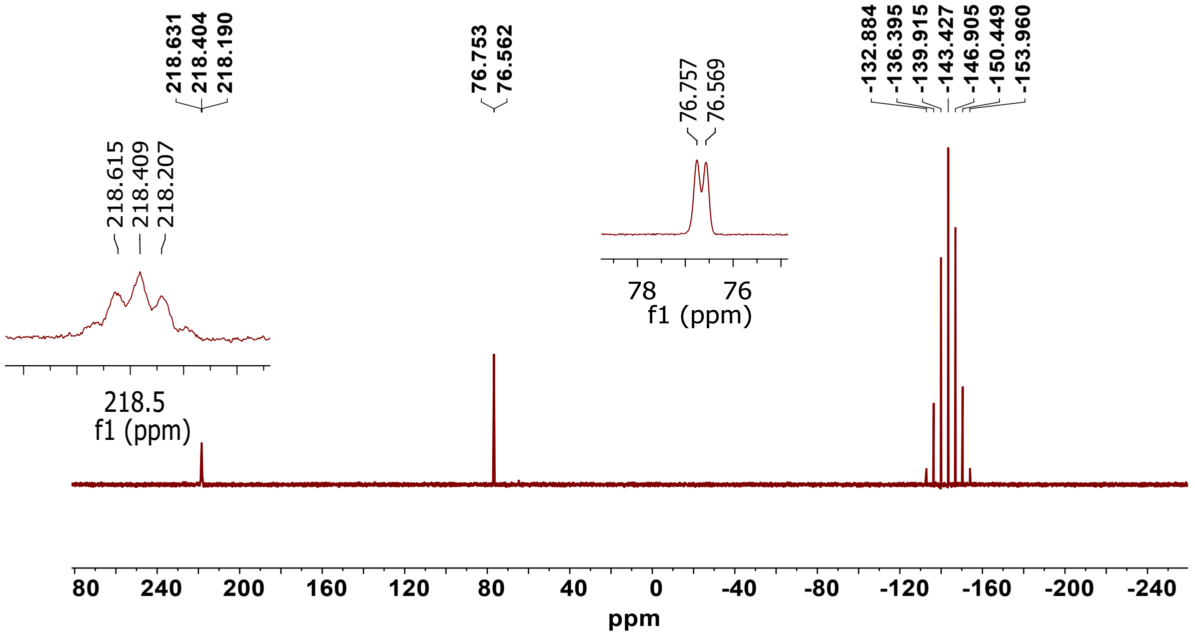

Figure S29. ^{31}P NMR (202.4 MHz) spectrum of $[Ni\{PhP(OCH_2PPh_2)_2\text{-}\kappa^3 P,P,P\}(^tBuNC)_2][PF_6]_2$ **6** in DMSO−d_6.

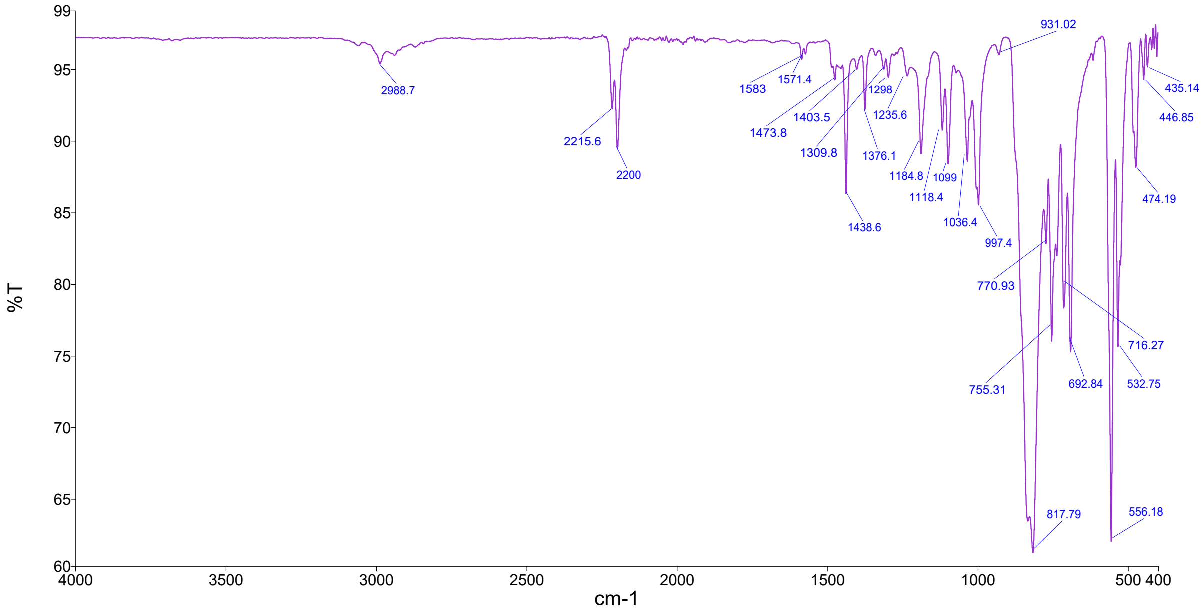

Figure S30. ATR-IR spectrum of [Ni{PhP(OCH_2PPh_2)$_2$-κ^3*P*,*P*,*P*}(^{t}BuNC)$_2$][PF_6]$_2$ **6**.

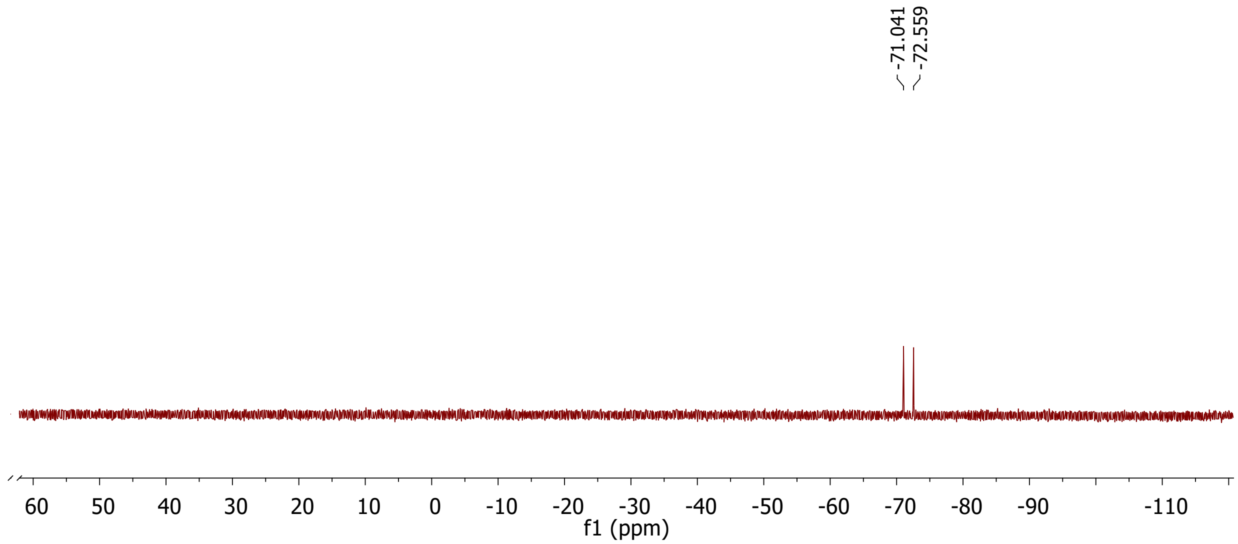

Figure 31. ^{19}F NMR (470.6 MHz) spectrum of [Ni{PhP(OCH_2PPh_2)$_2$-κ^3*P*,*P*,*P*}(^{t}BuNC)$_2$][PF_6]$_2$ **6** in $CDCl_3$.

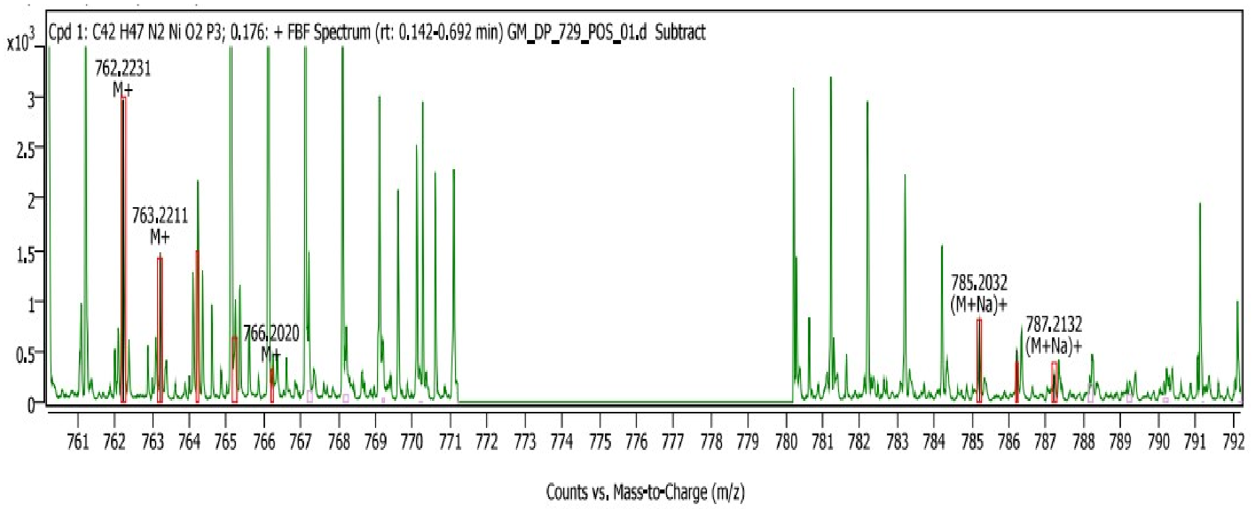

Figure S32. HRMS spectrum of [Ni{PhP(OCH$_2$PPh$_2$)$_2$-κ^3*P*,*P*,*P*}(^{t}BuNC)$_2$][PF$_6$]$_2$ **6**.

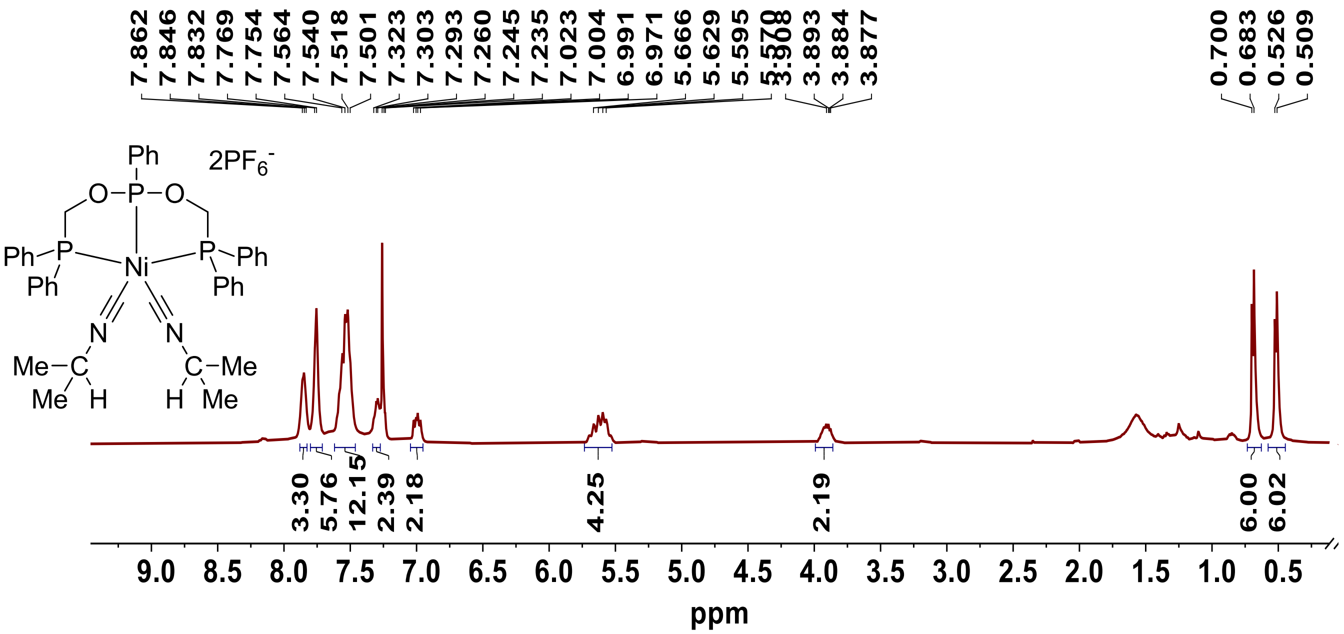

Figure S33. ^{1}H NMR (400 MHz) spectrum of [Ni{PhP(OCH$_2$PPh$_2$)$_2$-κ^3*P*,*P*,*P*}(^{i}PrNC)$_2$][PF$_6$]$_2$ **7** in CDCl$_3$.

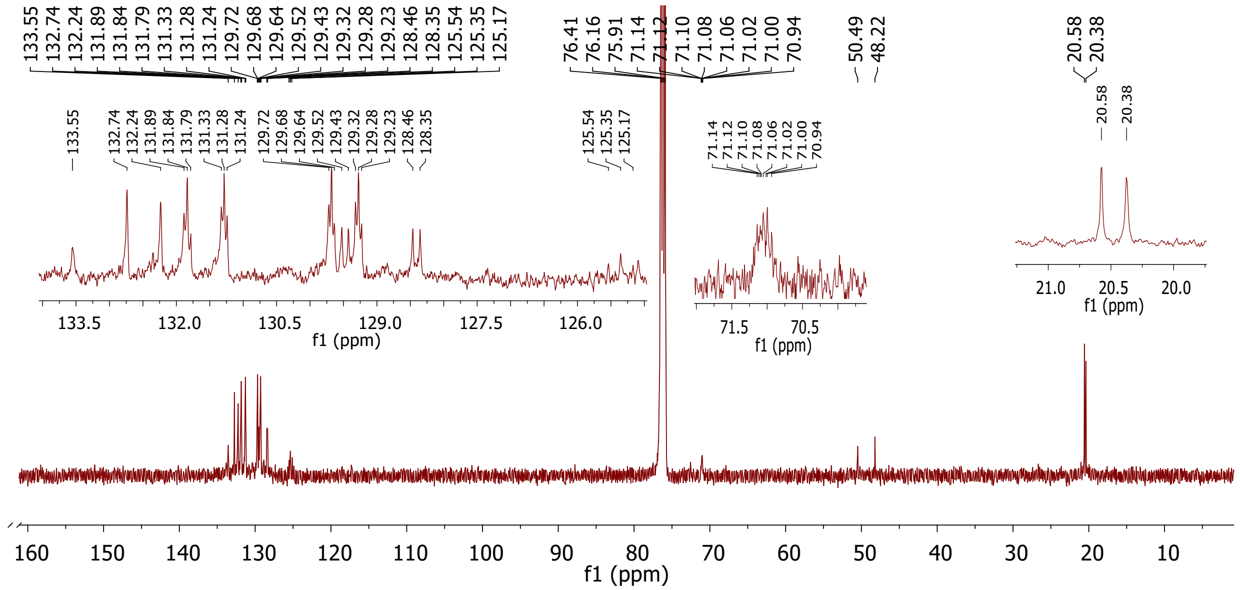

Figure S34. ^{13}C NMR (125.7 MHz) spectrum of [Ni{PhP(OCH$_2$PPh$_2$)$_2$-κ^3*P*,*P*,*P*}(^{i}PrNC)$_2$][PF$_6$]$_2$ **7** in CDCl$_3$.

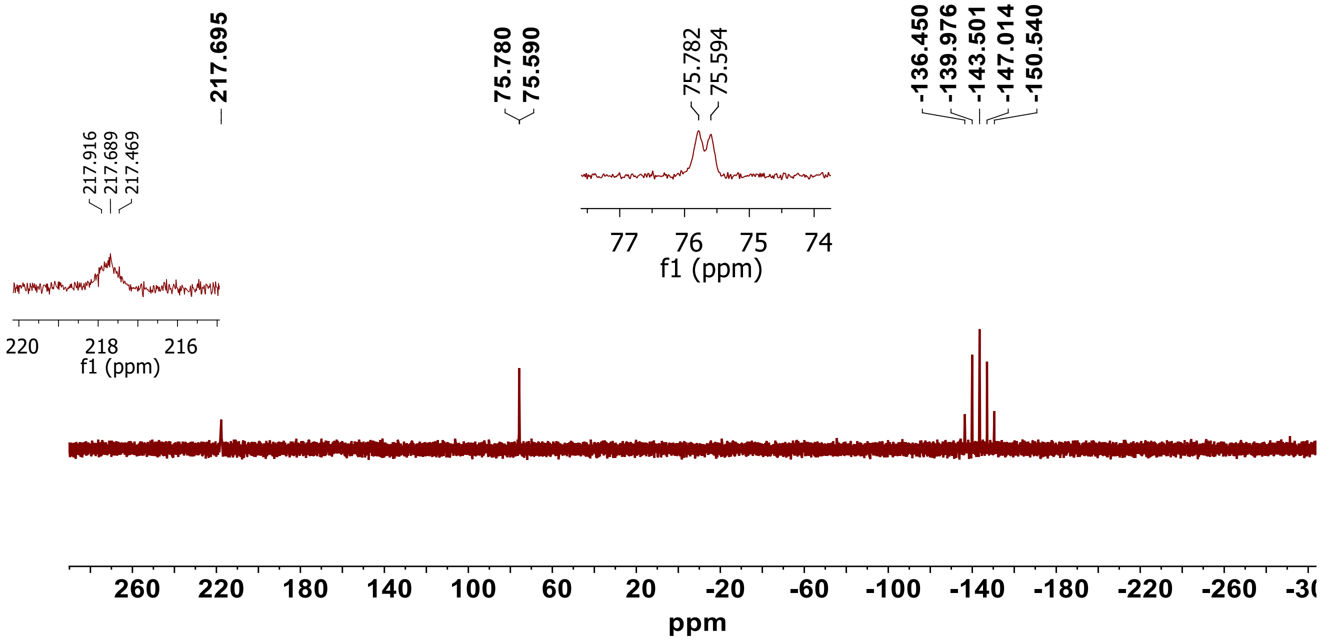

Figure S35. ^{31}P NMR (202.4 MHz) spectrum of [Ni{PhP(OCH$_2$PPh$_2$)$_2$-κ^3*P*,*P*,*P*}(^{i}PrNC)$_2$][PF$_6$]$_2$ **7** in CDCl$_3$.

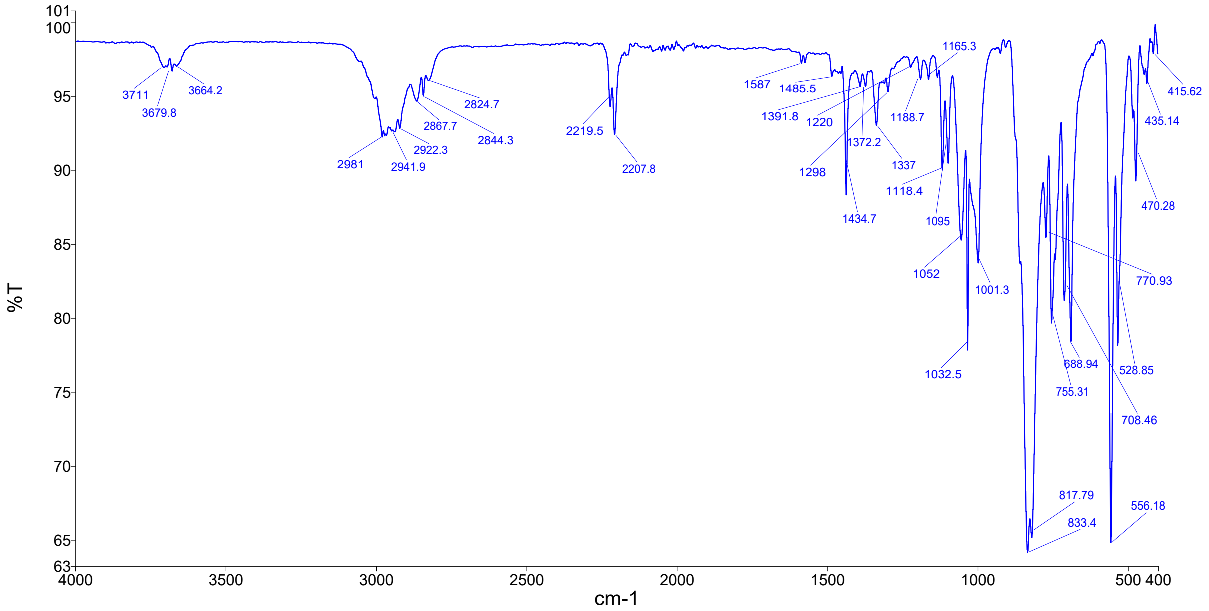

Figure S36. ATR-IR spectrum of [Ni{PhP(OCH_2PPh_2)$_2$-κ^3P,P,P}(^{i}PrNC)$_2$][PF_6]$_2$ **7**.

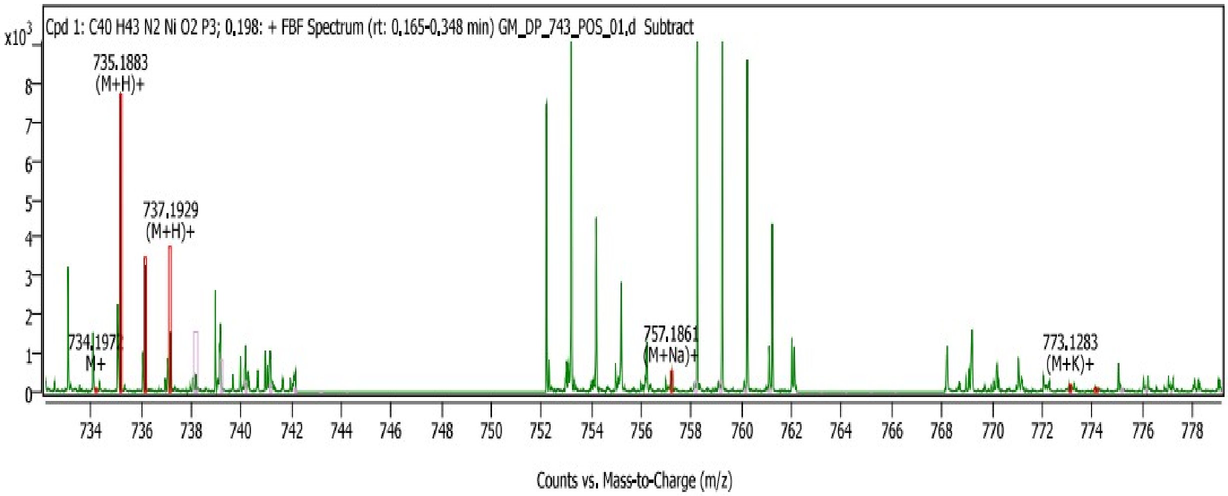

Figure S37. HRMS spectrum of [Ni{PhP(OCH_2PPh_2)$_2$-κ^3P,P,P}(^{i}PrNC)$_2$][PF_6]$_2$ **7**.

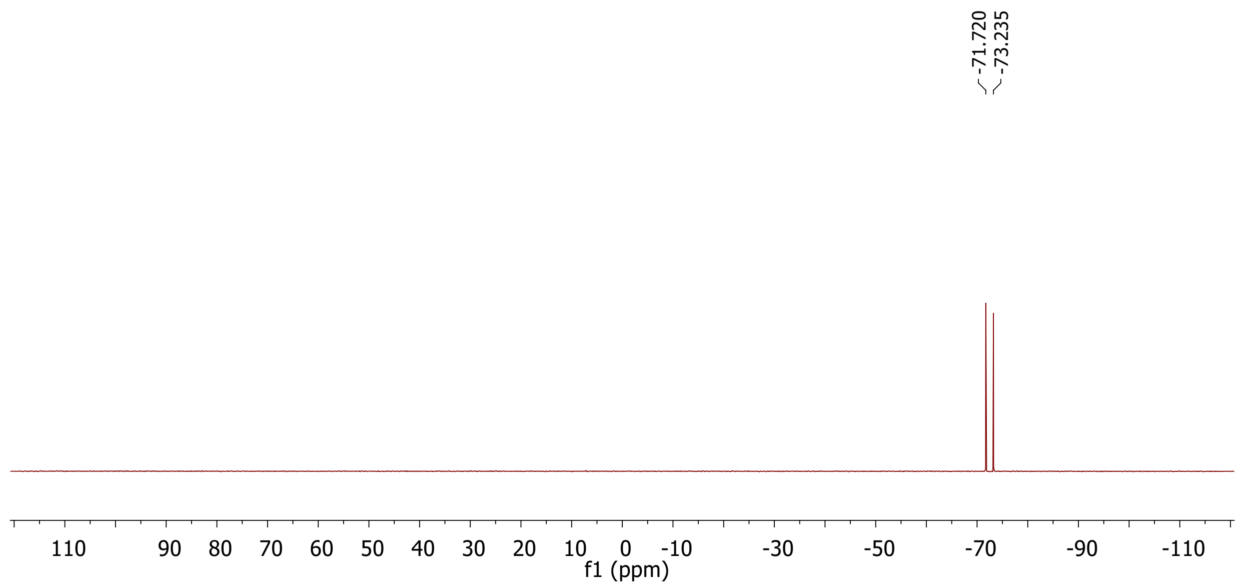

Figure S38. ^{19}F NMR (470.6 MHz) spectrum of [Ni{PhP(OCH_2PPh_2)$_2$-$\kappa^3 P,P,P$}(^{i}PrNC)$_2$][PF_6]$_2$ **7** in $CDCl_3$.

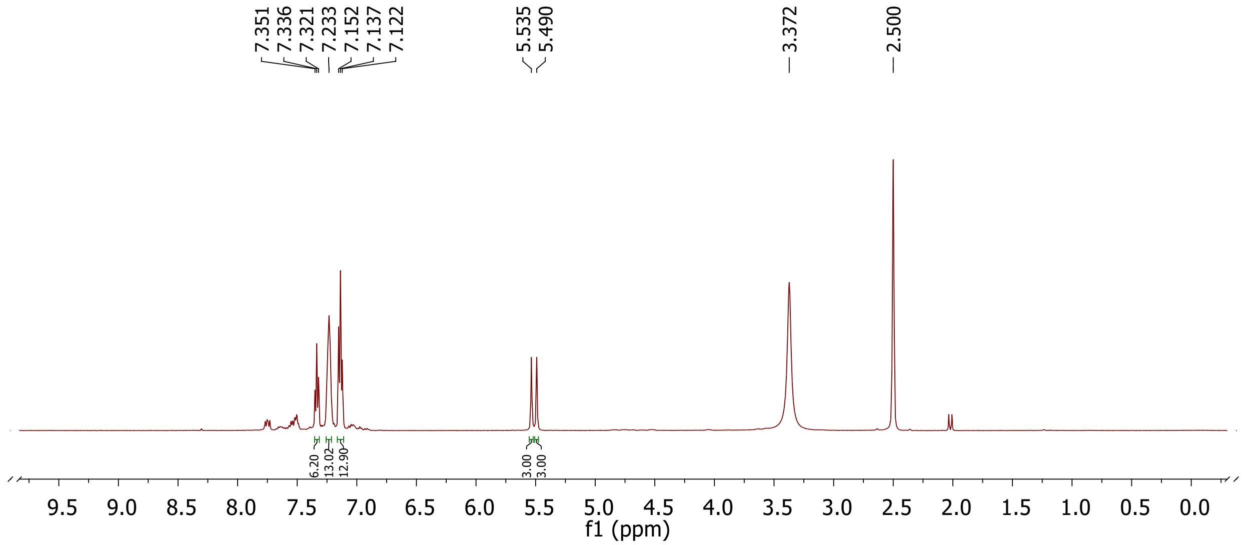

Figure S39. ^{1}H NMR (500 MHz) spectrum of [NiCl{P(OCH_2PPh_2)$_3$-$\kappa^4 P,P,P,P$}][PF_6], **8** in DMSO−d_6.

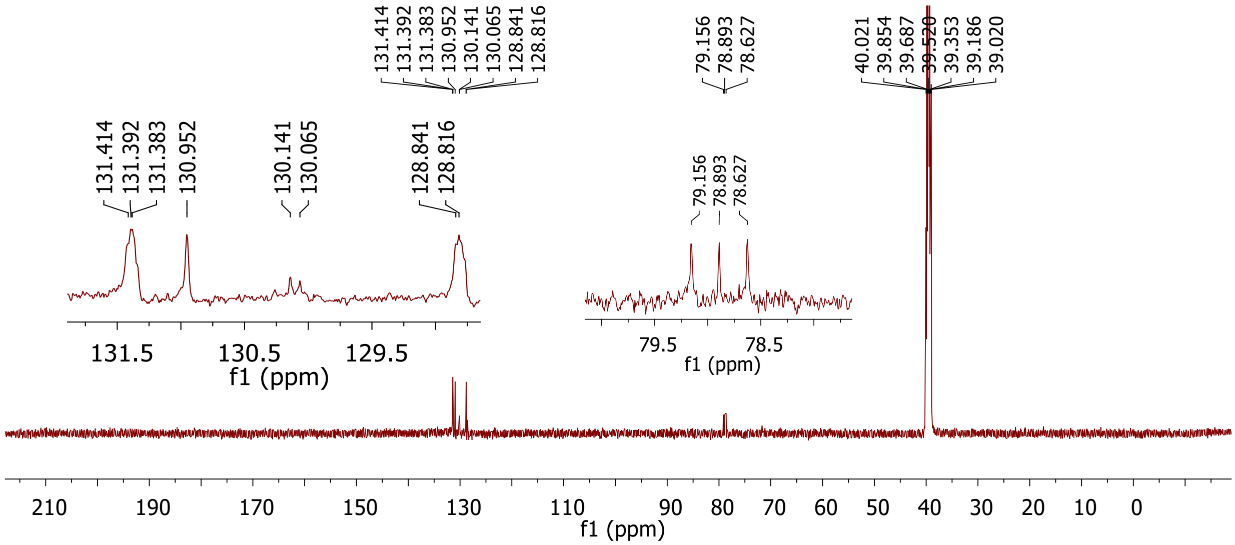

Figure S40. ^{13}C NMR (125.7 MHz) spectrum of [NiCl{P(OCH_2PPh_2)$_3$-κ^4*P*,*P*,*P*,*P*}][PF_6], **8** in DMSO−d_6.

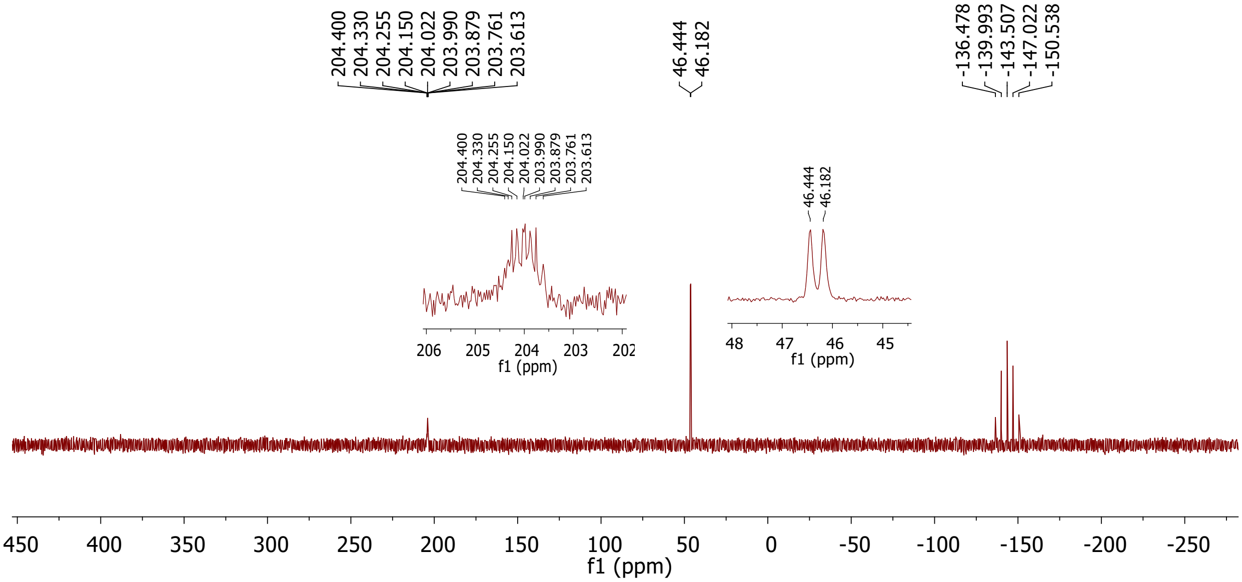

Figure S41. ^{31}P NMR (202.4 MHz) spectrum of [NiCl{P(OCH_2PPh_2)$_3$-κ^4*P*,*P*,*P*,*P*}][PF_6], **8** in DMSO−d_6.

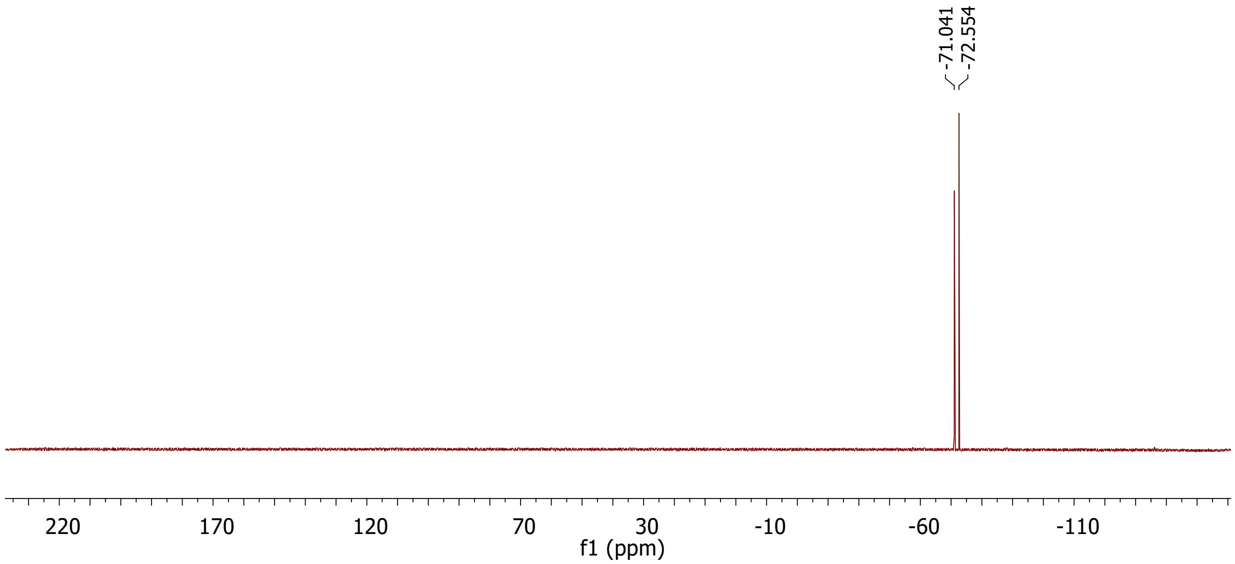

Figure S42. ^{19}F NMR (470.6 MHz) spectrum of [NiCl{P(OCH_2PPh_2)$_3$-κ^4*P*,*P*,*P*,*P*}][PF_6], **8** in DMSO−d_6.

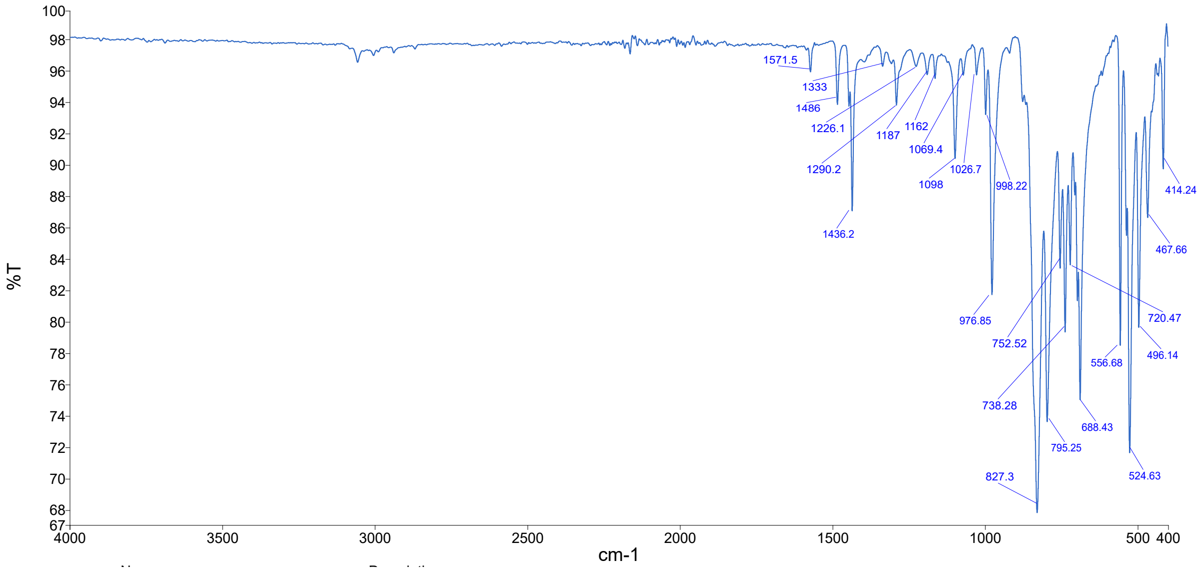

Figure S43. ATR-IR spectrum of [NiCl{P(OCH_2PPh_2)$_3$-κ^4*P*,*P*,*P*,*P*}][PF_6], **8**.

Name	GM_DP_1020	Rack Pos.		Instrument	IITKGP	Operator	
Inj. Vol. (ul)	2	Plate Pos.		IRM Status	Success		
Data File	GM_DP_1020_POS_01.d	Method (Acq)	MS SCAN POS_ORGANOMETALLIC S_01.m	Comment		Acq. Time (Local)	11/11/2021 4:12:31 PM (UTC+05:30)

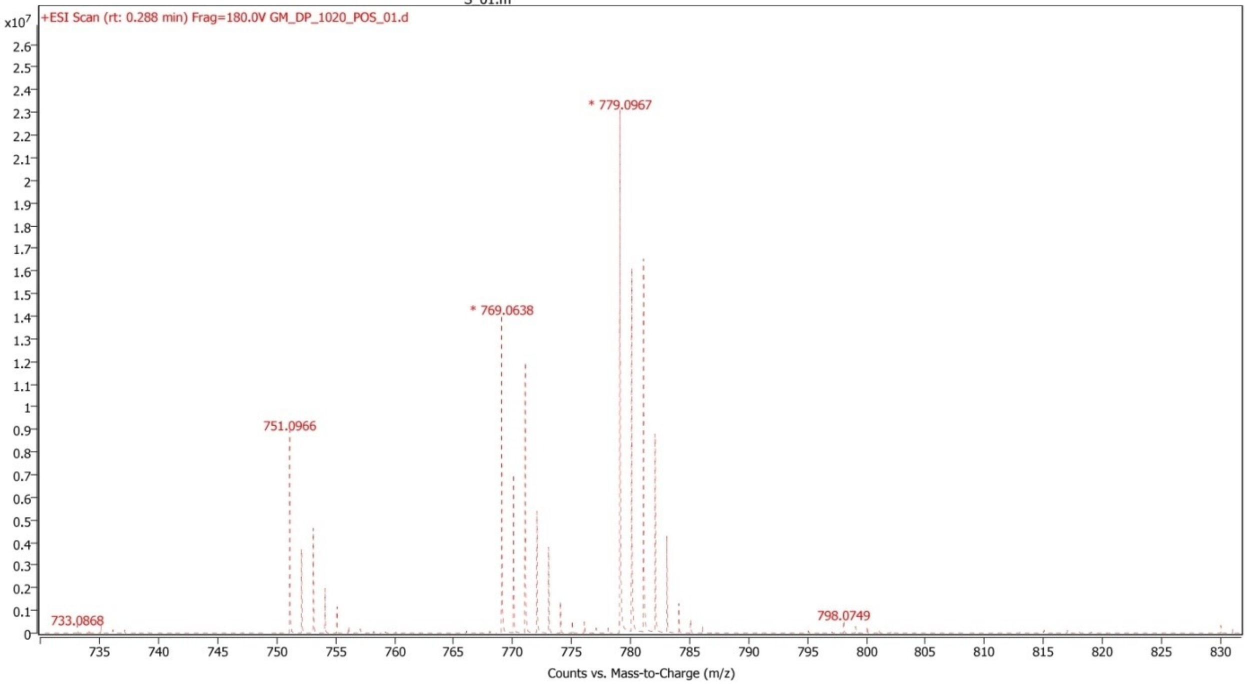

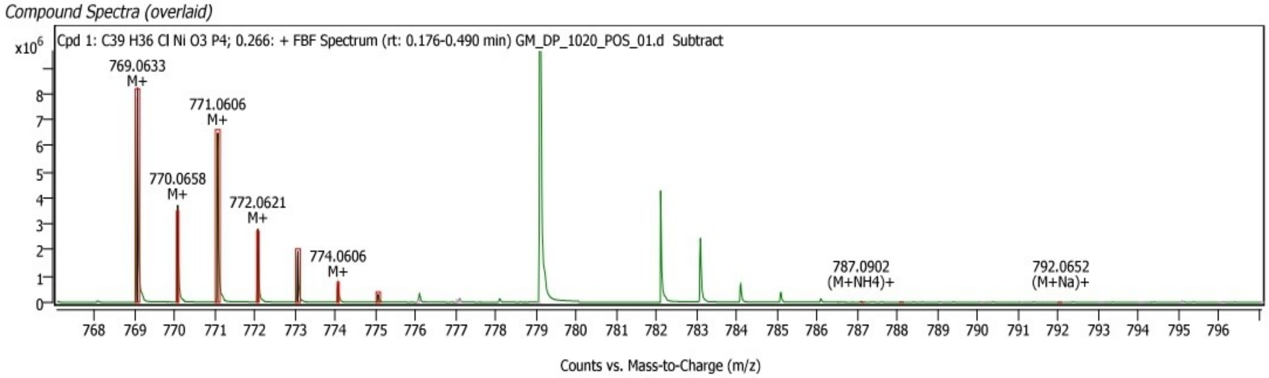

Figure S44. HRMS spectrum of $[NiCl\{P(OCH_2PPh_2)_3\text{-}\kappa^4 P,P,P,P\}][PF_6]$, **8**.

X-ray Structures

Single crystal X-ray diffraction data collections for all complexes were performed using Bruker-APEX-II CCD or APEX3 D8 QUEST PHOTON-II diffractometer with graphite monochromated Mo Kα radiation (λ = 0.71073 Å). The space group for every structure was obtained by XPREP program. Using the GUI WingX, the structures were solved by SHELXT[1] which successfully located most of the nonhydrogen atoms. Subsequently, least-squares refinements were carried out on F^2 using SHELXL Version 2018/3[2] to locate the remaining nonhydrogen atoms. Non-hydrogen atoms were refined with anisotropic displacement parameters. Hydrogen atoms attached to carbon atoms were fixed in calculated positions. In the case of structure **6**, one of the PF_6^- anions and the solvent of crystallization CH_2Cl_2 are disordered and they were successfully resolved using SADI, EADP and SIMU restraints. The refinement data for all the structures are summarized in Table S1. Crystallographic data were deposited with the Cambridge Crystallographic Data Centre, CCDC, 12 Union Road, Cambridge CB21EZ, UK. These data can be obtained free of charge upon quoting the depository numbers CCDC 2112704-2112708 from web interface (at http://www.ccdc.cam.ac.uk).

Table S1. Crystallographic data for complexes **3-7**.

	**3**	**4**	**5**	**6**	**7**	**8**
Empirical formula	$C_{33}H_{31}Cl_4NiO_2P_3$	$C_{32}H_{29}ClF_6NiO_2P_4$	$C_{41}H_{38}ClF_6NNiO_2P_4$	$C_{43}H_{49}Cl_2F_{12}N_2NiO_2P_5$	$C_{41}H_{45}Cl_2F_{12}N_2NiO_2P_5$	$C_{39}H_{36}ClF_6$ NiO_3P_5
Formula weight	753.00	777.59	908.76	1138.30	1110.25	915.69
Wavelength (Å)	0.71073	0.71073	0.71073	0.71073	0.71073	0.71073
Temperature (K)	296	297	296	296	100	296
Crystal system	Monoclinic	Triclinic	Monoclinic	Monoclinic	Monoclinic	Cubic
Space group	$P2_1/c$	$P{-}1$	$P2_1/c$	$P2_1/n$	$P2_1/n$	$P2_13$
a/Å	11.2965(8)	12.0853(7)	10.4860(7)	11.235(2)	10.6099(10)	16.0943(9)
b/Å	23.8196(17)	12.7608(8)	13.2706(9)	22.991(4)	22.192(2)	16.0943(9)
c/Å	12.6524(8)	12.9324(7)	29.888(2)	20.206(4)	20.122(2)	16.0943(9)
α/degree	90	106.472(3)	90	90	90	90
β/degree	98.894(3)	94.713(3)	91.166(3)	97.139(6)	91.369(3)	90
γ/degree	90	115.252(2)	90	90	90	90
Vol (Å^3)	3363.5(4)	1681.68(17)	4158.2(5)	5178.8(16)	4736.5(8)	4168.8(7)
Z	4	2	4	4	4	4
D_{calcd}, g cm^{-3}	1.487	1.536	1.452	1.460	1.557	1.459
μ/mm^{-1}	1.067	0.910	0.748	0.712	0.777	0.784
F(000)	1544	792	1864	2328	2264	1872
θ range (degree)	2.362 to 30.518	2.944 to 28.315	2.350 to 27.199	2.216 to 24.997	1.835 to 26.413	2.192 to 27.146
Limiting Indices	$-16 \le h \le 16$,<br>$-32 \le k \le 34$,<br>$-18 \le l \le 18$	$-16 \le h \le 15$,<br>$-14 \le k \le 17$,<br>$-17 \le l \le 17$	$-13 \le h \le 13$,<br>$-17 \le k \le 16$,<br>$-38 \le l \le 38$	$-13 \le h \le 13$,<br>$-27 \le k \le 27$,<br>$-24 \le l \le 24$	$-13 \le h \le 13$,<br>$-24 \le k \le 27$,<br>$-24 \le l \le 25$	-20<=h<=20<br>-20<=k<=20<br>-20<=l<=20
Total/ unique no. of reflns	57694 / 10198	23584 / 8250	81157 / 9234	105309 / 9088	59211 / 9639	62912 / 3107
R_{int}	0.0961	0.0400	0.0845	0.0911	0.0649	0.0979
Data/ restr./params.	10198/0/388	8250/0/415	9234/0/507	9088/197/546	9639/0/590	3107 / 0 / 166
GOF (F^2)	1.041	1.044	1.068	1.074	1.030	1.059
$R1$, $wR2$	0.0598, 0.0967	0.0380, 0.0947	0.0540, 0.1319	0.0658, 0.1895	0.0382, 0.0928	0.0630, 0.1675
R indices (all data) $R1$, $wR2$	0.1490, 0.1270	0.0610, 0.1034	0.1139, 0.1781	0.0943, 0.2038	0.0498, 0.0995	0.0924, 0.1973
Largest different peak and hole (e Å^{-3})	0.581, −0.552	0.622, −0.558	1.243, −0.466	1.079, −0.641	0.706, −0.415	0.746 and -0.594

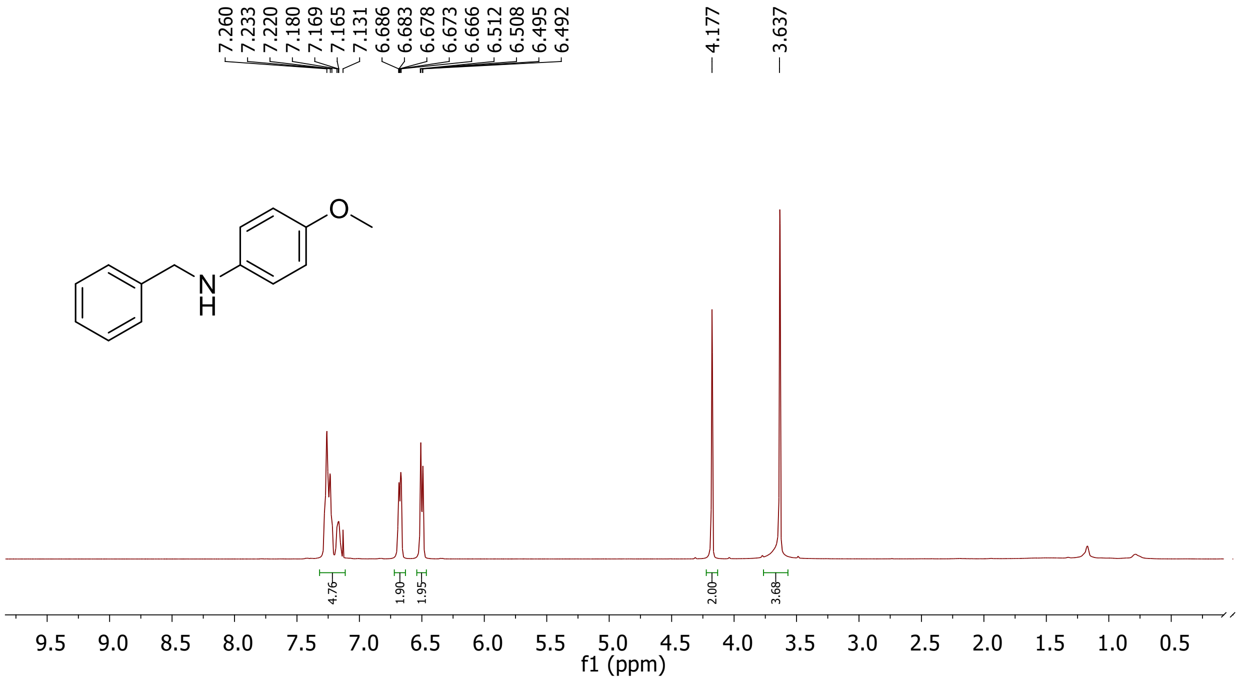

Figure S45. ^{1}H NMR (500 MHz) spectrum of *N*-benzyl-4-methoxyaniline in $CDCl_3$.

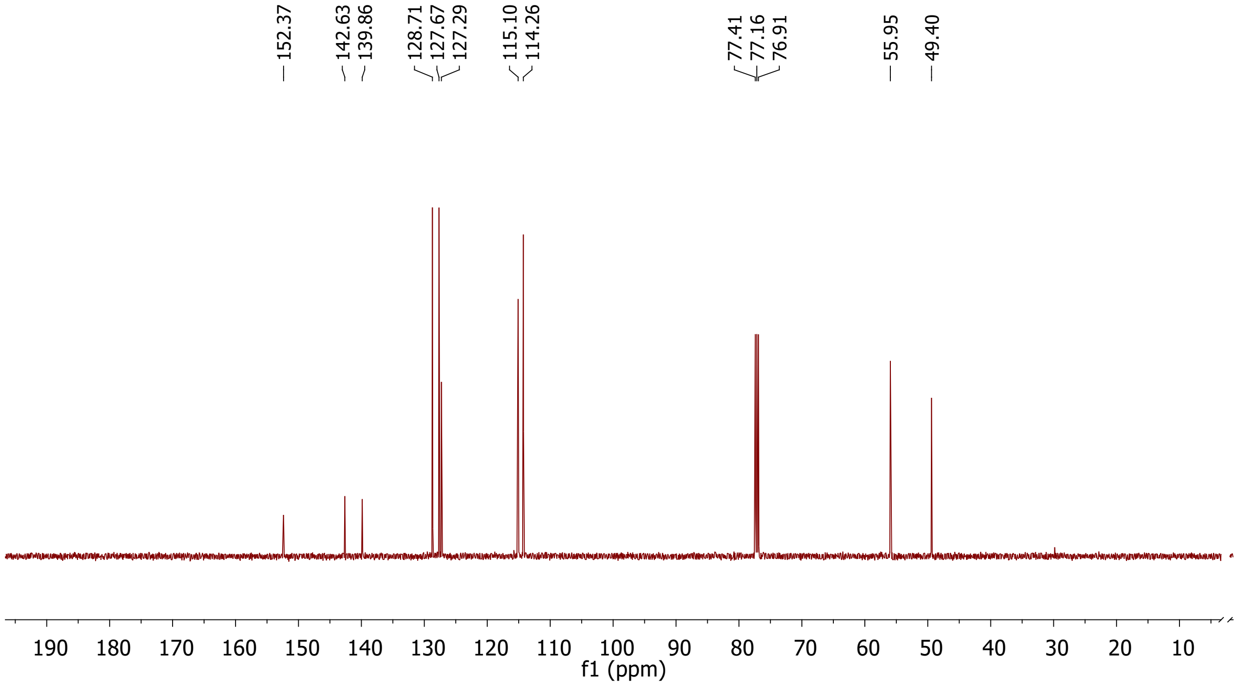

Figure S46. $^{13}C\{^{1}H\}$ NMR (125.7 MHz) spectrum of *N*-benzyl-4-methoxyaniline in $CDCl_3$.

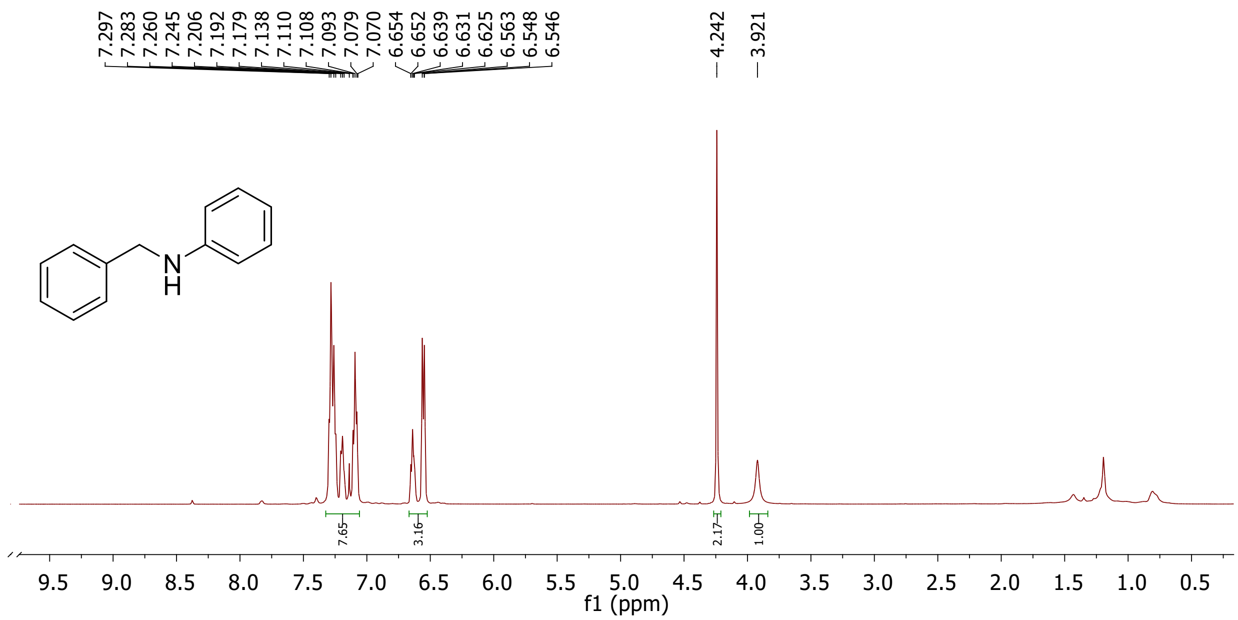

Figure S47. ^{1}H NMR (500 MHz) spectrum of *N*-benzylaniline in $CDCl_3$.

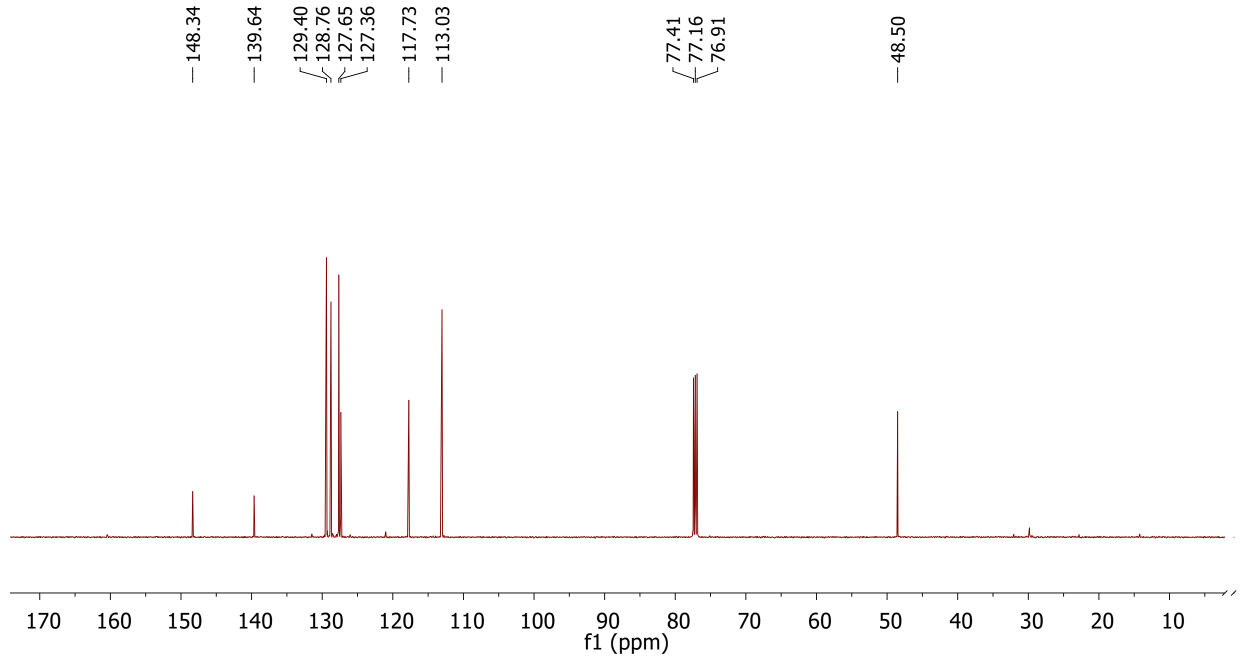

Figure S48. $^{13}C\{^{1}H\}$ NMR (125.7 MHz) spectrum of *N*-benzylaniline in $CDCl_3$.

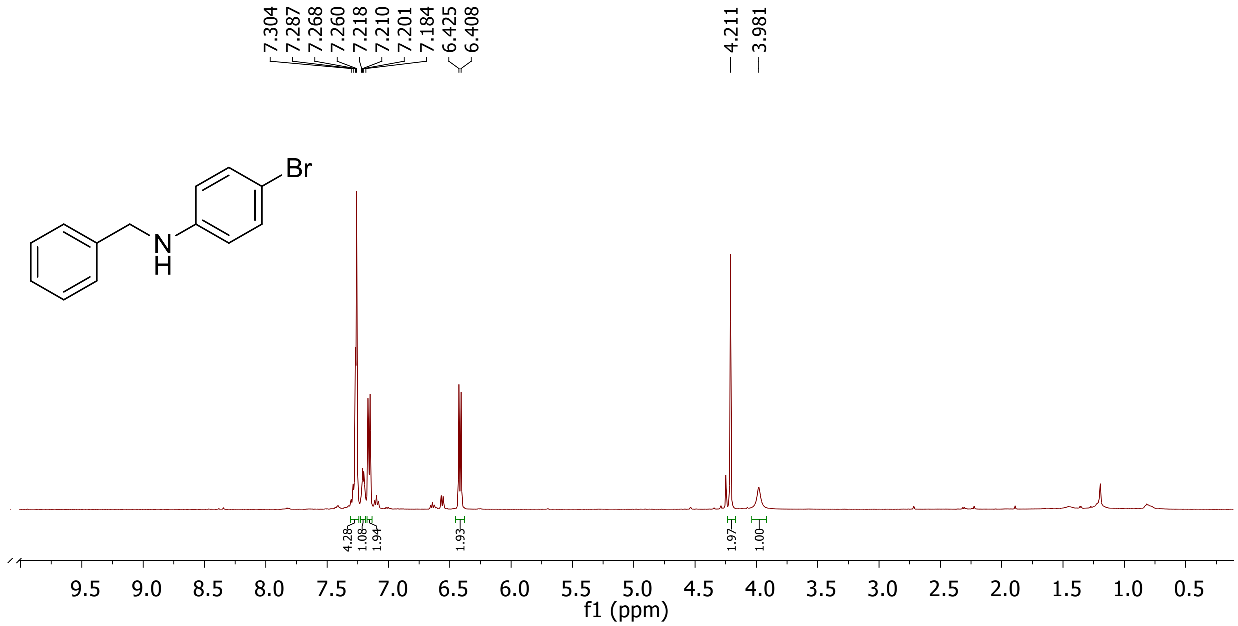

Figure S49. ^{1}H NMR (500 MHz) spectrum of *N*-benzyl-4-bromoaniline in $CDCl_3$.

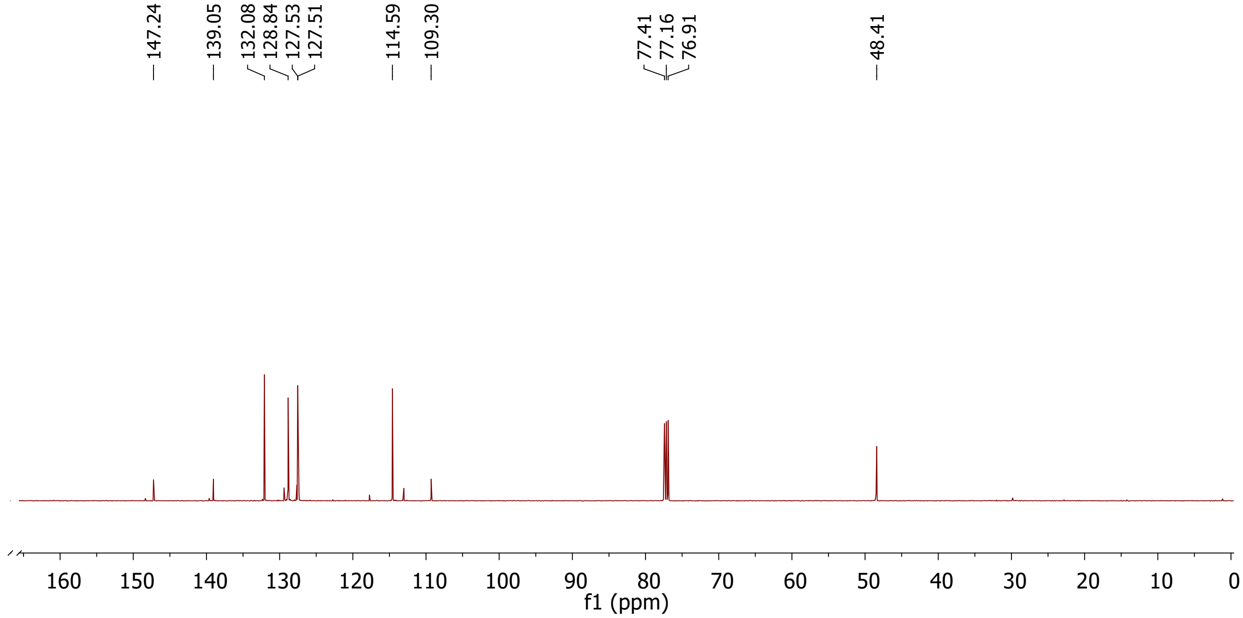

Figure S50. $^{13}C\{^{1}H\}$ NMR (125.7 MHz) spectrum of *N*-benzyl-4-bromoaniline in $CDCl_3$.

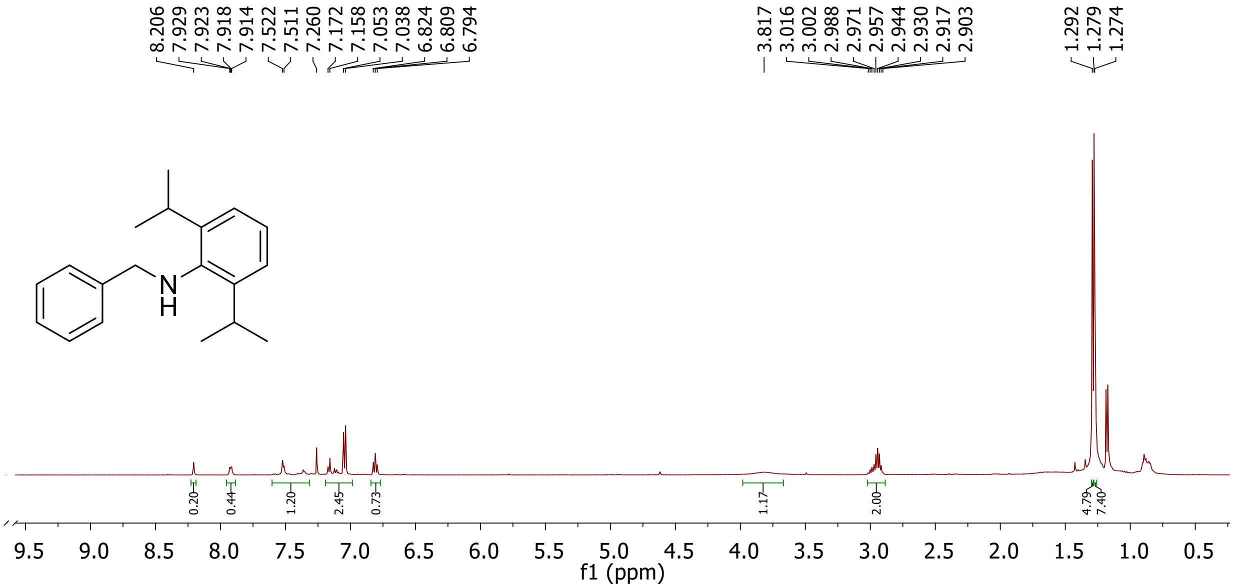

Figure S51. ^{1}H NMR (500 MHz) spectrum of *N*-benzyl-2,6-diisopropylaniline in $CDCl_3$.

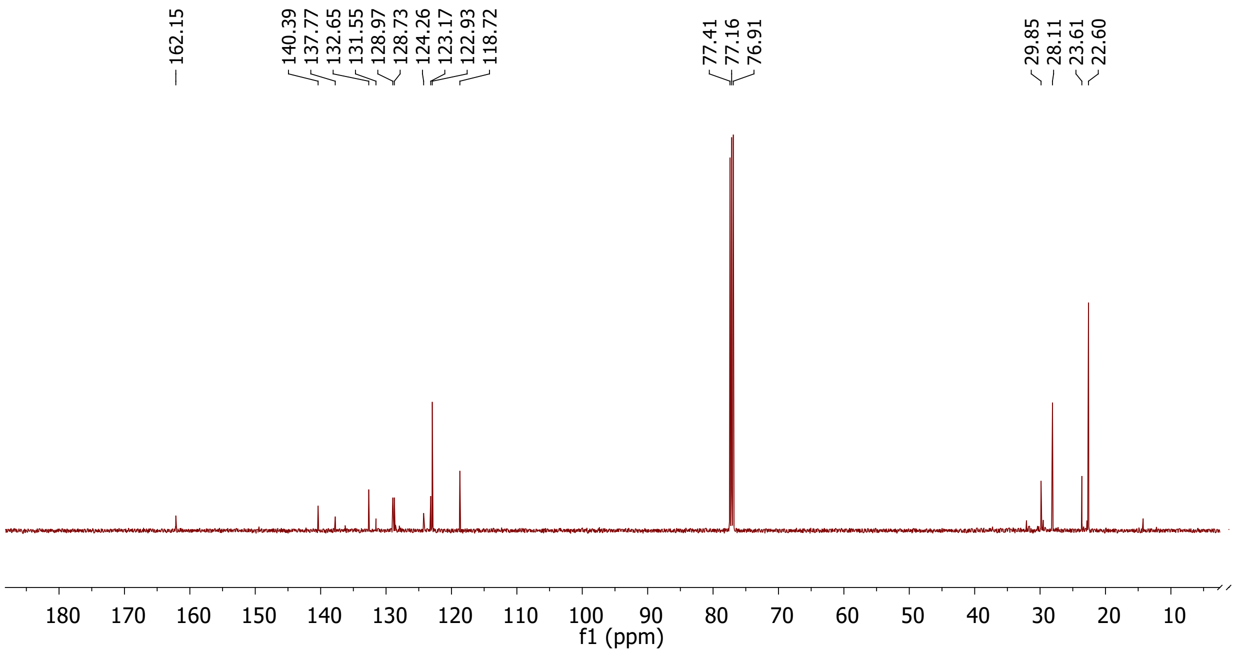

Figure S52. $^{13}C\{^{1}H\}$ NMR (125.7 MHz) spectrum of *N*-benzyl-2,6-diisopropylaniline in $CDCl_3$.

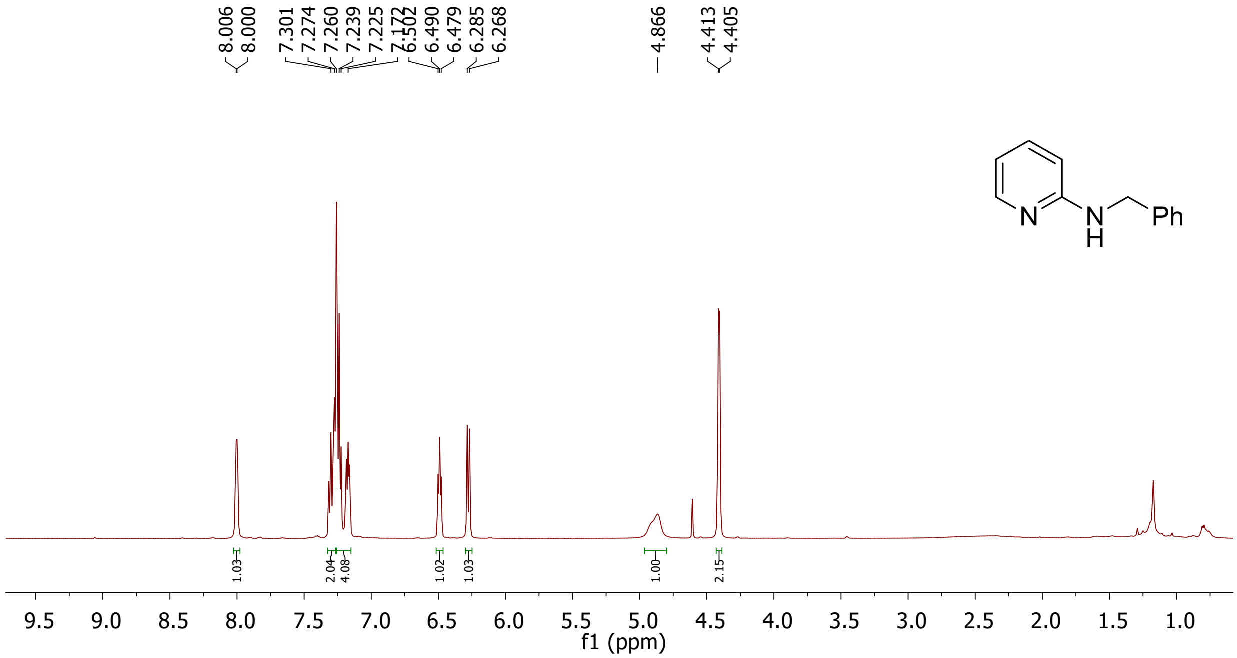

Figure S53. ^{1}H NMR (500 MHz) spectrum of *N*-benzylpyridin-2-amine in $CDCl_3$.

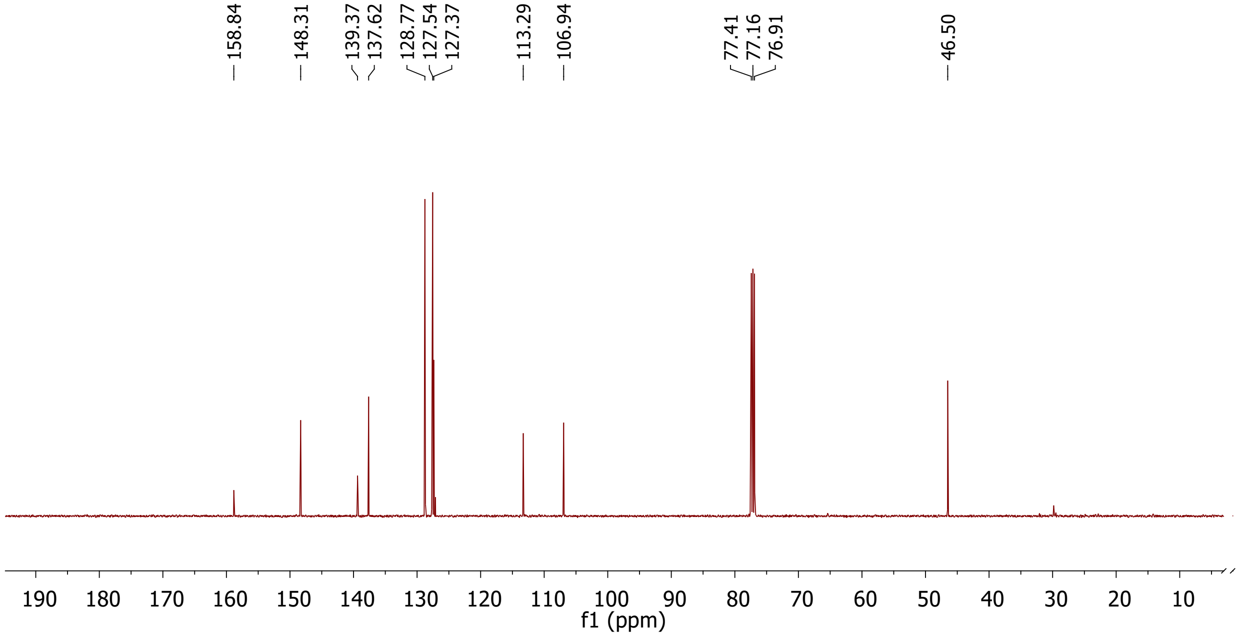

Figure S54. $^{13}C\{^{1}H\}$ NMR (125.7 MHz) spectrum of *N*-benzylpyridin-2-amine in $CDCl_3$.

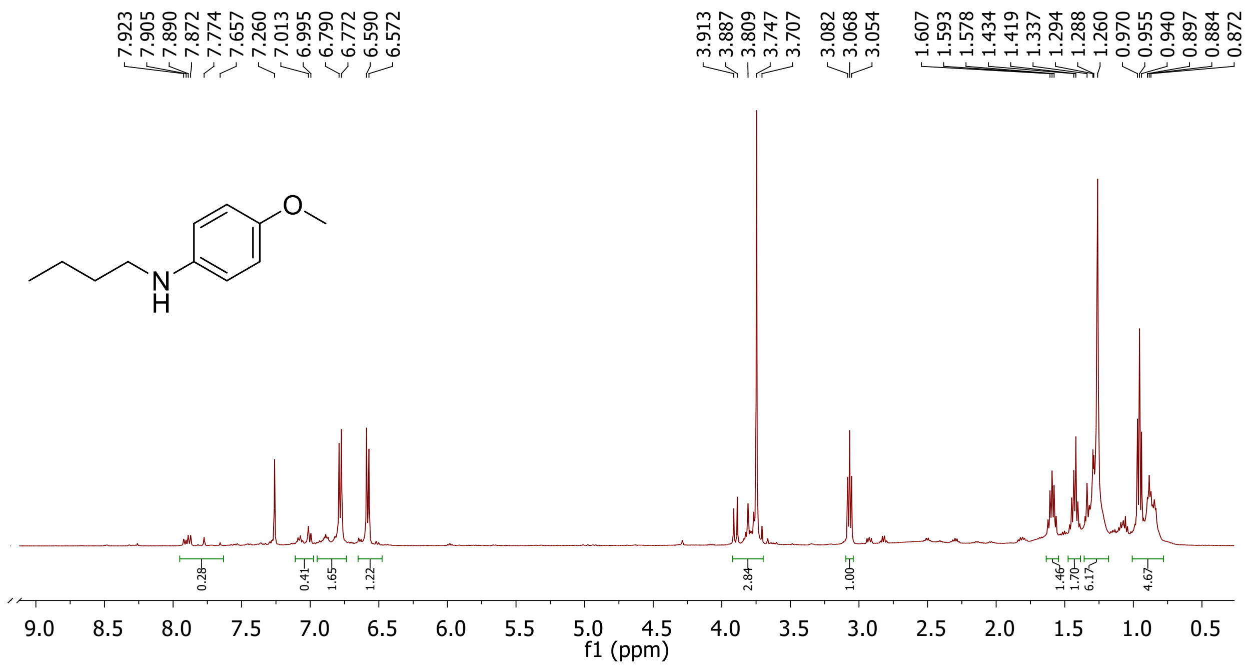

Figure S55. ^{1}H NMR (500 MHz) spectrum of *N*-butyl-4-methoxyaniline in $CDCl_3$.

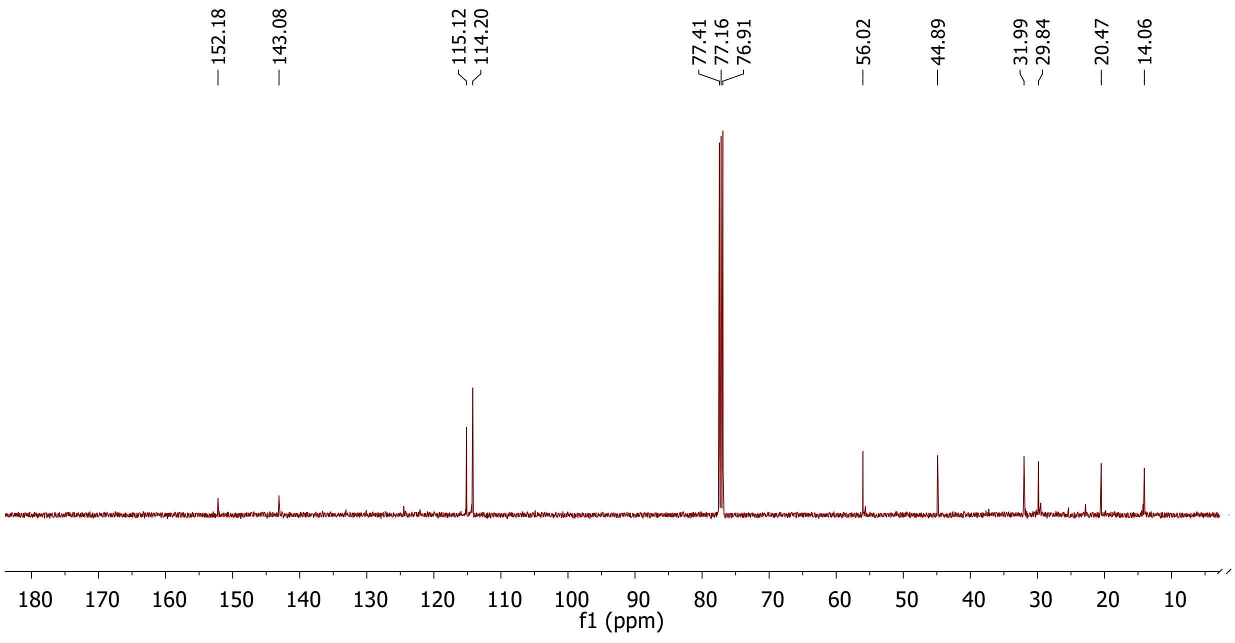

Figure S56. $^{13}C\{^{1}H\}$ NMR (125.7 MHz) spectrum of *N*-butyl-4-methoxyaniline in $CDCl_3$.

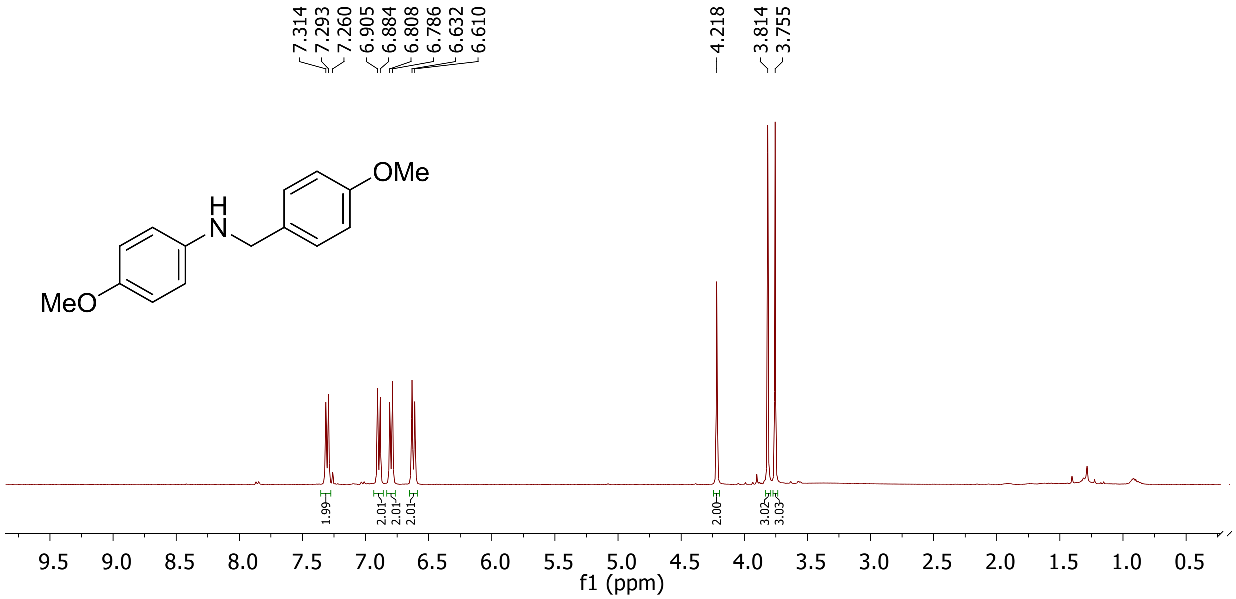

Figure S57: ^{1}H NMR (400 MHz) spectrum of 4-methoxy-*N*-(4-methoxybenzyl)aniline in $CDCl_3$.

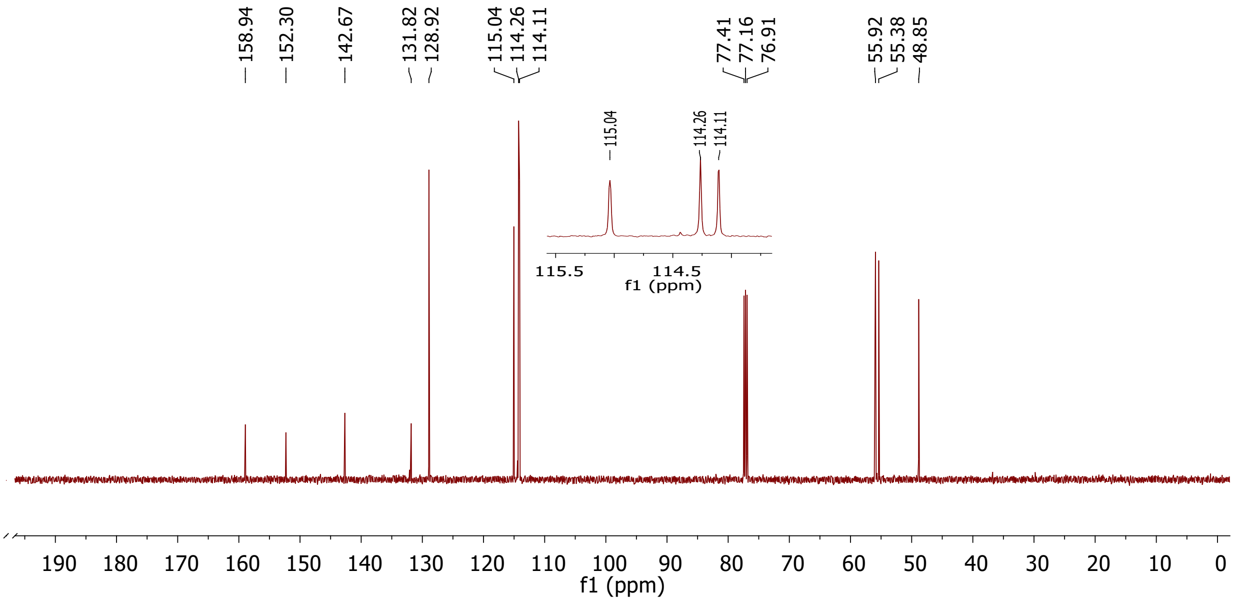

Figure S58: $^{13}C\{^{1}H\}$ NMR (125.7 MHz) spectrum of 4-methoxy-*N*-(4-methoxybenzyl)aniline in $CDCl_3$.

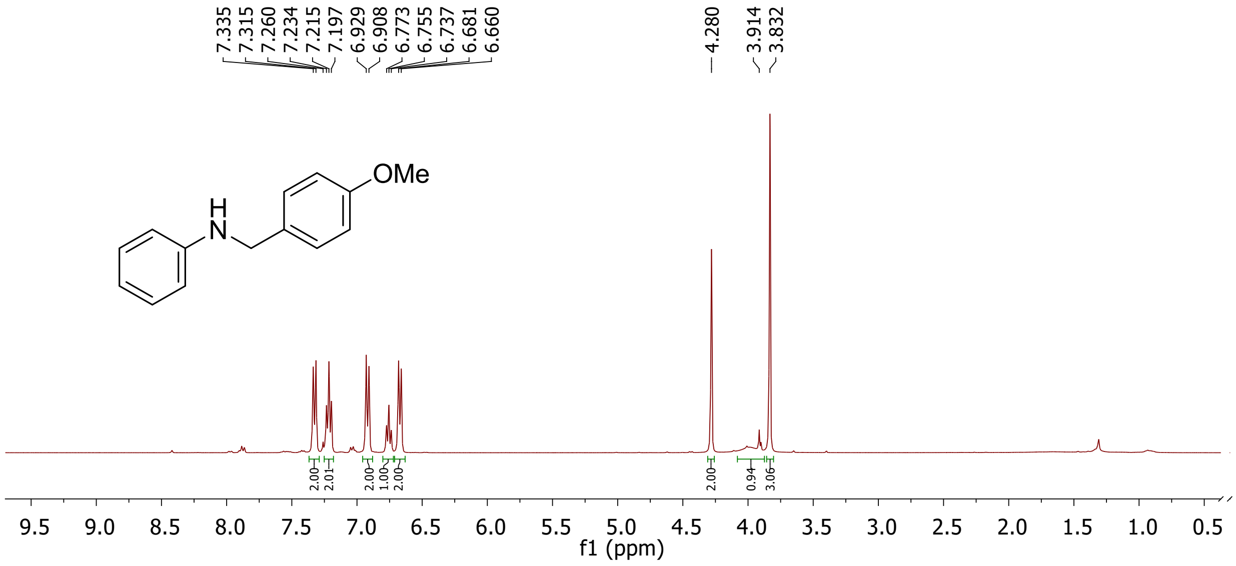

Figure S59: ^{1}H NMR (400 MHz) spectrum of *N*-(4-methoxybenzyl)aniline in $CDCl_3$.

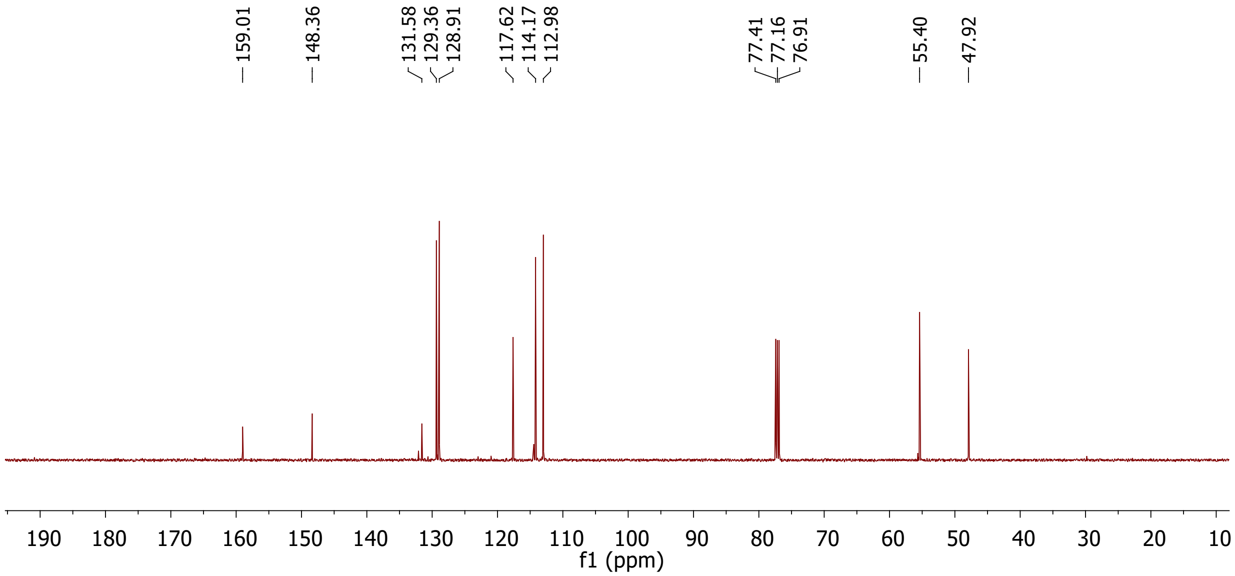

Figure S60: $^{13}C\{^{1}H\}$ NMR (125.7 MHz) spectrum of *N*-(4-methoxybenzyl)aniline in $CDCl_3$.

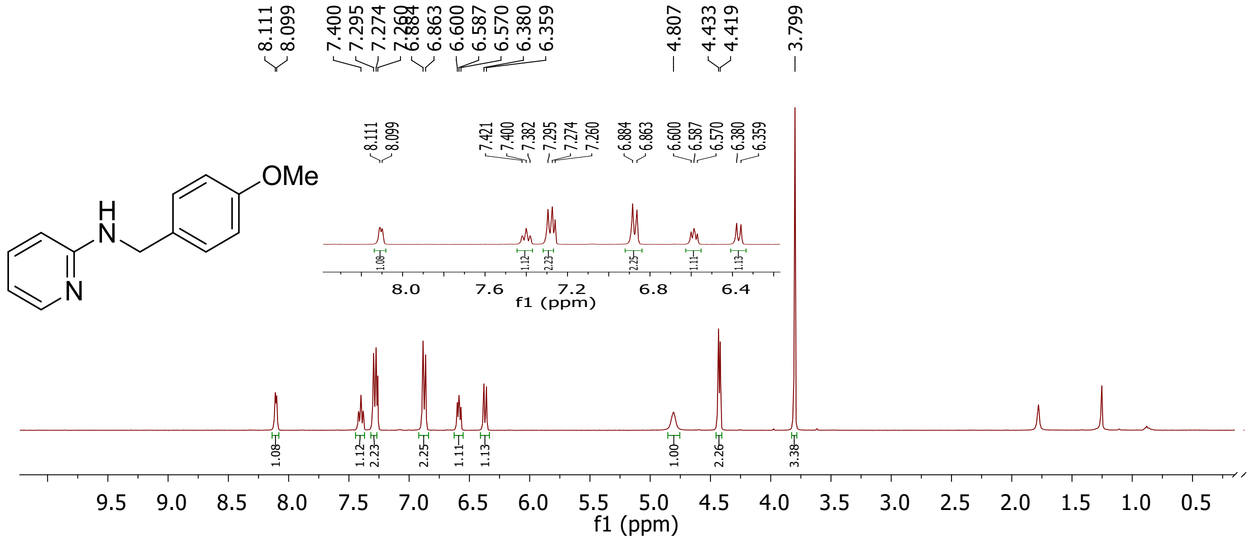

Figure S61: ^{1}H NMR (400 MHz) spectrum of *N*-(4-methoxybenzyl)pyridin-2-amine in $CDCl_3$.

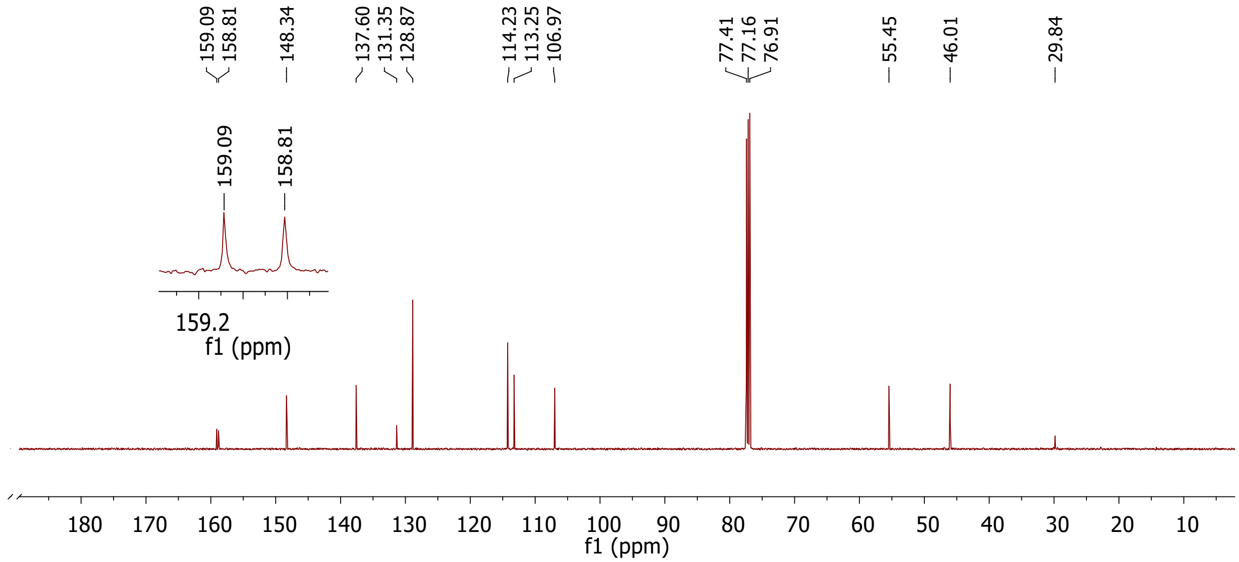

Figure S62: $^{13}C\{^1H\}$ NMR (125.7 MHz) spectrum of *N*-(4-methoxybenzyl)pyridin-2-amine in $CDCl_3$.

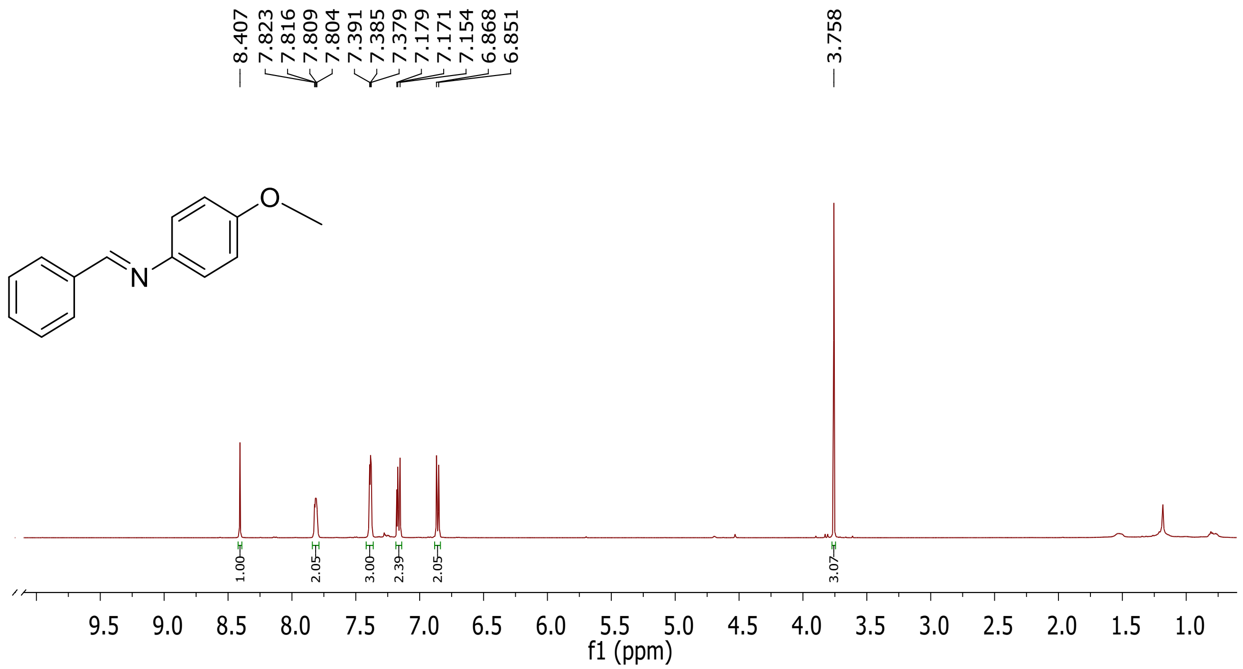

Figure S63: ^{1}H NMR (500 MHz) spectrum of *N*-benzylidene-4-methoxyaniline in $CDCl_3$.

References

1. G. M. Sheldrick, *Acta Cryst. Sect. A: Found. Crystallogr.*, 2015, **71**, 3.
2. G. M. Sheldrick, *Acta Cryst. Sect. C: Struct. Chem.,* 2015, **71**, 3.
